# Mixed-Ligand Complex Formation in the Systems Containing the Potential Drug [V^IV^O(acac)_2_] with Blood and Cellular Bioligands. Implications on the Active Species Formed in the Organism

**DOI:** 10.1021/acs.inorgchem.6c02223

**Published:** 2026-07-22

**Authors:** Daniele Sanna, Valeria Ugone, Péter Buglyó, Nóra Ildikó Kovács, Sándor Nagy, Nádia Ribeiro, Isabel Correia, João Costa Pessoa, Federico Pisanu, Eugenio Garribba

**Affiliations:** † Consiglio Nazionale Delle Ricerche, Istituto di Chimica Biomolecolare, Traversa La Crucca 3, Sassari I-07100, Italy; ‡ Department of Inorganic and Analytical Chemistry, 37599 University of Debrecen, Egyetem tér 1, Debrecen H-4032, Hungary; § Centro de Química Estrutural, Institute of Molecular Sciences and Departamento de Engenharia Química, Instituto Superior Técnico, 72971 Universidade de Lisboa, Av. Rovisco Pais, Lisboa 1049-001, Portugal; ∥ Now at Prof. Silva Pereira’s Applied and Environmental Mycology Group (AEM), Instituto de Tecnologia Química e Biológica (ITQB), Av. da República, Oeiras 2780-157, Portugal; ⊥ Dipartimento di Medicina, Chirurgia e Farmacia, Università di Sassari, Viale San Pietro, Sassari I-07100, Italy

## Abstract

Bis­(acetylacetonato)­oxidovanadium­(IV), [V^IV^O­(acac)_2_], is one of the most widely studied V^IV^ complexes with remarkable biological activities. However, the interaction of [V^IV^O­(acac)_2_] with the serum and cytosol relevant bioligands (bLs) has not been systematically investigated. Here, we present an integrated study of the ternary systems V^IV^O^2+^/acac/bL (where bL is lactate, citrate, oxalate, histidine, cysteine, aspartate, pyrophosphate, ATP, and reduced glutathione) by pH-potentiometry, electrospray ionization-mass spectrometry (ESI-MS), electron paramagnetic resonance (EPR), UV–vis, and circular dichroism (CD). Stability constants were determined for V^IV^O–acac and V^IV^O–acac–bL complexes, showing that significant amounts of mixed-ligand species form in most of the ternary systems, whose distribution is strongly pH- and bL-dependent. EPR measurements allowed obtaining insights into the binding modes of bLs. Mass, UV–vis, and CD spectra confirmed the existence of the proposed ternary species in solution. The thermodynamic data were used to model the speciation of [V^IV^O­(acac)_2_] in the blood serum, in erythrocytes, and cancer cell cytosol, showing that when V concentration is larger than 10–20 μM, [V^IV^O­(acac)­(citrato)]^2–^, [V^IV^O­(acac)­(P_2_O_7_)]^3–^, and, secondarily, [V^IV^O­(acac)­(histidinato)] and [V^IV^O­(acac)­(aspartato)]^−^ become relevant species. The findings, besides clarifying the solution chemistry of [V^IV^O­(acac)_2_] in biologically relevant environments, also highlight the likely role of mixed-ligand V^IV^O­(acac)­(bL) complexes in its transport, accumulation, and pharmacological activity.

## Graphical abstract

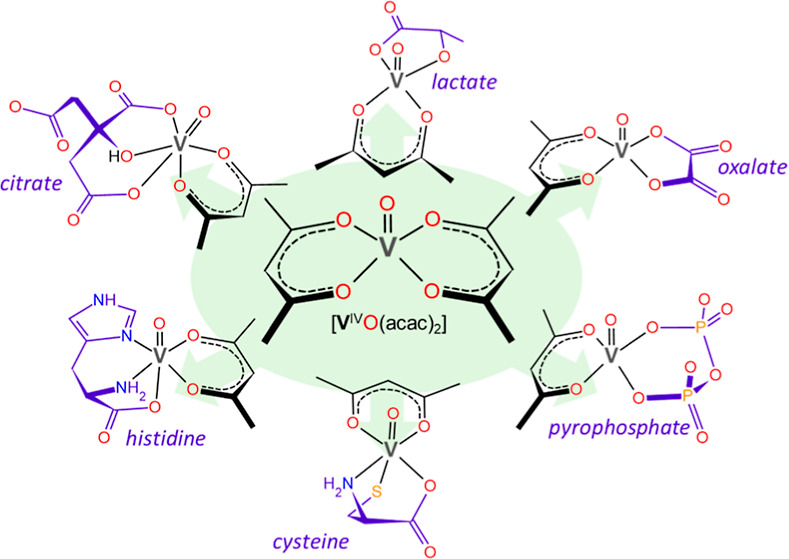

## 1 Introduction

Vanadium compounds (VCs) have been reported to exhibit several biological effects and a wide range of pharmacological properties. They have been proposed for the treatment of many diseases,^1^ and now some of their most promising applications in medicine are their use in the therapy of patients suffering from type 2 diabetes mellitus^1h,2^ and as anticancer,^1c,1f,1h,3^ antibacterial,^1l,4^ or antiparasitic^5^ agents.

Among the compounds with antidiabetic and anticancer prospective use, bis­(acetylacetonato)­oxidovanadium­(IV), [V^IV^O­(acac)_2_] (**1**) is worth being mentioned. In the solid state, it consists of discrete molecules with an almost square pyramidal geometry (Scheme 1).^6^ In the presence of a monodentate ligand L, such as dioxane or methanol, it transforms into *trans*-[V^IV^O­(acac)_2_(L)],^7^ while pyridine, substituted pyridines, quinoline, and isoquinoline may give both *cis*- or *trans*-octahedral isomers (Scheme 1).^7–9^

**Scheme 1:**
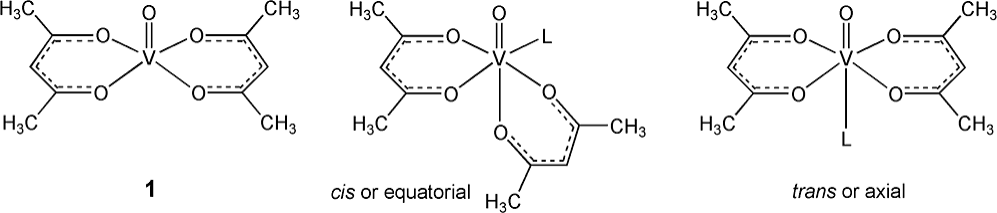
Structural Formulae of [V^IV^O­(acac)_2_] (**1**). In [V^IV^O­(acac)_2_(L)] Complexes, the Donor Atom of Ligand L May Bind *Cis* or *Trans* to the O-Oxido; If the Binding Is *Cis* (or Equatorial), One of the acac^–^ Ligands Will Then Have One of Its O-Atoms Bound *Trans* to the O-Oxido Ligand, While an Equatorial Position Will be Occupied by Ligand L; If the Binding Is *Trans* (or Axial), a Ligand L Can Bind in Axial Position *Trans* to the V=O Group

[V^IV^O­(acac)_2_] is a quite stable V^IV^O species^10^ and has been employed as a precursor in the synthesis of many vanadium complexes where one^11^ or both^5c,12^ acac^–^ ligands are replaced. Moreover, it has been used as a catalyst in reactions to obtain many types of organic compounds.^13^

[V^IV^O­(acac)_2_], and various vanadium-containing species deriving from it, may exhibit several biological effects, namely insulin-enhancing and anticancer properties. It also impacts the cardiac mitochondrial function.^14^ Notably, **1** was reported to ameliorate hyperglycemia in streptozotocin-induced diabetic rats (STZ-rats) more efficiently than V^IV^OSO_4_ ^15^ and to possess a greater capacity to enhance insulin receptor (IR) kinase activity in cells than many other V^IV^O chelate complexes.^10a^ **1** was also shown to have antiproliferative effects,^10a,16^ in particular in human pancreatic cancer cell line AsPC-1 where it induces a G2/M cell cycle arrest through the elevation of reactive oxygen species (ROS) levels. Recently, it was reported that **1** can induce mitotic catastrophe and methuotic cell death in glioblastoma cells, an aggressive form of cancer with limited types of clinical treatment; [V^IV^O­(acac)_2_] markedly inhibits the growth of glioblastoma cells in both in vitro and in vivo models, showing, with an IC_50_ of 4–6 μM, a greater activity than the standard treatment with temozolomide.^17^ Notably, **1** shows low cytotoxicity on normal cells, such as primary neural cells, astrocytes, and BV2 microglia cells with selectivity indices in the range 9.7–106.0.^17^ Moreover, it was demonstrated by Makinen and Mustafi and their groups, through positron emission tomography imaging and X-ray fluorescence microscopy, that **1** preferentially accumulates in human breast carcinoma or murine colon cancer cells than in normal cells.^18^ These results indicate that V-based compounds may have a potential application as both antidiabetic and anticancer agents to prevent or treat patients suffering from both diseases.^16,19^

Another remarkable property of [V^IV^O­(acac)_2_] is its high activity in degrading plasmid DNA in the absence of any activating agents, air, and photoirradiation.^20^ The nature of the pH buffer is relevant for the nuclease activity, and in phosphate-buffered medium, single-strand cleavage is clear for concentrations of **1** as low as 1.2 μM. Moreover, in the presence of activators, the cleavage is still much more effective.^20^

Depending on experimental conditions, such as pH and temperature, and on the presence and strength of other ligands, complexes of labile metal ions undergo changes when dissolved in water-containing media, namely hydrolysis and ligand exchange. Vanadium compounds have been discussed in this context and may additionally be involved in redox reactions, V^III^, V^IV^, and V^V^ being the most common oxidation states.^7,21^ The biotransformation of species with composition V^IV^O­(L)_2_ largely affects the pharmacological efficacy by determining the absorption in the gastrointestinal tract, the transport in the blood, the uptake by the target cells, and—overall—the availability of the pharmacologically active species.^22^ Depending on the thermodynamic stability, particularly under physiological conditions, the interaction with blood and cellular bioligands (bLs) and proteins can take place with the formation of mixed-ligand species V^IV^O–acac–bL/protein or the complete replacement of acac^–^ by bL or proteins.^7,23^ Such types of interactions seem to play a key role in the transport and mechanism of action of pharmacologically active VCs and have been investigated by several groups.^24,25^ The possibility that [V^IV^O­(acac)_2_] remains intact or form mixed-ligand species in the bloodstream and in the cellular environment could be indicated by the dose-dependent capacity to lower plasma glucose in diabetic laboratory animals, and by long lifetime in the blood, which allows one to find a correlation of its pharmacological action with vanadium content in blood and cells.^10a,26^ Moreover, the neutral form of **1** could underlie its relatively high cell accumulation, much greater than that of [V^IV^O­(ma)_2_], with ma = maltolato and vanadate­(V).^27^

Some authors stated that [V^IV^O­(acac)_2_] binds ligands in the *trans* position,^28^ and others that the coordination to the V^IV^ occurs only equatorially, while other publications report either *cis*- or *trans*-binding depending on the ligand added (Scheme 1).^9,29^ In some cases, the axial interaction (e.g., H-bonding) with the O-oxido ligand of another molecule was also proposed.^30^ This is an important aspect because the geometry assumed in solution affects the way it may bind to biological molecules, e.g., a protein.^7,31^ The binding mode of the ligands to *cis* and *trans* isomers of V^IV^O­(acac)_2_ is prevalently governed by steric issues,^9^ with the formation of the adducts occurring without loss of stability compared to **1**.

It has been demonstrated that V^IV^O–acac complexes bind to serum proteins, namely human apo-transferrin (apo-HTf) forming [V^IV^O­(acac)_2_(apo-HTf)] and/or [V^IV^O­(acac)­(apo-HTf)] adducts.^32^ If V^IV^O–acac species are uptaken by cells, it is also relevant to evaluate its potential to bind to molecules present inside them. Even though it has been emphasized that **1** is a stable entity,^10c,24e,33^ it is a weak Lewis acid and, upon dissolution in solutions containing potential ligands, the vanadium center may coordinate a donor ligand in the vacant site, forming [V^IV^O­(acac)_2_(L)] complexes, and this process (eq 1) has been studied by several methods.^10b,28,29,34^

1 $$\left[V^{IV}O{\left(acac\right)}_{2}\right]+L⇄\left[V^{IV}O{\left(acac\right)}_{2}\left(L\right)\right]$$

There are many studies confirming the formation of mixed-ligand complexes with composition [V^IV^O­(acac)_2_(L)] or [V^IV^O­(acac)­(L–L)], where L is a monodentate and L–L are bi- or tridentate ligands. Furthermore, the formation of complexes such as [V^IV^O­(acac)­(L^1^, L^2^)] or [V^IV^O­(acac)­(L^1^, L^2^, L^3^)], where L^1^, L^2^, and L^3^ are donor atoms from proteins is plausible and, indeed, the formation of V^IV^O–acac–apo-HTf adducts or V^IV^O–acac–Lyz/Ub (Lyz = lysozyme, Ub = ubiquitin) was demonstrated through spectrometric, spectroscopic, and computational techniques.^32,35^

Until now, however, the interaction of [V^IV^O­(acac)_2_] with the most important small bioligands of blood and cells was not studied and no data on the possible formation of mixed-ligand species were reported, unlike other potential V-based drugs such as [V^IV^O­(pic)_2_(H_2_O)],^36^ [V^IV^O­(ma)_2_],^37^ and [V^IV^O­(dhp)_2_],^38^ where pic = picolinate, and dhp = 1,2-dimethyl-3-hydroxy-4­(1*H*)­pyridinonate, whose behavior has been discussed in some reviews.^39,40^ In light of what was discussed in the literature for pic, ma, and dhp, these mixed complexes could be important to explain the transport to the target organs and the mechanism of action of [V^IV^O­(acac)_2_]. In this work, the ternary systems V^IV^O^2+^/acac/bL, where bL is a serum or cellular bioligand (lactate (lact), citrate (citr), oxalate (ox), histidine (His), cysteine (Cys), aspartate (Asp), pyrophosphate, adenosine triphosphate (ATP), and reduced glutathione (GSH)), were studied through the combined application of potentiometric (pH-titrations), spectrometric (electrospray ionization-mass spectrometry (ESI-MS)), and spectroscopic techniques (electron paramagnetic resonance (EPR), UV–vis spectroscopy and circular dichroism (CD)), these techniques providing valuable information on the coordination mode of the ligands around V^IV^O^2+^ ions.^31,41–43^ The bioligands were selected both for their high concentration in the blood serum or cytosol and for their binding strength, as they are capable of replacing one or both acac^–^ anions, forming mixed-ligand V^IV^O–acac–bL or the corresponding binary V^IV^O–bL complexes. The studied bLs are also known to have relevant roles in the metabolism of cells.^44^ In fact, the concentration of some of them significantly increases in the cytosol of cancer cells when compared with normal ones (e.g., lactate, oxalate, aspartate, cysteine, pyrophosphate, ATP, and glutathione), while for others a decrease is observed (citrate and histidine).

The data obtained may help to disclose the distribution of V^IV^O–acac species among the bioligands of blood and cellular environment.

## 2 Experimental Section

### 2.1 Chemicals

V^IV^OSO_4_·3H_2_O, acetylacetone, [V^IV^O­(acac)_2_], lactic acid, citric acid, oxalic acid, l-aspartic acid, l-histidine, l-cysteine, pyrophosphoric acid, adenosine triphosphate, reduced glutathione, 1-methylimidazole (MeIm), and ammonium carbonate were all Sigma-Aldrich products and were used without further purification. V^IV^O^2+^ solutions were prepared according to literature procedures.^45^

### 2.2 EPR Measurements

The solutions for EPR experiments were prepared dissolving a weighted amount of V^IV^OSO_4_·3H_2_O, acac, and the bioligands in ultrapure water (obtained through the purification system Millipore Milli-Q Academic), to obtain a metal ion concentration of 2.0 × 10^–3^ M and different bioligand-to-metal molar ratios (bL:M). Argon was bubbled through the solutions to ensure the absence of oxygen and avoid the oxidation of V^IV^. Immediately after the preparation of the solutions, samples were frozen at 120 K with liquid nitrogen and EPR spectra recorded at different pH values.

EPR spectra were recorded at 120 K with an X-band (9.4 GHz) Bruker EMX spectrometer equipped with an HP 53150A microwave frequency counter with this instrumental setting: microwave frequency 9.40–9.41 GHz, microwave power 20 mW, time constant 81.92 ms, modulation frequency 100 kHz, modulation amplitude 0.4 mT, resolution 4096 points. When the signal-to noise-ratio was small due to the low V^IV^O^2+^ concentration, signal averaging was used; this process involves the repeated acquisition of *N* spectra, which results in an increase of the signal-to-noise ratio of a factor √*N*.^46^ The hyperfine coupling constants along the *z* axis, *A*_*z*_, for V^IV^O complexes are negative, so in the text the absolute values are reported (|*A*_*z*_|). For some systems, the experimental values of |*A*_*z*_| (|*A*_*z*_|^exptl^) were compared with those estimated with the “additivity relationship” (|*A*_*z*_|^estmtd^), which relates |*A*_*z*_| to the number and type of the equatorial ligands, assigning them a specific contribution. This empirical rule has been proved and accepted in a large number of articles.^47^ Usually, the experimental values of |*A*_*z*_| fall in the range of ±3 × 10^–4^ cm^–1^ with respect to those estimated with the “additivity relationship”.^47a,47b^ The contribution of the various donors was taken from refs 47b,47c,48 .

### 2.3 ESI-MS Measurements

The solutions were prepared dissolving V^IV^OSO_4_·3H_2_O and acac, or directly [V^IV^O­(acac)_2_], in ultrapure water (LC–MS grade, Sigma-Aldrich) in order to obtain metal concentrations in the range 0.5–1.0 × 10^–3^ M and a bL:M ratio of 1:1 or 2:1. In these solutions, the bioligand bL was added to obtain different V^IV^O^2+^:acac:bL molar ratios. The pH was adjusted by adding a diluted solution of (NH_4_)_2_CO_3_ and argon was bubbled through the solutions to ensure the absence of O_2_, avoiding the oxidation of V^IV^O^2+^ ions, and the removal of any CO_2_ formed from ammonium carbonate. An aliquot of each of the final solutions was diluted to have a vanadium concentration (*c*_V_) of 5.0 × 10^–6^ M, the pH was set to the desired value, and the ESI-MS spectra were recorded.

Mass spectra in positive-ion (ESI-MS­(+)) and negative-ion (ESI-MS(−)) modes were obtained on a Q Exactive Plus Hybrid Quadrupole-Orbitrap (Thermo Fisher Scientific) mass spectrometer. The solutions were infused at a flow rate of 5.00 μL/min into the ESI chamber immediately after their preparation. The spectra were recorded in the *m*/*z* range 50–750 at a resolution of 140,000. The experimental conditions used for the ESI-MS­(+) measurements were as follows: spray voltage 2300 V, capillary temperature 250 °C, sheath gas 5–10 (arbitrary units), auxiliary gas 3 (arbitrary units), sweep gas 0 (arbitrary units), and probe heater temperature 50 °C. For the ESI-MS(−) measurements, the conditions were as follows: spray voltage −1900 V, capillary temperature 250 °C, sheath gas 20 (arbitrary units), auxiliary gas 5 (arbitrary units), sweep gas 0 (arbitrary units), and probe heater temperature 14 °C. MS/MS experiments were performed using NCE setting in the range 10–40 (arbitrary units); ion fragments were detected at a resolution of 17,500. The spectra were analyzed by using Thermo Xcalibur 3.0.63 software (Thermo Fisher Scientific).

### 2.4 Circular Dichroism and Visible Absorption Spectra Measurements

Circular dichroism and visible absorption spectra were recorded as a function of pH and metal-to-bioligand molar ratio from stock solutions of [V^IV^O­(acac)_2_] and of bioligands. The stock solution of [V^IV^O­(acac)_2_] contained 12.5% (v/v) MeOH to facilitate its dissolution and prevent the oxidation of V^IV^ to V^V^. The pH was varied adding diluted solutions of HCl and NaOH, and the explored pH range was 4–11, depending on the bioligand. The value of *c*_V_ was between 2 and 4 mM and V^IV^O^2+^:bL ratios were 1:0, 1:0.5, 1:1, 1:2, 1:4, 1:6, or 1:12. The visible absorption spectra were normally recorded in 1.0 cm quartz cells between 400 and 900 nm with a PerkinElmer Lambda 35 UV–vis spectrophotometer, while the circular dichroism spectra were obtained in either 1.0 or 2.0 cm quartz cells, normally in the 400–800 nm wavelength range, with a JASCO 720 spectropolarimeter, with the photomultiplier suitable for the 400–900 nm range. Note that, while the V^IV^O species absorb radiation of the visible range of the spectrum, the chiral ligands involved in this study do not absorb. Therefore, it is the chirality of the ligand atoms that is somewhat transmitted to the V^IV^ atom, and the CD bands observed correspond to what is designated by induced CD spectra. Unless otherwise stated, by CD spectra we mean a representation of Δε values vs λ, where Δε corresponds to the differential absorption/(*b* × *c*_V_), with *b* being the optical path of the quartz cell and *c*_V_ the total concentration of the chosen species, in this study the added V^IV^O compound. While for the visible absorption spectra all species absorbing at the particular wavelengths measured contribute (according to their concentration and ε values) to the CD spectra, this is true only for chiral species.^41^ Therefore, when present in solution, V^IV^O complexes such as [V^IV^O­(acac)_2_], [V^IV^O­(acac)­(H_2_O)_2_]^+^, and [V^IV^O­(H_2_O)_5_]^2+^, correspond to Δε = 0 and do not contribute to the measured CD spectra.

### 2.5 Potentiometric Measurements

Protonation constants of the ligands and stability constants of their V^IV^O complexes were determined by pH-potentiometric titrations on 15.00 mL of samples at 25.0 ± 0.1 °C and at a constant ionic strength of 0.20 M (KCl). The V^IV^O^2+^ concentration was in the range 0.9–2.2 × 10^–3^ M. In the case of V^IV^O^2+^/acac and V^IV^O^2+^/Asp systems 1:1, 1:2, and 1:4 molar ratios were explored, while for the ternary systems V^IV^O^2+^/acac/lact, V^IV^O^2+^/acac/citr, V^IV^O^2+^/acac/His, V^IV^O^2+^/acac/Asp, V^IV^O^2+^/acac/H_4_P_2_O_7_, and V^IV^O^2+^/acac/ATP 1:1:1, 1:2:1, 1:1:2, and 1:2:2 molar ratios were used. All titrations were performed over the pH range 2.0–11.0, or until precipitation occurred with a carbonate-free KOH of known concentration (ca. 0.2 M) under a purified argon atmosphere. A Mettler Toledo T50 titrator equipped with a combined glass electrode (type 6.0234.100) was used for the pH-metric measurements. The electrode system was calibrated according to Irving et al.,^49^ and therefore the pH-metric readings were converted into hydrogen-ion concentrations. The water ionization constant, p*K*_w_, was 13.76 ± 0.01 under the conditions used. A 10 min waiting time was employed during the titrations to reach steady pH readings. If this time was not enough to obtain pH equilibrium, the data points were omitted in the calculations. The reproducibility of the points included in the evaluation was within 0.005 pH unit in the whole pH range measured. The number of experimental points used in the calculations was about 200–300. The stability of the ternary complexes, reported as the logarithm of the overall formation constant β_pqrs_ = [(VO)_p_(acac)_q_(bL)_r_H_s_]/[VO]^p^[acac]^q^[bL]^r^[H]^s^, where VO stands for the V^IV^O^2+^ ion and acac and bL are the deprotonated forms of acetylacetone and bioligands (lact^–^, citr^3–^, His^–^, Asp^2–^, P_2_O_7_^4–^, and ATP^4–^), was calculated with the aid of the PSEQUAD program.^50^ The stability constants for the binary species formed in the V^IV^O^2+^/lact, V^IV^O^2+^/citr, V^IV^O^2+^/His, V^IV^O^2+^/Asp, V^IV^O^2+^/H_4_P_2_O_7_, and V^IV^O^2+^/ATP systems were taken from the literature^51^ and used as fixed values to estimate those of the ternary complexes. The notation H_–1_ indicates proton dissociation of groups which do not deprotonate in the absence of V^IV^O^2+^ coordination or hydroxido ligands. The uncertainties (3σ values) of the stability constants are given in parentheses. During the calculations the following hydroxido complexes of V^IV^O^2+^ were assumed: [V^IV^O­(OH)]^+^ (logβ_100–1_ = −5.94), [(V^IV^O)_2_(OH)_2_]^2+^ (logβ_200–2_ = −6.95), with stability constants calculated from the data of Henry et al.^52^ and corrected for the different ionic strengths by use of the Davies equation,^53^ [V^IV^O­(OH)_3_]^−^ (logβ_100–3_ = −18.0) and [(V^IV^O)_2_(OH)_5_]^−^ (logβ_200–5_ = −23.5).^10b,54^

The distribution curves and calculated speciation in the blood serum, RBC cytosol, and cancer cells were generated using the suite of programs Aq_Solutions.^55^

## 3 Results and Discussion

### 3.1 Binary System V^IV^O^2+^/acac

[V^IV^O­(acac)_2_] is one of the most remarkable and stable oxidovanadium­(IV) complexes known,^10a,15b^ with a wide spectrum of biological activity. In the solid state, it presents an almost regular square pyramidal geometry (Scheme 1).^6b,10a,10c^ The reported stability constants in aqueous solution for the formation of V^IV^O­(acac)^+^ and V^IV^O­(acac)_2_ are logβ_1_ = 8.73 and logβ_2_ = 16.27, respectively.^10c^ EPR spectroscopy allowed one to characterize the 1:1 and 1:2 species with *g*_*z*_ = 1.940, |*A*_*z*_| = 174.5 × 10^–4^ cm^–1^ (V^IV^O­(acac)^+^) and *g*_*z*_ = 1.946, |*A*_*z*_| = 166.7 × 10^–4^ cm^–1^ (V^IV^O­(acac)_2_).^28b^

In this study, the characterization of the systems V^IV^O^2+^/acac was re-examined by pH-metric titrations and ESI-MS spectrometry (Table 1). pH titrations indicate that three species are formed: [V^IV^O­(acac)­(H_2_O)_2_]^+^, [V^IV^O­(acac)_2_], and [(V^IV^O)_2_(acac)_2_(OH)_2_]. The stability constants of [V^IV^O­(acac)­(H_2_O)_2_]^+^ and [V^IV^O­(acac)_2_] are comparable with those reported in the literature,^10c^ and the formation of [(V^IV^O)_2_(acac)_2_(OH)_2_] (indicated with [(V^IV^O)_2_(acac)_2_H_–2_] by potentiometry) accounts for the deprotonation, and the decrease of the EPR signal observed at pH > 6. The formation of [(V^IV^O)_2_(acac)_2_(OH)_2_] can be explained with the hydrolysis of [V^IV^O­(acac)­(H_2_O)_2_]^+^, which yields a di-μ-hydroxido-bridged species due to the reaction 2 [V^IV^O­(acac)­(H_2_O)_2_]^+^ ⇄ [(V^IV^O)_2_(acac)_2_(μ-OH)_2_] + 2H^+^.^56^ As many other di-μ-hydroxido dinuclear V^IV^O species, [(V^IV^O)_2_(acac)_2_(OH)_2_] shows an antiferromagnetic behavior, due to an *anti*- or a *syn*-orthogonal arrangement of the V=O group with respect to the bridging plane^57^ and cannot be revealed by EPR. The calculated species distribution diagram is shown in Figure 1.

**Table 1:** Proton and V^IV^O Stability Constants of the Species Formed in the Binary System with Acetylacetone and in the Ternary Systems with the Studied Bioligands

	acac^–^	lact^–^ ^a^	citr^3–^ ^b^	His^–^ ^c^	Asp^2–^ ^d^	P_2_O_7_^4–^ ^e^	ATP^4–^ ^f^
Hacac	8.81(1)						
HbL		3.70(4)	5.55(1)	9.13(1)	9.64(1)	8.25(1)	6.45(1)
H_2_bL		–	9.85(1)	15.19(1)	13.29(1)	14.13(1)	10.35(1)
H_3_bL		–	12.80(1)	16.87(1)	15.12(2)	15.79(1)	–
[V^IV^O(acac)(H_2_O)_2_]^+^	8.73(1)						
[V^IV^O(acac)_2_]	16.05(3)						
[(V^IV^O)_2_(acac)_2_H_–2_]^g^	9.47(10)						
V^IV^O(acac)(H_2_bL)		–	22.36(4)	–	–	26.4(2)	–
V^IV^O(acac)(HbL)		–	19.74(3)	–	20.91(9)	24.38(4)	18.95(4)
V^IV^O(acac)(bL)		11.87(4)	16.66(3)	17.80(4)	16.46(9)	18.70(9)	14.00(7)
number of points		313	338	290	231	301	238
fitting (mL)		1.9 × 10^–2^	1.3 × 10^–2^	2.5 × 10^–2^	1.2 × 10^–2^	2.1 × 10^–2^	1.2 × 10^–2^
maximum pH		6.5	9.0	9.5	8.0	9.0	7.0

a Hlact is the neutral form.

b H_3_Citr is the neutral form.

c HHis is the neutral form.

d H_2_Asp is the neutral form.

e H_4_P_2_O_7_ is the neutral form.

f H_4_ATP is the neutral form.

g The notation H_–2_ indicates the dissociation of two groups which do not deprotonate in the absence of V^IV^O^2+^ coordination. The formation of this species can be explained with the hydrolysis of [V^IV^O­(acac)­(H_2_O)_2_]^+^, which gives a di-μ-hydroxido-bridged complex, [(V^IV^O)_2_(acac)_2_(OH)_2_]. In the rest of the text, the formula [(V^IV^O)_2_(acac)_2_(OH)_2_] is used for clarity.

**Figure 1:**
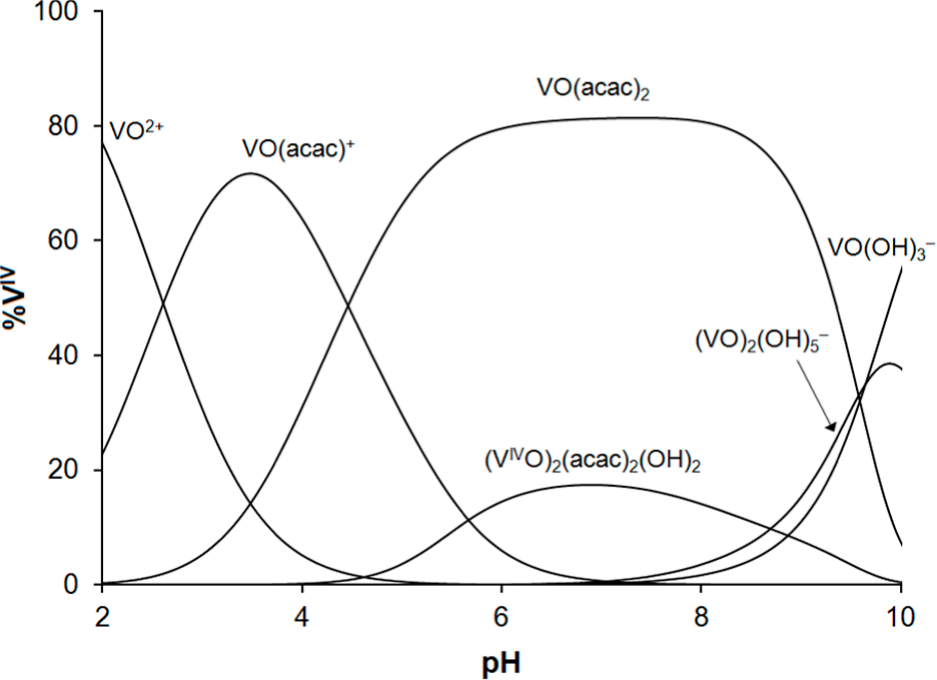
Concentration distribution curves of the V^IV^O species formed as a function of pH in the V^IV^O^2+^/acac system with molar ratio 1:2 and *c*_V_ = 2.0 × 10^–3^ M.

In the ESI-MS­(+) spectrum of [V^IV^O­(acac)_2_], the following species were identified: [V^IV^O­(acac)_2_+H]^+^ (*m*/*z* = 266.0355) and [V^IV^O­(acac)_2_+Na]^+^ (*m*/*z* = 288.0175) in the positive ion mode and [V^IV^O­(acac)_2_–H]^−^ (*m*/*z* = 264.0212, with low intensity) in the negative ion mode. The spectrum in the positive mode is shown in Figure S1 of the Supporting Information. The comparison between the experimental and calculated isotopic pattern for the peak of [V^IV^O­(acac)_2_+Na]^+^ ion is shown as an example in Figure S2 of the Supporting Information. In the ESI-MS(−) spectrum, the signal of vanadate ion [H_2_V^V^O_4_]^−^ at *m*/*z* 116.9388 was also detected; this could be a product of the oxidation of hydrolytic V species present in solution and/or formed during the ESI-MS experiment. The relevant species derived from ligands, free metal ions, and binary V^IV^O complexes identified by ESI-MS spectrometry are listed in Table S1 of the Supporting Information.

### 3.2 Ternary System V^IV^O^2+^/acac/lact

The potentiometric titrations of the ternary system V^IV^O^2+^/acac/lact indicates the formation of one mixed-ligand species between pH 3 and 6 with composition [V^IV^O­(acac)­(lact)] that reaches ca. 40% of total V at mM concentration range. The species distribution diagram of the formed species is reported in Figure 2. No evidence of formation of [V^IV^O­(acac)­(lactH_–1_)]^−^, with the deprotonation of alcoholic OH group, is obtained.

**Figure 2:**
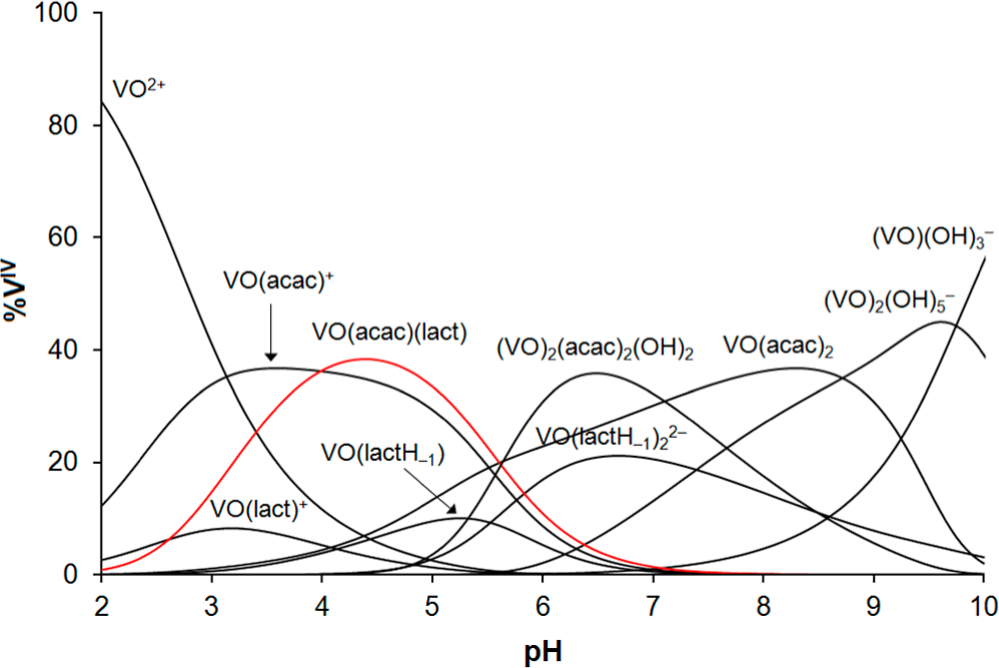
Concentration distribution curves of the species formed as a function of pH in the system V^IV^O^2+^/acac/lact with molar ratio 1:1:1 and *c*_V_ = 2.0 × 10^–3^ M. The curve of the mixed-ligand species is represented in red.

EPR spectra of solutions containing V^IV^O^2+^, acac, and lact with a molar ratio 1:1:1 were recorded at different pH values (*c*_V_ = 2.0 mM). A mixed-ligand species is formed in solution at acidic pH, with a maximum concentration around 4.5, confirming the pH-potentiometric results; its spin Hamiltonian parameters, *g*_*z*_ = 1.943 and |*A*_*z*_| = 173.5 × 10^–4^ cm^–1^, and its composition is [V^IV^O­(acac)­(lact)] (**II** in Figure S3 of the Supporting Information). At pH 7.4, [V^IV^O­(acac)_2_] (**III**) is the major species and [V^IV^O­(lactH_–1_)_2_]^2–^ (**IV**) is a minor complex, in agreement with the potentiometric data, considering that [(V^IV^O)_2_(acac)_2_(OH)_2_] is EPR-silent.

ESI-MS­(+) and ESI-MS(−) spectra of the system V^IV^O^2+^/acac/lact with a molar ratio 1:2:2 and 1:1:1 were recorded at pH around 5 (Figure S4 of the Supporting Information) and the following species were identified: [V^IV^O­(acac)­(lact)+H]^+^ (*m*/*z* = 256.0146), [V^IV^O­(acac)­(lact)+Na]^+^ (*m*/*z* = 277.9968) in the positive ion mode, [V^IV^O­(acac)­(lact)–H]^−^ (*m*/*z* = 254.0002) and [V^V^O_2_(lact)–H]^−^ (*m*/*z* = 170.9497) in the negative ion mode. The spectra do not change significantly with the molar ratio. The experimental and calculated isotopic pattern for the peak of [V^IV^O­(acac)­(lact)–H]^−^ in the ESI-MS(−) spectrum are shown in Figure S5 of the Supporting Information. The list with the mixed-ligand complexes revealed by ESI-MS is reported in Table 2.

**Table 2:** Mixed-Ligand Species Identified by ESI-MS Spectrometry in the Studied Systems

ion	composition	exptl *m/z* ^a^	calcd *m*/*z* ^a^	error (ppm)^b^
[V^IV^O(acac)(lact)+H]^+^	C_8_H_13_O_6_V	256.0148	256.0146	0.6
[V^IV^O(acac)(lact)+Na]^+^	C_8_H_12_O_6_VNa	277.9968	277.9966	0.8
[V^IV^O(acac)(lact)–H]^−^	C_8_H_11_O_6_V	254.0002	254.0001	0.6
[V^IV^O(acac)(citr)+3H]^+^	C_11_H_15_O_10_V	358.0102	358.0099	0.7
[V^IV^O(acac)(citr)+2H+Na]^+^	C_11_H_14_O_6_VNa	379.9923	379.9919	1.1
[V^IV^O(acac)(citr)+H]^−^	C_11_H_13_O_10_V	355.9959	355.9954	1.5
[V^IV^O(acac)(ox)]^−^	C_7_H_7_O_7_V	253.9638	253.9637	0.6
[V^IV^O(acac)(His)+H]^+^	C_11_H_16_O_5_N_3_V	321.0527	321.0524	0.7
[V^IV^O(acac)(His)+Na]^+^	C_11_H_15_O_5_N_3_VNa	343.0346	343.0344	0.6
[V^IV^O(acac)(His)+K]^+^	C_11_H_15_O_5_N_3_VK	359.0091	359.0088	0.6
[V^IV^O(acac)(Cys)+2H]^+^	C_8_H_14_NO_5_SV	287.0027	287.0027	0.1
[V^IV^O(acac)(Cys)+H+Na]^+^	C_8_H_13_NO_5_SVNa	308.9847	308.9846	0.4
[V^IV^O(acac)(Cys)]^−^	C_8_H_12_NO_5_SV	284.9884	284.9881	0.8
[V^IV^O(acac)(Asp)+2H]^+^	C_9_H_14_O_7_NV	299.0209	299.0204	1.4
[V^IV^O(acac)(Asp)+H+Na]^+^	C_9_H_13_O_7_NVNa	321.0026	321.0024	0.5
[V^IV^O(acac)(Asp)]^−^	C_9_H_12_O_7_NV	297.0061	297.0059	0.8
[V^IV^O(acac)(P_2_O_7_)+2H]^−^	C_5_H_7_O_10_P_2_V	341.9119	341.9116	1.0
[V^IV^O(acac)(ATP)+H]^2–^	C_15_H_20_O_16_N_5_P_3_V	334.9789	334.9784	1.3
[V^IV^O(acac)(GSH)+3H]^+^	C_15_H_24_N_3_O_9_SV	473.0669	473.0667	0.4
[V^IV^O(acac)(GSH)+2H+Na]^+^	C_15_H_24_N_3_O_9_SVNa	495.0489	495.0487	0.5

a The experimental and calculated *m*/*z* values refer to the representative peak with monoisotopic mass.

b Deviation in ppm from the calculated values, determined as 10^6^ × [(Exptl *m*/*z* – Calcd *m*/*z*)/Calcd *m*/*z*].

The structure of the possible mixed-ligand species formed by the V^IV^O­(acac)^+^ moiety with lactate, [V^IV^O­(acac)­(lact)], is represented in Scheme 2A.

**Scheme 2:**
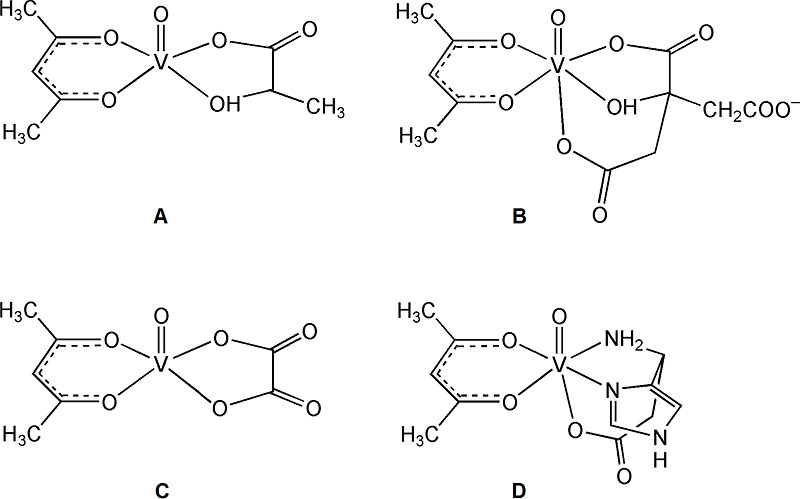
Proposed Structures of the Most Relevant Mixed-Ligand Species Formed in the Ternary Systems with Lact, Citr, Ox, and His: (A) [V^IV^O­(acac)­(lact)]; (B) [V^IV^O­(acac)­(citr)]^2–^; (C) [V^IV^O­(acac)­(ox)]^−^; and (D) [V^IV^O­(acac)­(His)]

UV–vis absorption and circular dichroism spectra were recorded with solutions of [V^IV^O­(acac)_2_] adding different amounts of lactic acid and adjusting the pH at 6.0 with HCl or NaOH. The changes in ε values in the visible absorption spectra indicate that mixed-ligand complexes are formed at this pH, in agreement with potentiometric, EPR, and ESI-MS data (Figure S6 of the Supporting Information). The CD spectra confirm these results and indicate that at least one V^IV^O–acac–lact species forms at pH = 6.0.

### 3.3 Ternary System V^IV^O^2+^/acac/citr

The pH-potentiometric measurements allow to detect three different ternary complexes with composition [V^IV^O­(acac)­(H_2_citr)], [V^IV^O­(acac)­(Hcitr)]^−^, and [V^IV^O­(acac)­(citr)]^2–^ (Table 1). The first two species exist with a low concentration in the acidic pH range (2–4), while the latter is the predominant complex between pH 3.5 and 9.0, as indicated by the concentration distribution curves of the formed species (Figure 3).

**Figure 3:**
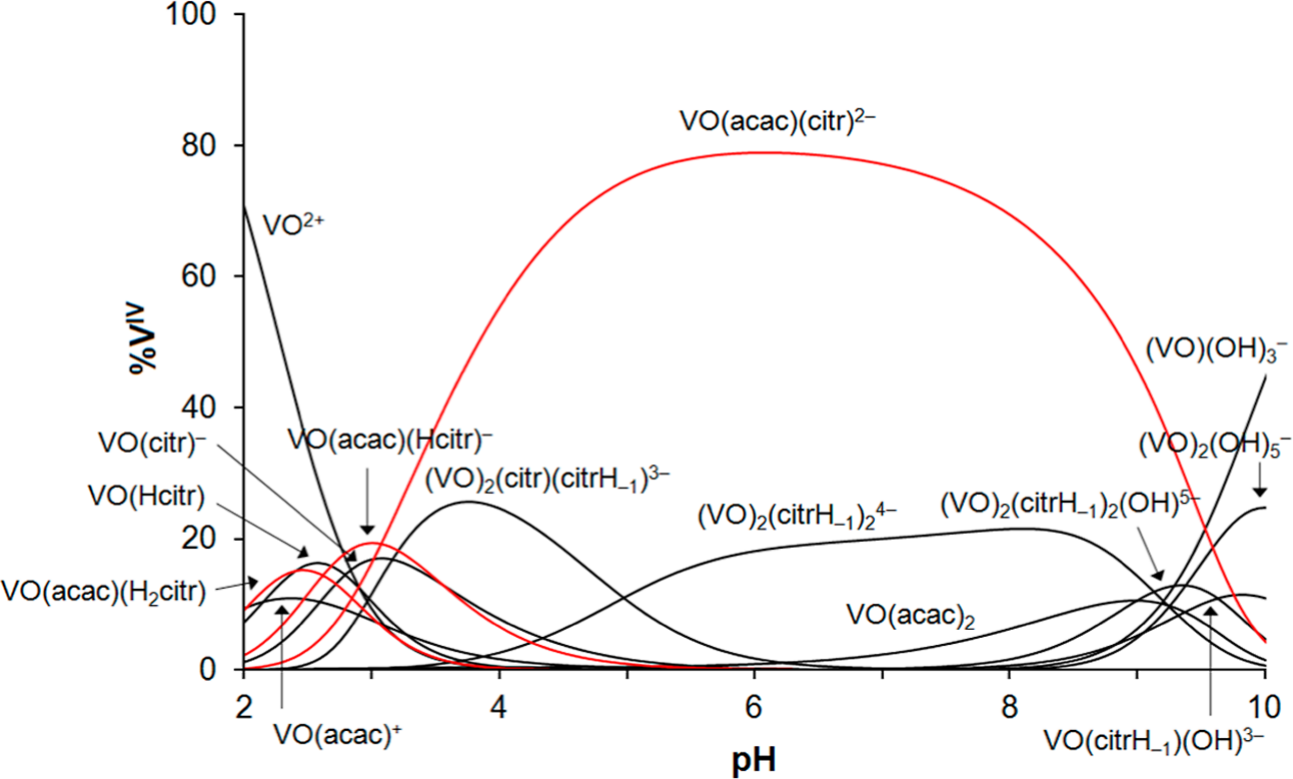
Concentration distribution curves of the species formed as a function of pH in the system V^IV^O^2+^/acac/citr with molar ratio 1:1:1 and *c*_V_ = 2.0 × 10^–3^ M. The curves of the mixed-ligand species are represented in red.

EPR spectra of solutions containing V^IV^O^2+^, acac, and citr with a molar ratio 1:1:1 were recorded at different pH values (*c*_V_ = 2.0 mM). At low pH, the resonances are not well resolved and it is probable that a mixture of species exist in solution, including V^IV^O­(Hcitr)/V^IV^O­(citr)^−^ and V^IV^O­(acac)^+^ plus small amounts of V^IV^O­(acac)­(H_2_citr) and V^IV^O­(acac)­(Hcitr)^−^ (Figure 4). At pH 6.5, the formation of a new species, with |*A*_*z*_| = 171.4 × 10^–4^ cm^–1^, *g*_*z*_ = 1.944, was observed. These parameters suggest that it may be a mixed-ligand species with composition [V^IV^O­(acac)­(citr)]^2–^ (**III** in Figure 4). In fact, |*A*_*z*_|^exptl^ is in good agreement with |*A*_*z*_| estimated with the ‘additivity relationship’ which for an equatorial binding mode [(CO, O^–^)_acac_ + (COO^–^, OH)_citr_] predicts 171.0 × 10^–4^ cm^–1^; moreover, the value of |*A*_*z*_|^exptl^ is similar to that measured for [V^IV^O­(acac)­(lact)], suggesting that the bioligand is deprotonated only at the carboxylic group, while the alcoholic function keeps its proton to give a coordination mode (COO^–^, OH)_citr_; its proposed structure is shown in Scheme 2B. The binding of an equatorial alkoxido-O^–^ would imply a significant decrease in the |*A*_*z*_| value (|*A*_*z*_|^estmtd^ = 160.7 × 10^–4^ cm^–1^), not compatible with |*A*_*z*_|^exptl^. Therefore, in this case, the behavior of citrate differs from that displayed by other systems where the OH group undergoes deprotonation. At higher pH, the major species detected by EPR is [V^IV^O­(acac)_2_] (**V**) that coexists in solution with the dinuclear complex [(V^IV^O)_2_(citrH_–1_)_2_]^4–^ ^58^ (**IV**).

**Figure 4:**
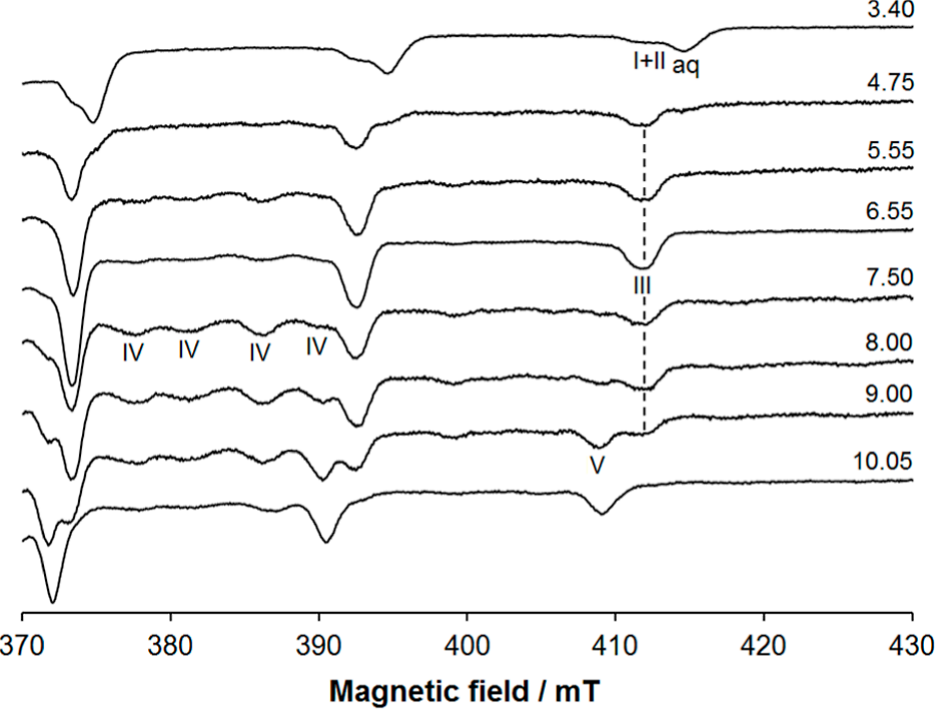
Anisotropic EPR spectra of solutions containing V^IV^O^2+^, acac, and citr with a molar ratio of 1:1:1 and *c*_V_ = 2.0 × 10^–3^ M at several pH values. The resonances of these species are indicated: **aq**, [V^IV^O­(H_2_O)_5_]^2+^; **I** + **II**, [V^IV^O­(Hcitr)]/[V^IV^O­(citr)]^−^ + [V^IV^O­(acac)]^+^; **III**, [V^IV^O­(acac)­(citr)]^2–^; **IV**, [(V^IV^O)_2_(citrH_–1_)_2_]^4–^; and **V**, [V^IV^O­(acac)_2_]. The resonances of the mixed-ligand species [V^IV^O­(acac)­(citr)]^2–^ are also denoted with the dotted line.

ESI-MS spectra of the aqueous solutions were recorded at pH ∼5 and ∼7 with molar ratios of 1:2:2 and 1:1:1 and *c*_V_ = 5.0 × 10^–6^ M. The following ternary species were identified in the positive ion mode spectrum: [V^IV^O­(acac)­(citr)+3H]^+^ (*m*/*z* = 358.0099, Table 2) and [V^IV^O­(acac)­(citr)+2H+Na]^+^ (*m*/*z* = 379.9923, Table 2). In the negative ion mode, [V^IV^O­(acac)­(citr)+H]^−^ (*m*/*z* = 355.9959, Figure S7 of the Supporting Information) and [(V^IV^O)_2_(citr)_2_]^2–^ (*m*/*z* = 255.9432) were revealed. The spectra do not change significantly with the molar ratio and pH.

Visible absorption spectra were recorded with solutions of [V^IV^O­(acac)_2_] adding different amounts of citric acid and adjusting the pH at 5.3 with HCl or NaOH solutions. The significant changes in ε values in the visible absorption spectra (Figure S8 of the Supporting Information) indicate the formation of increasing amounts of a mixed-ligand complex in this pH range, confirming potentiometric, EPR, and ESI-MS results.

### 3.4 Ternary System V^IV^O^2+^/acac/ox

EPR spectra of aqueous solutions containing V^IV^O^2+^, acac, and ox were recorded at different pH values with a molar ratio 1:1:1 (*c*_V_ = 2.0 mM). The major species present in the pH range 3–7 is *cis*-[V^IV^O­(ox)_2_(H_2_O)]^2–^ (**II** in Figure S9 of the Supporting Information).^59^ A small amount of a ternary complex with *g*_*z*_ = 1.948 and |*A*_*z*_| = 162.6 × 10^–4^ cm^–1^ is formed (**III**), with one acac^–^ and an ox^2–^ ligand bound in the equatorial plane of the V^IV^O^2+^ ion (Scheme 2C).

In the ESI-MS(−) spectrum of the system V^IV^O^2+^/acac/ox with molar ratio 1:1:1 at pH ∼5.0 and *c*_V_ = 5.0 × 10^–6^ M, the signal of the mixed-ligand species [V^IV^O­(acac)­(ox)]^−^ (*m*/*z* = 253.9638, Figure S10 of the Supporting Information) was detected (Table 2). The peaks of the binary complex [V^V^O_2_(ox)]^−^ (*m*/*z* = 170.9133) were also identified, indicating the presence of [V^IV^O­(ox)_2_(H_2_O)]^2–^ that undergoes oxidation in solution and/or in-source^60^ during the acquisition of the spectrum. The same process was observed with the ESI-MS technique for other *cis*-octahedral V^IV^O species,^61^ and it is favored by the structure of *cis*-V^IV^O­(H_2_O)^2+^ moiety which yields the *cis*-V^V^O_2_^+^ ion without structural rearrangements.^7^

### 3.5 Ternary System V^IV^O^2+^/acac/His

In the ternary system with [V^IV^O­(acac)_2_] and His, at millimolar concentrations, the mixed-ligand species [V^IV^O­(acac)­(His)] is the major species in aqueous solution between pH 5 and 9 (Figure 5); in particular, it reaches more than 90% of V^IV^ at physiological pH. Its existence suppresses the formation of the bis-chelated species [V^IV^O­(acac)_2_] and [V^IV^O­(His)_2_] in this pH range.

**Figure 5:**
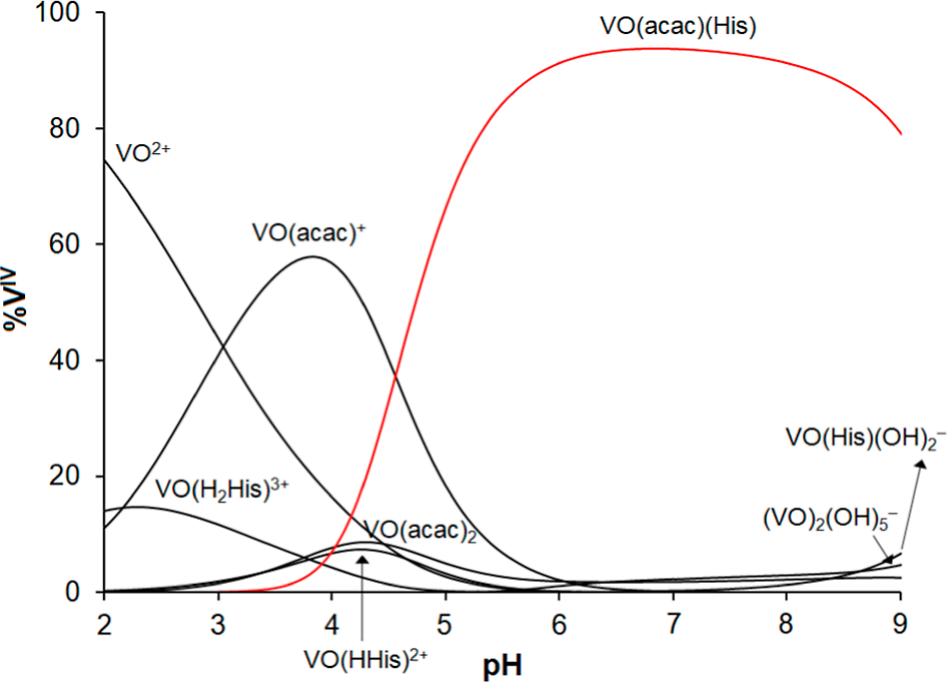
Concentration distribution curves of the species formed as a function of pH in the system V^IV^O^2+^/acac/His with molar ratio 1:1:1 and *c*_V_ = 2.0 × 10^–3^ M. The curve of the mixed-ligand species is represented in red.

The EPR spectra of solutions containing V^IV^O^2+^, acac, and His were recorded at different pH values with a molar ratio 1:1:1 and *c*_V_ = 2.0 mM. After the formation of V^IV^O­(acac)^+^ (**I** in Figure 6) and V^IV^O­(H_*x*_His), with *x* = 1–2,^51c,62^ (**II**) in the acidic pH range, from pH 5 to 9, the signals of the [V^IV^O­(acac)­(His)] mixed-ligand species were observed (**III**, with *g*_*z*_ = 1.954 and |*A*_*z*_| = 162.5 × 10^–4^ cm^–1^). In this complex, acac^–^ is bidentate, while His^–^ is tridentate with one donor in the axial position. The estimated values of |*A*_*z*_|, on the basis of the “additivity relationship”, are 165.2 × 10^–4^ cm^–1^ for the [(CO, O^–^) + (NH_2_, COO^–^, N_im_^ax^)] coordination mode, 165.8 × 10^–4^ cm^–1^ for [(CO, O^–^) + (COO^–^, N_im_, NH_2_^ax^)], and 163.8 × 10^–4^ cm^–1^ for [(CO, O^–^) + (NH_2_, N_im_, COO^–ax^)]. Therefore, the last option appears to be the most plausible coordination mode (Scheme 2D). The formation of the mixed-ligand species is confirmed by the comparison of the spectrum recorded in the ternary system with those recorded, at the same pH, in the binary systems with acac and His (Figure S11 of the Supporting Information).

**Figure 6:**
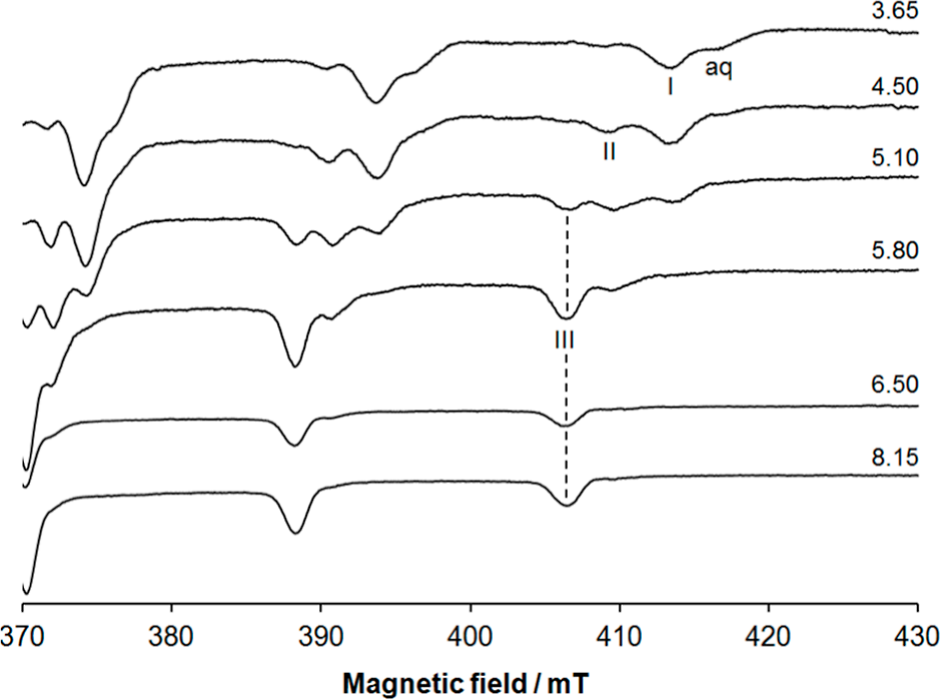
Anisotropic EPR spectra of solutions containing V^IV^O^2+^, acac, and His with a molar ratio of 1:1:1 and *c*_V_ = 2.0 × 10^–3^ M at several pH values. The resonances of these species are indicated: **aq**, [V^IV^O­(H_2_O)_5_]^2+^; **I**, [V^IV^O­(acac)]^+^; **II**, [V^IV^O­(HHis)]^+^: **III**, [V^IV^O­(acac)­(His)]. The resonances of the mixed-ligand species [V^IV^O­(acac)­(His)] are also denoted with the dotted line.

Moreover, EPR spectra were recorded at pH 7 varying the [V^IV^O­(acac)_2_]:His ratio, and they differ from those recorded for the solution only containing [V^IV^O­(acac)_2_] (Figure S12 of the Supporting Information).

ESI-MS­(+) spectra of the system V^IV^O^2+^/acac/His with molar ratios of 1:2:2 and 1:1:1 were recorded at pH ∼7 (*c*_V_ = 5.0 × 10^–6^ M) and are represented in Figure 7. The peaks of the mixed-ligand complexes [V^IV^O­(acac)­(His)+H]^+^ (*m*/*z* = 321.0527, see Figure S13 of the Supporting Information for the simulation of the isotopic pattern), [V^IV^O­(acac)­(His)+Na]^+^ (*m*/*z* = 343.0346) and [V^IV^O­(acac)­(His)+K]^+^ (*m*/*z* = 359.0091) were identified (Table 2). The spectra do not significantly change with the molar ratio. The signals of the bis-chelated complexes with histidine were not observed in the mass spectra.

**Figure 7:**
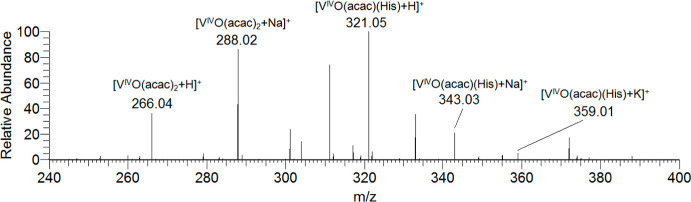
ESI-MS­(+) spectrum recorded on the system V^IV^O^2+^/acac/His 1:1:1 (ultrapure LC–MS water, pH = 7.45 and *c*_V_ = 5.0 × 10^–6^ M). Only two decimal numbers are reported for clarity.

Several visible absorption and CD spectra were recorded with solutions containing [V^IV^O­(acac)_2_] alone (Figures S14 of the Supporting Information), as well as [V^IV^O­(acac)_2_] and His (Figures 8 and S15–S21 of the Supporting Information), at several pH values and [V^IV^O­(acac)_2_]:His molar ratios. Figure 8 depicts the spectra measured with a [V^IV^O­(acac)_2_]:His molar ratio of 1:2 at several pH values. Note that CD bands in the visible range of the spectra can only be seen if a chiral ligand, such as l-histidine, binds to V^IV^O.^51c,62^ At pH 4.0, the addition of His to the solution containing [V^IV^O­(acac)_2_] gives rise to quite weak CD bands, and only for higher molar ratios, the bands become more easily visible (Figure S15B of the Supporting Information). When the pH is increased to 7 and 8, the CD spectra remain similar, but at these pH values, the use of 2 equiv of His is enough to yield important changes and relatively high |Δε| values (Figure 8), this corresponding to the formation of increasing concentrations of mixed V^IV^O–acac–His species. The CD data can be interpreted postulating the formation of mixed-ligand species that start at acidic pH values, and significantly increase their concentration at physiological pH, in agreement with EPR data. The visible and CD spectra recorded at fixed pH with varying the [V^IV^O­(acac)_2_]:His molar ratios and at a fixed ratio changing the pH, shown in Figures S15–S21 of the Supporting Information, globally agree with the data of the species diagram depicted in Figure 5.

**Figure 8:**
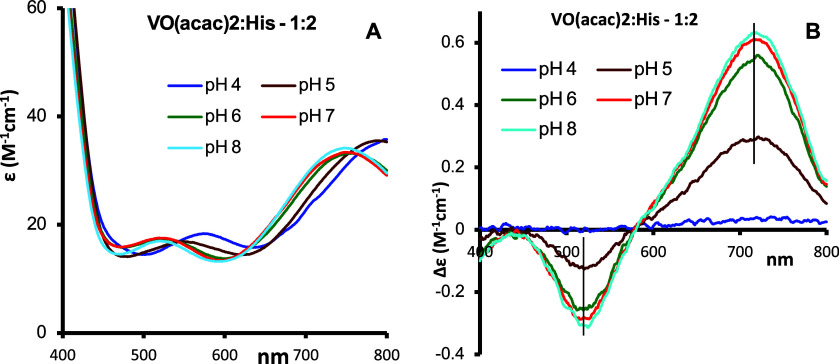
Spectra of solutions containing [V^IV^O­(acac)_2_] and His with a [V^IV^O­(acac)_2_]:His molar ratio of 1:2 at several pH values. The *c*_V_ is ∼2.1 mM, and the spectra correspond to those represented in Figures S15–S19 of the Supporting Information. (A) Visible absorption spectra and (B) CD spectra, where the approximate positions of the λ_max_ of bands are indicated (for the negative band, the λ_max_ slightly decreases with increase in pH, while the opposite happens with the positive band).

### 3.6 Ternary System V^IV^O^2+^/acac/Cys

EPR spectra of the system V^IV^O^2+^/acac/Cys were recorded at different pH values with a molar ratio 1:1:1 (*c*_V_ = 2 mM). The major species observed in solution in the pH range 6.0–8.5 was [V^IV^O­(acac)_2_] (**II** in Figure S22 of the Supporting Information), together with [V^IV^O­(Cys)_2_]^2–^ (**IV**). This latter species exhibits the same spin Hamiltonian parameters (*g*_*z*_ = 1.958, |*A*_*z*_| = 154 × 10^–4^ cm^–1^) as the bis-chelated complex of 3-mercaptopropionic acid with 2 × (COO^–^, S^–^) coordination.^63^ According to Costa Pessoa et al., in this species one of the two amino groups is likely bound in the apical position of the metal ion.^64^

In the pH range 7.0–9.0, the mixed-ligand complex [V^IV^O­(acac)­(Cys)]^−^, with EPR parameters intermediate between [V^IV^O­(acac)_2_] and [V^IV^O­(Cys)_2_]^2–^, is formed in a small amount (**III** in Figure S22 of the Supporting Information). This is confirmed by the spectra recorded as a function of the ratio [V^IV^O­(acac)_2_]:Cys that are different from that of [V^IV^O­(acac)_2_] alone at pH 7, but not at pH 5 (Figures S23 and S24 of the Supporting Information). In principle, three different arrangements of Cys are possible, but it is plausible that the binding mode is identical to that in [V^IV^O­(Cys)_2_]^2–^, i.e., (COO^–^, S^–^, NH_2_^ax^) (Scheme 3A).^64^

**Scheme 3:**
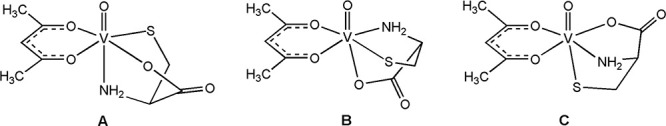
Proposed Structure of the Mixed-Ligand Species [V^IV^O­(acac)­(Cys)]^−^ Formed in the Ternary System V^IV^O^2+^/acac/Cys with Different Coordination Mode of Cys Ligand: (A) Coordination (COO^–^, S^–^, NH_2_^ax^); (B) Coordination (NH_2_, S^–^, COO^–ax^); and (C) Coordination (NH_2_, COO^–^, S^–ax^); According to the EPR Spectroscopy, Only Species **A** Is Observed in Aqueous Solution with a Minor Amount

ESI-MS spectra of the system V^IV^O^2+^/acac/Cys with a molar ratio 1:2:2 and 1:1:1 were recorded at pH 4.8 and 7.5 (*c*_V_ = 5.0 × 10^–6^ M) and the following species were identified (Table 2): [V^IV^O­(acac)­(Cys)+2H]^+^ (*m*/*z* = 287.0027) and [V^IV^O­(acac)­(Cys) + H + Na]^+^ (*m*/*z* = 308.9847, experimental and calculated isotopic pattern in Figure S25 of the Supporting Information).

The addition of Cys to a solution of [V^IV^O­(acac)_2_] at pH ∼4.0 (Figure S26 of the Supporting Information) up to a [V^IV^O­(acac)_2_]:Cys molar ratio 1:4 has some effect on the visible absorption spectra, but not on CD spectra, where no bands are recorded; this means that, at this pH, there is no significant formation of species having Cys bound to V^IV^O^2+^. Only when the pH value reaches ca. 5.0 (Figure S27 of the Supporting Information), and particularly 6.0 (Figure S28 of the Supporting Information), CD bands of low intensity were observed, indicating a significant increase in the presence of chiral V^IV^O complexes. At pH 6.0, the intensity of bands is still low. Noteworthy, the CD spectra all cross very close to Δε = 0 at ∼492 nm; this finding and the constant ratio of Δε values of the bands at ∼570 and 750 nm suggest that, with the increase in the molar ratio, there is a transformation of nonchiral species to a single chiral complex.

From pH 6.0 to 8.0, the intensity of the CD bands continue increasing and a change in the pattern of bands is observed, suggesting there is a modification in the type of chiral V^IV^O complexes present (Figures S29 and S30 of the Supporting Information); notably, the pattern is maintained from pH 7.0 to 8.0 and this means that the binding mode remains the same. This species should be [V^IV^O­(acac)­(Cys)]^−^, according to the EPR and ESI-MS data. Finally, at pH 9.5, it is also possible to observe quite significant changes in the visible absorption and CD spectra as Cys is added to [V^IV^O­(acac)_2_], indicating that a distinct chiral V^IV^O complex is formed (Figure S31 of the Supporting Information). In particular, λ_max_ in the CD bands change with the [V^IV^O­(acac)_2_]:Cys molar ratio: it is ∼730 nm, ∼715 nm, and ∼708 nm for 1:1, 1:2 and 1:4 ratios, respectively. On the contrary, the intensity of the CD band with λ_max_ ∼655 nm remains constant for ratios 1:2 and 1:4. This is a clear indication of the formation of a distinct chiral species, where one acac^–^ is substituted by a Cys ligand; it should be [V^IV^O­(Cys)_2_]^2–^, according to EPR measurements (Figure S25 of the Supporting Information).

Therefore, globally the visible absorption and CD spectra, including those of Figures S32–S34 of the Supporting Information, indicate a significant increase of the presence of chiral V^IV^O complexes as the pH and/or amount of Cys is increased with the formation of [V^IV^O­(acac)­(Cys)]^−^ and [V^IV^O­(Cys)_2_]^2–^, revealed also with EPR spectroscopy. The minor species present in the pH range 5.0–6.0 that gives rise to a somewhat different pattern of CD bands (Figures S27 and S28 of the Supporting Information) may correspond to either [V^IV^O­(Cys)­(HCys)]^− 64^ or [V^IV^O­(acac)­(HCys)] that may form in low amounts and are not detected by EPR. Some changes are also seen in the visible absorption spectra upon addition of Cys. Part of these modifications may correspond to a decrease in the relative amounts of hydrolytic V^IV^O species as Cys is added.

### 3.7 Ternary System V^IV^O^2+^/acac/Asp

The data of pH-potentiometry indicate that, when aspartic acid is present in the ternary system with V^IV^O^2+^ and acac, two mixed-ligand species are formed: [V^IV^O­(acac)­(HAsp)] from pH 3 to 5 reaching ca. 15% of total V in solution and [V^IV^O­(acac)­(Asp)]^−^ in the pH range 4–10 (Figure 9).

**Figure 9:**
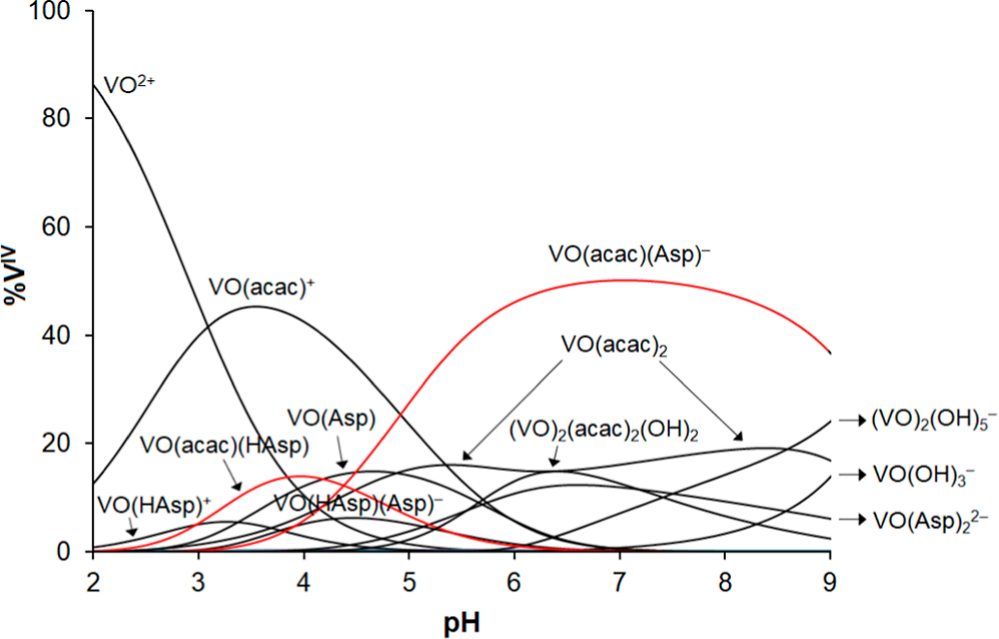
Concentration distribution curves of the species formed as a function of pH in the system V^IV^O^2+^/acac/Asp with molar ratio 1:1:1 and *c*_V_ = 2.0 × 10^–3^ M. The curves of the mixed-ligand species are represented in red.

EPR spectra of the system V^IV^O^2+^/acac/Asp suggest that, in the pH range 4.5–6.0, [V^IV^O­(acac)_2_] (**III** in Figure S35 of the Supporting Information) is formed together with another species (**II**), with EPR parameters, *g*_*z*_ = 1.944, |*A*_*z*_| = 173.3 × 10^–4^ cm^–1^, similar to those measured in the system V^IV^O^2+^/acac/lact. This result, also considered in relation to the potentiometric data, allow us to confirm that this is the mixed-ligand complex with composition [V^IV^O­(acac)­(HAsp)], where aspartate—still protonated at the amino group—binds V^IV^ with a (COO^–^, COO^–^) donor set. For pH >5, after the deprotonation of the –NH_3_^+^, [V^IV^O­(acac)­(HAsp)] transforms into [V^IV^O­(acac)­(Asp)]^−^, which exists under two isomers, one with (COO^–^, COO^–^, NH_2_^ax^) binding mode (**II**) and another with (NH_2_, COO^–^, COO^–ax^) coordination (**IV**), this latter isomer being detected over the low-field resonances of [V^IV^O­(acac)_2_] (**III**). The approximate EPR parameters of **IV** are *g_z_* ca. 1.947 and |*A_z_* | ca. 166 × 10^–4^ cm^–1^, with |*A_z_* |^exptl^ very close to |*A_z_* | estimated with the “additivity relationship” (165.6 × 10^–4^ cm^–1^).

ESI-MS spectra of the system V^IV^O^2+^/acac/Asp with molar ratios 1:2:2 and 1:1:1 were recorded at pH 4.85 with *c*_V_ = 5.0 × 10^–6^ M (Figure S36 of the Supporting Information) and the following mixed-ligand species were identified: [V^IV^O­(acac)­(Asp)+2H]^+^ (*m*/*z* = 299.0209, the experimental and calculated isotopic pattern are shown in Figure S37 of the Supporting Information) and [V^IV^O­(acac)­(Asp)+H+Na]^+^ (*m*/*z* = 321.0026) in the positive mode (see also Table 2). Notably, the intensity of the peaks in the mass spectra of the mixed-ligand complex with Asp is intermediate between those of the ternary species with His and Cys; therefore, even if the ESI-MS technique is not appropriate to quantitatively support the speciation details, it must be observed that this follows the order of percent amount in aqueous solution.

The visible absorption and CD spectra of the binary system V^IV^O^2+^/Asp were reported in the literature.^51a^ The addition of Asp at pH ∼4 has a small effect in the visible absorption spectra and more pronounced effect in CD. A larger excess of Asp (ratio [V^IV^O­(acac)_2_]:Asp 1:4) ensures already an appreciable CD band intensity (Figure S38 of the Supporting Information). At pH 5 and 6, the changes in both visible absorption and CD spectra are more significant (Figures 10 and S39 of the Supporting Information), changes that are similar when the pH was increased up to 7 and 8 (Figures S40 and S41 of the Supporting Information). In Figures S42–S44 of the Supporting Information, the spectra are presented as a function of pH, keeping the ratio [V^IV^O­(acac)_2_]:Asp constant.

**Figure 10:**
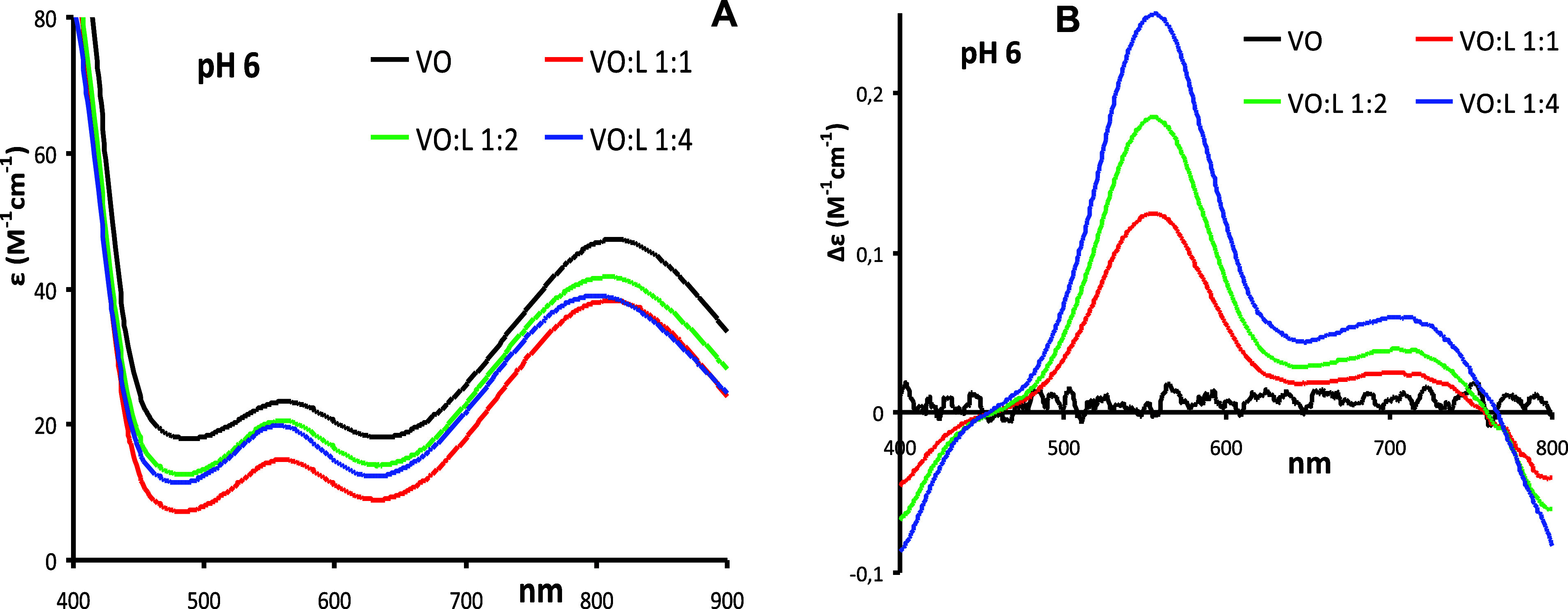
Spectra of solutions containing [V^IV^O­(acac)_2_] upon additions of Asp at pH ∼6.00 and *c*_V_ ∼2.0 mM. The VO/L ratios indicated correspond to [V^IV^O­(acac)_2_]:Asp molar ratios. (A) Visible absorption spectra in the range 450–900 nm and (B) circular dichroism spectra in the range 400–800 nm. Note that the black CD spectrum was recorded with a solution of [V^IV^O­(acac)_2_], where no CD bands are expected to exist.

The pattern of CD spectra is similar in all cases. They show two negative bands with λ_max_ < 400 nm and λ_max_ > 790 nm, and two positive bands at 540–560 and 680–730 nm whose intensity increases with pH and [V^IV^O­(acac)_2_]:Asp molar ratios; both positive CD bands also shift their λ_max_, this being more relevant for those at ca. 680–730 nm.

The CD spectra measured in the pH range 4–5 have contributions from [V^IV^O­(HAsp)]^+^, [V^IV^O­(Asp)], and [V^IV^O­(HAsp)_2_],^51a^ but globally differ from those measured under the same conditions for the binary V^IV^O^2+^/Asp system. Therefore, the distinct pattern obtained and the changes with pH and [V^IV^O­(acac)_2_]:Asp molar ratios can only be explained assuming the formation of [V^IV^O­(acac)­(HAsp)], and the progressive formation of [V^IV^O­(acac)­(Asp)]^−^ as pH is increased. We may conclude that the recorded CD spectra, depicted in Figures 10 and S38–S44 of the Supporting Information, are consistent and support the findings made from the potentiometric data, as well as EPR and ESI-MS techniques.

### 3.8 Ternary System V^IV^O^2+^/acac/P_2_O_7_^4–^

The pyrophosphate anion is involved in numerous biosynthetic processes and is an appropriate ligand to model the binding to V^IV^O^2+^ of the diphosphate or triphosphate moiety of ADP and ATP. Three different mixed-ligand species can be identified by pH-potentiometry in the ternary system, V^IV^O^2+^/acac/P_2_O_7_^4–^: [V^IV^O­(acac)­(H_2_P_2_O_7_)]^−^, [V^IV^O­(acac)­(HP_2_O_7_)]^2–^, and [V^IV^O­(acac)­(P_2_O_7_)]^3–^, the latter two being the predominant ones in the pH range 2.5–5.5 and 5.5–10.5, respectively (Figure 11). Plausible structures are depicted in Scheme 4A,B.

**Figure 11:**
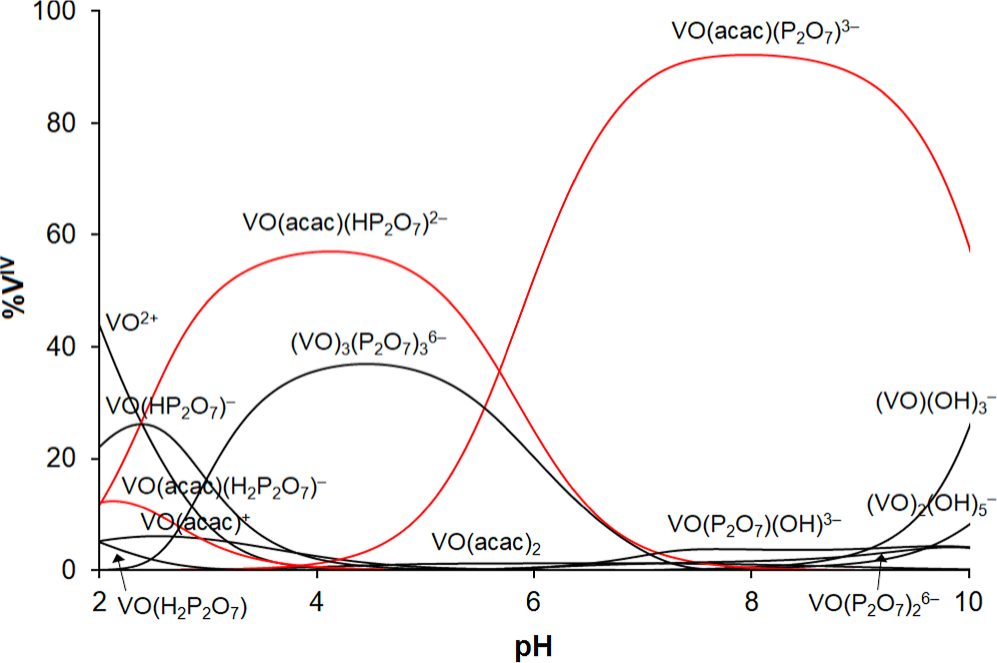
Concentration distribution curves of the species formed as a function of pH in the system V^IV^O^2+^/acac/P_2_O_7_^4–^ with molar ratio 1:1:1 and *c*_V_ = 2.0 × 10^–3^ M. The curves of the mixed-ligand species are represented in red.

**Scheme 4:**
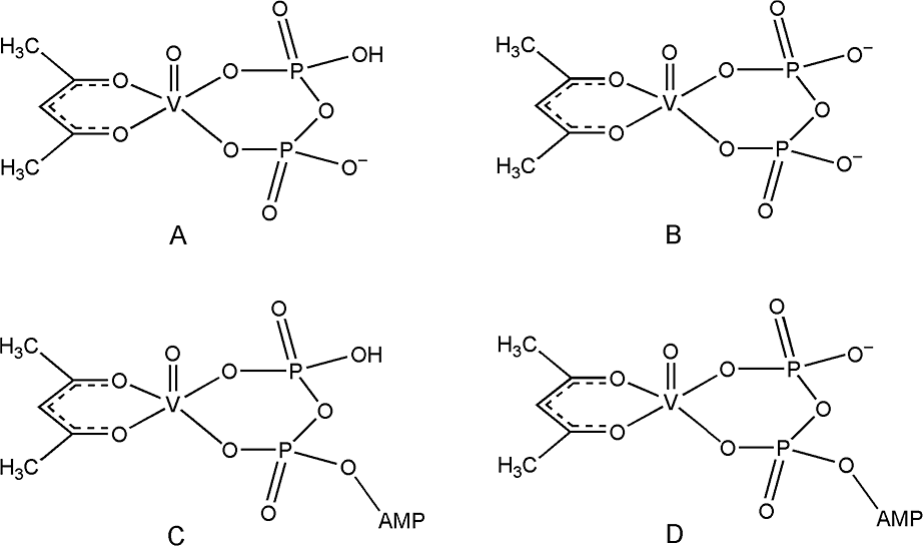
Plausible Structures of the Mixed-Ligand Species Formed in the Ternary System with Pyrophosphate and ATP: (A) [V^IV^O­(acac)­(HP_2_O_7_)]^2–^; (B) [V^IV^O­(acac)­(P_2_O_7_)]^3–^; (C) [V^IV^O­(acac)­(HATP)]^2–^; and (D) [V^IV^O­(acac)­(ATP)]^3–^

EPR spectra of the solutions containing V^IV^O^2+^, acac, and P_2_O_7_^4–^ anion, recorded with a molar ratio 1:1:1 (*c*_V_ = 2.0 mM), confirm the results of the pH-potentiometric titrations (Figure S45 of the Supporting Information). A first mixed-ligand complex is observed between pH 4.0 and 5.5 with *g*_*z*_ = 1.941 and |*A*_*z*_| = 173.2 × 10^–4^ cm^–1^ (**I**). From pH 5 to 9, the signals of the mixed-ligand species [V^IV^O­(acac)­(P_2_O_7_)]^3–^ were also detected (**II**). Its spin Hamiltonian parameters are *g*_*z*_ = 1.943 and |*A*_*z*_| = 171.6 × 10^–4^ cm^–1^. In these complexes, acac^–^ is bidentate, while P_2_O_7_^4–^ forms a six-membered chelate ring. It must be added that, in the range pH 4–6, the trinuclear species [(V^IV^O)_3_(P_2_O_7_)_3_]^6–^ is revealed in the EPR spectra by the series of absorptions indicated by **III** with a low |*A*_*z*_| and by the broad central band, due to the exchange and dipolar interaction of the neighboring paramagnetic V^IV^O^2+^ ions;^65^ this is in agreement with concentration distribution curves (Figure 11) and previous results in the literature.^51e^

The mass spectra in the negative-ion mode of the system V^IV^O^2+^/acac/P_2_O_7_^4–^ 1:1:5 were recorded at pH 9.0 and the peak related to the mixed-ligand species ([V^IV^O­(acac)­(P_2_O_7_)+2H]^−^) was observed at *m*/*z* = 341.9119 (the simulation of the isotopic pattern is shown in Figure S46 of the Supporting Information), confirming the results obtained with pH-potentiometry and EPR spectroscopy.

### 3.9 Ternary System V^IV^O^2+^/acac/ATP

The ternary complexes identified by pH-potentiometry in the ternary system V^IV^O^2+^/acac/ATP are [V^IV^O­(acac)­(HATP)]^2–^ and [V^IV^O­(acac)­(ATP)]^3–^, being predominant in the range 3.0–7.5 (Figure 12). The coordination of ATP in these species takes place with phosphate oxygen donors (Scheme 4C,D).

**Figure 12:**
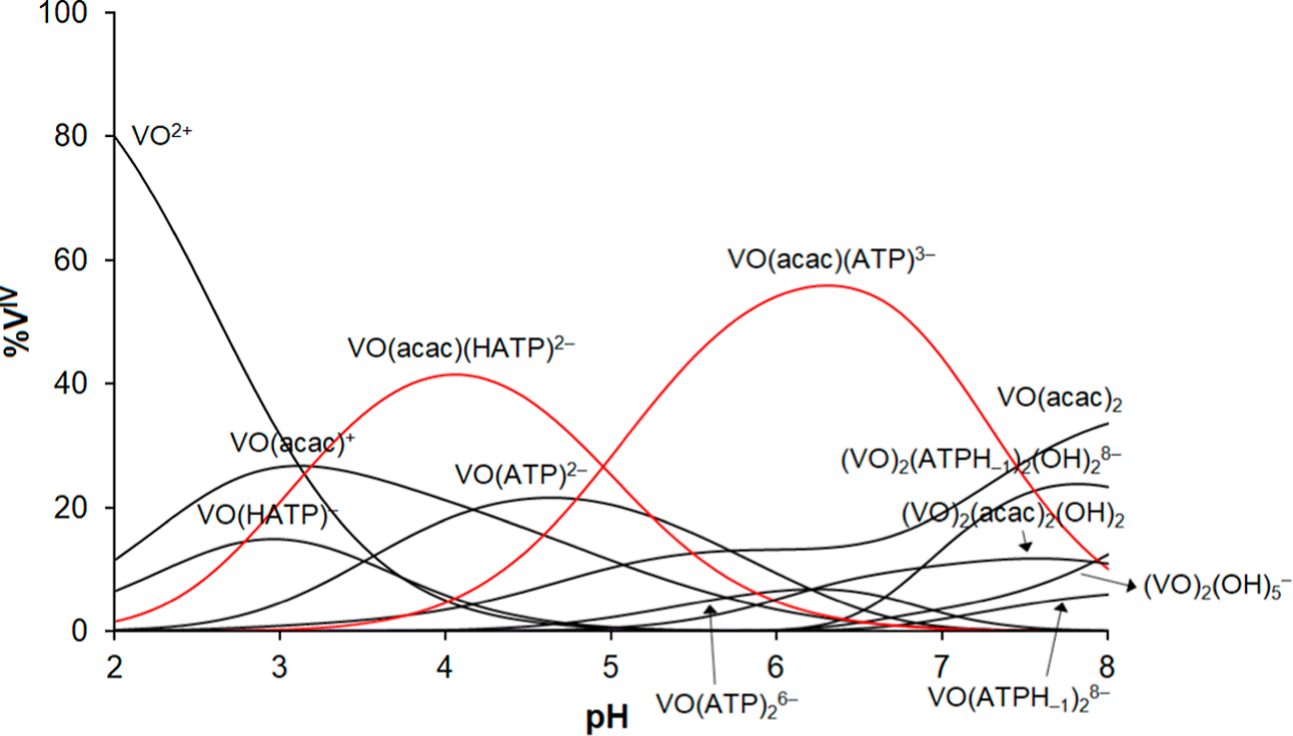
Concentration distribution curves of the species formed as a function of pH in the system V^IV^O^2+^/acac/ATP with molar ratio 1:1:1 and *c*_V_ = 2.0 × 10^–3^ M. The curves of the mixed-ligand species are represented in red.

EPR spectra were recorded at different pH values with a molar ratio 1:1:5 (*c*_V_ = 2.0 mM). In fact, relatively high ATP:V^IV^O^2+^ ratios were necessary to observe a clear EPR signal compared with the previously analyzed ligands. In the pH range 3–6, three species were observed: [V^IV^O­(ATP)]^2–^ (**I** in Figure 13) with *g*_*z*_ = 1.930, |*A*_*z*_| = 181.0 × 10^–4^ cm^–1^ and [V^IV^O­(ATP)_2_]^6–^ (**II**) with *g*_*z*_ = 1.934, |*A*_*z*_| = 178.2 × 10^–4^ cm^–1^, where ATP binds V^IV^O with the phosphate groups forming a six-membered chelate ring. The resonances of [V^IV^O­(acac)­(HATP)]^2–^ appear as a shoulder of the signals of the previous species (**III**). Above pH 6, a species with spin Hamiltonian parameters similar to **III**, *g*_*z*_ = 1.942, |*A*_*z*_| = 172.7 × 10^–4^ cm^–1^, is detected. It is indicated with **IV** in Figure 13 and can be explained with the deprotonation of the diphosphate moiety, without changing the donor set of the ligand. At pH > 9, two other species were revealed in small amounts (**V** and **VI**), which should correspond to the bis-chelated complexes of ATP and 2 × (OH, O^–^) and 2 × (O^–^, O^–^) bindings of the sugar ring, their composition being [V^IV^O­(ATPH_–1_)_2_]^8–^ (**V**) and [V^IV^O­(ATPH_–2_)_2_]^10–^ (**VI**); these species are relevant at very basic pH values.^51d^

**Figure 13:**
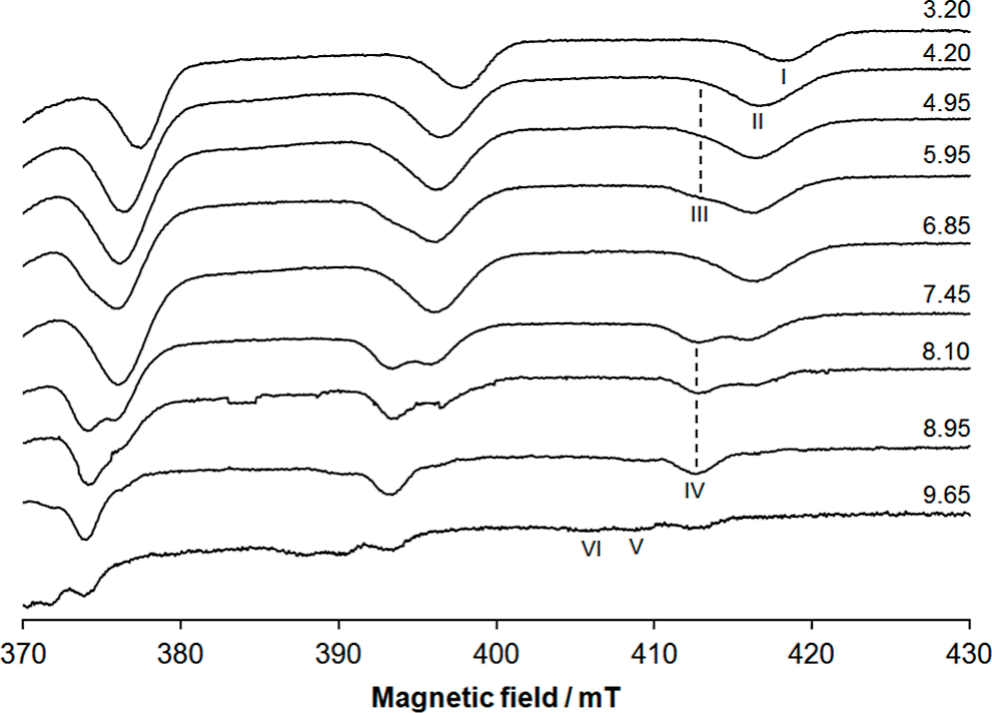
Anisotropic EPR spectra of solutions containing V^IV^O^2+^, acac, and ATP with a molar ratio of 1:1:5 and *c*_V_ = 2.0 × 10^–3^ M at several pH values. **I**, [V^IV^O­(ATP)]^2–^; **II**, [V^IV^O­(ATP)_2_]^6–^; **III**, [V^IV^O­(acac)­(HATP)]^2–^; **IV**, [V^IV^O­(acac)­(ATP)]^3–^; **V**, [V^IV^O­(ATPH_–2_)_2_]^8–^; and **VI**, [V^IV^O­(ATPH_–4_)_2_]^10–^. The resonances of the mixed species [V^IV^O­(acac)­(HATP)]^2–^/[V^IV^O­(acac)­(ATP)]^3–^ are also denoted with dotted lines.

It must be observed that potentiometry does not provide useful support to the EPR (and CD, see below) findings above pH ∼ 8–9, because a significant rearrangement of the binding mode at the metal center occurs parallel with progressive hydrolysis, which causes very slow processes.

ESI-MS­(−) spectra were recorded at pH 8.5 and V^IV^O^2+^ concentration 5.0 × 10^–6^ M, using a molar ratio V^IV^O^2+^:acac:ATP 1:1:10. The following species were identified: [V^IV^O­(acac)­(ATP)+H]^2–^ (*m*/*z* = 334.9789), [V^IV^O­(ATP)]^2–^ (*m*/*z* = 284.9525), and [V^IV^O­(ATP)+H]^−^ (*m*/*z* = 570.9126). The simulation of the isotopic pattern of [V^IV^O­(acac)­(ATP)+H]^2–^ is shown in Figure S47 of the Supporting Information.

Some relatively small changes are observed in the visible absorption and CD spectra in the pH range 4.0–8.5, while for pH > 8.5, both types of spectra depict significant modifications, more evident from pH 9.5 to 11.0 (Figure 14), where the most relevant chiral species is [V^IV^O­(ATPH_–2_)_2_]^10–^. The modification of the pattern of the visible absorption and CD spectra in the range ca. 8.5–9.5 discloses a clear change in the coordination environment of V^IV^O center, with the progressive binding of deprotonated hydroxyl groups of the ribose fragment. Besides the change in the CD spectral pattern, the intensity of the induced CD bands increases significantly at pH 9.8 and 11.0, because the donor atoms involved are now close to the stereogenic centers of the ATP molecule.

**Figure 14:**
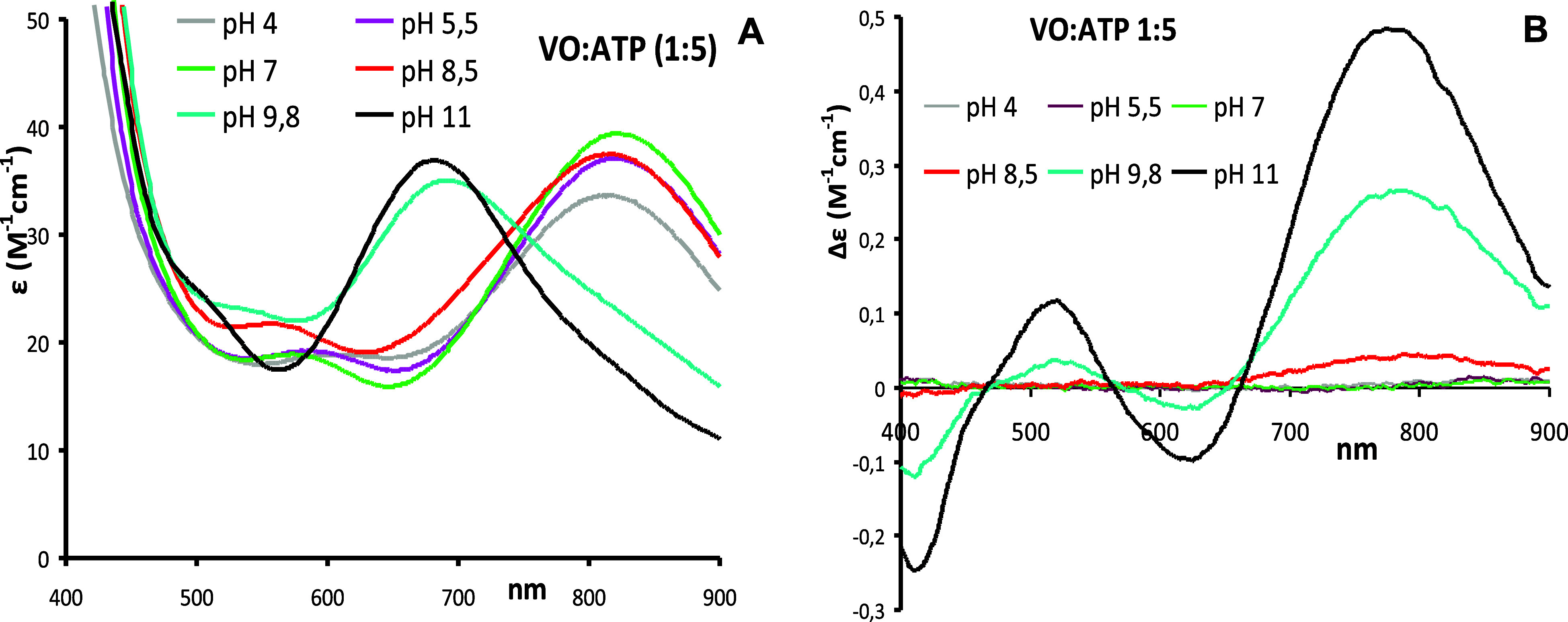
Spectra of solutions containing [V^IV^O­(acac)_2_] and ATP with a 1:5 molar ratio and *c*_V_ ∼2.0 mM at several pH values. (A) Absorption spectra in the range 400–900 nm and (B) circular dichroism spectra in the range 400–900 nm.

### 3.10 Ternary System V^IV^O^2+^/acac/GSH

EPR spectra of the system V^IV^O^2+^/acac/GSH with a molar ratio 1:1:1 were recorded at different pH values and *c*_V_ ∼2.0 mM. A ternary complex was revealed around pH 4 with a composition of [V^IV^O­(acac)­(HGSH)]^−^ and binding mode [(CO, O^–^) + (NH_2_, COO^–^)], probably with the –SH still protonated (**II** in Figure S48 of the Supporting Information). Its spin Hamiltonian parameters are *g*_*z*_ = 1.943 and |*A*_*z*_| = 172.4 × 10^–4^ cm^–1^. A second mixed-ligand compound appears in a small amount around pH 9.5 after the deprotonation of the thiol and amide groups; to this species, the composition [V^IV^O­(acac)­(GSHH_–1_)]^3–^ and the coordination mode [(CO, O^–^) + (N_amide_^–^, S^–^)] are assigned (**VI** in Figure S48 of the Supporting Information); its approximate spin Hamiltonian parameters are *g*_*z*_ ∼1.954 and |*A*_*z*_| ∼158 × 10^–4^ cm^–1^.

The mass spectrum of a solution containing V^IV^O^2+^, acac, and GSH was recorded at pH 4.80 with a molar ratio of 1:1:25 and *c*_V_ = 5.0 × 10^–6^ M. Similarly to the system with ATP and in line with what was reported in the literature,^66^ the binding of GSH to V^IV^O^2+^ requires a large excess of GSH; so the mass spectra, as well as CD and visible absorption spectra were measured under such experimental conditions. The following mixed-ligand species were identified: [V^IV^O­(acac)­(GSH)+3H]^+^ (*m*/*z* = 473.0669) and [V^IV^O­(acac)­(GSH) + 2H + Na]^+^ (*m*/*z* = 495.0489 with a very low intensity). The experimental and calculated isotopic patterns for [V^IV^O­(acac)­(GSH)+3H]^+^ were compared in Figure S49 of the Supporting Information. The stoichiometry of the mixed-ligand species was confirmed with the fragmentation pattern, and the MS/MS spectrum is shown in Figure 15. The peaks at *m*/*z* = 373.0147 and *m*/*z* = 391.0252, derived from the fragmentation process, correspond to [V^IV^O­(GSH)+2H]^+^ and [V^IV^O­(GSH)­(H_2_O)+2H]^+^.

**Figure 15:**
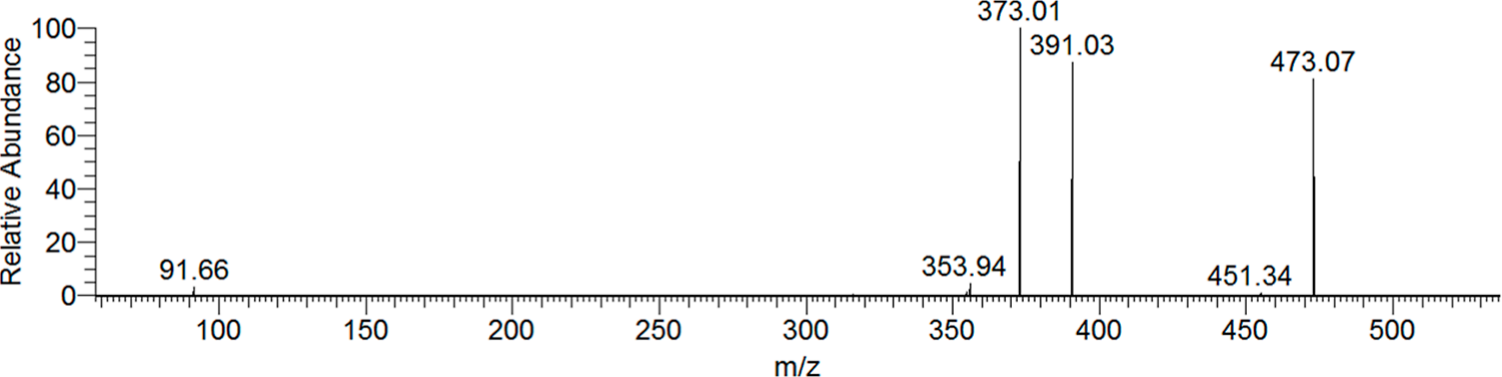
ESI-MS/MS spectrum of the species [V^IV^O­(acac)­(GSH)+3H]^+^ recorded in the positive ion mode in a *m*/*z* range of 473.0 ± 1 on the system with a V^IV^O^2+^:acac:GSH ratio of 1:1:25 (ultrapure LC–MS water, pH = 5.70, *c*_V_ = 5.0 × 10^–6^ M). Only two decimal numbers are reported for clarity.

The visible absorption spectra, recorded with [V^IV^O­(acac)_2_]:GSH molar ratios of either 1:5 or of 1:12.5, differ from those obtained under similar experimental conditions in the binary V^IV^O^2+^/GSH system (Figure 16).^66^ As pH changes from 4 to 7, some relevant modifications were observed. First, the ε values at ca. 810 nm increase up to pH = 7, and the addition of more GSH causes a decrease of ε. Moreover, the addition of a base yields a further decrease in the value of ε, particularly for pH 9.8 and 11. The band at ca. 575 nm is much less affected. Also, a change in the solution’s coloration (from greenish blue to yellow) indicates a change in the species present therein. Noteworthy, the visible spectra up to pH ∼9.5 do not suggest the extensive hydrolysis of the V^IV^O complexes formed, as occurs in the absence of acetylacetone,^66^ this further suggesting that an acac^–^ ligand is bound to V^IV^O^2+^ and that mixed-ligand species are present in solution at least until pH 10.

**Figure 16:**
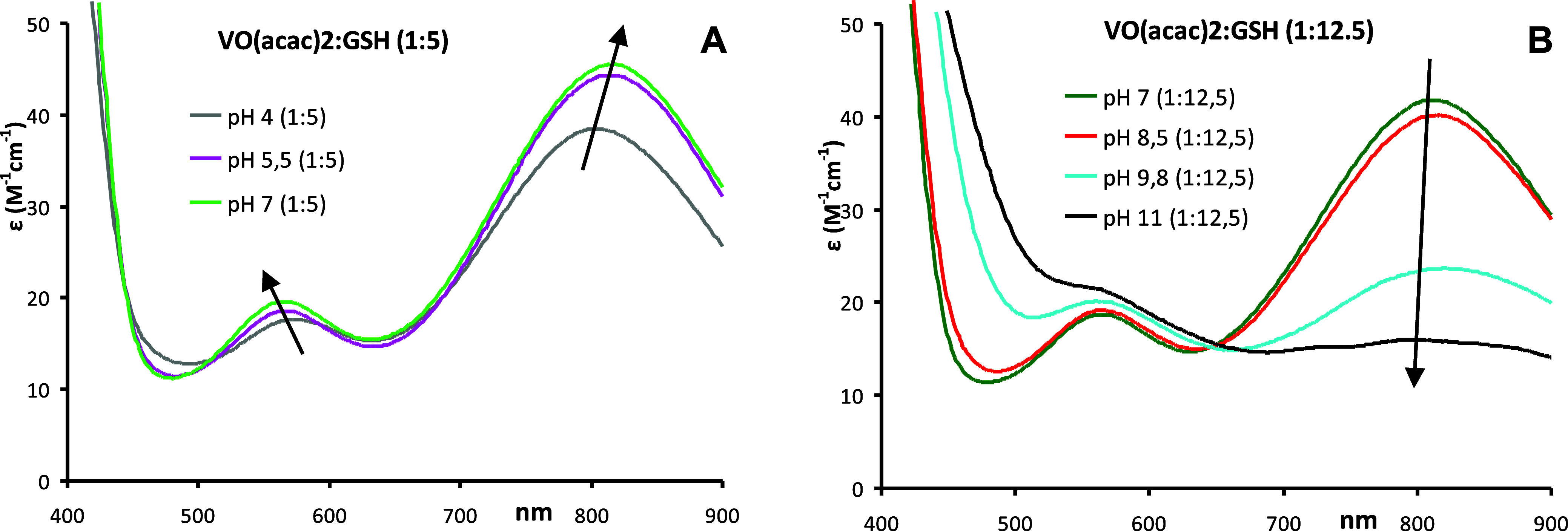
Absorption spectra of solutions containing [V^IV^O­(acac)_2_] and GSH with molar ratios of 1:5 and 1:12.5 and *c*_V_ ∼2.0 mM at several pH values: (A) ratio 1:5, from pH 4 to 7 and (B) ratio 1:12.5, from pH 7 to 11.

The CD spectra measured for this system using identical molar ratios (Figure S50 of the Supporting Information) are very weak in intensity and the signal-to-noise ratio is low. In the pH range 4–7, the Δε values are close to zero or very small, even if it should be noted that at pH = 5 there is a barely noticeable negative Δε band in the range 500–600 nm. The λ_max_ of the bands differ from those obtained under similar experimental conditions in the V^IV^O^2+^/GSH system,^66^ indicating that a distinct species forms in solution, with acac^–^ and GSH bound to V^IV^; this confirms the results obtained with EPR and ESI-MS techniques, which suggested the formation of the [V^IV^O­(acac)­(HGSH)]^−^ mixed-ligand complex in this pH range.

At pH = 7, comparing the data with the molar ratios [V^IV^O­(acac)_2_]:GSH 1:5 and 12.5, the Δε values at ca. 650–800 nm increase, but the weak band at ca. 500–600 nm is not much affected. The maximum Δε values are observed at pH = 8.5, while at pH = 11 Δε is close to zero. The fact that only weak intensity CD spectra were recorded in this system may indicate that the amount of the ternary species is low when the ratio [V^IV^O­(acac)_2_]:GSH is 1:5–1:12.5 (it must be considered that EPR and ESI-MS measurements were carried out using a 1:25 ratio) and/or that the binding of vanadium to GSH, under these conditions, is established at sites far from the molecule’s stereogenic centers.

### 3.11 Speciation of [V^IV^O­(acac)_2_] in the Blood Serum

After a vanadium-based drug is administered by intravenous/intraperitoneal injections,^18b,67^ it enters the bloodstream. This route ensures that the administered compound—[V^IV^O­(acac)_2_] in the present work—reaches the bloodstream in its original form and allows a direct investigation of its interactions with serum components and biologically relevant ligands. Until now, it was not clear the fraction which remains in the serum and the part taken up by the erythrocytes. In the literature, it is reported that ca. 90% of V of blood is associated with the serum fraction, with V present almost exclusively in the oxidation states +IV and +V,^1a,68^ but some authors suggested that the percent amount in the red blood cells (RBCs) could not be negligible and, for some compounds, exceed 50%.^24f,61b^ Other studies reported the possibility of reduction of V^IV^ to V^III^ in blood, and formation of the very stable adduct (V^III^)_2_(HTf) with human serum transferrin.^25e,69^

In the blood serum, VCs can interact with bLs, such as citrate, lactate, oxalate, phosphates, amino acids, etc., and proteins, like HTf and human serum albumin (HSA).^59,61a,70^ Therefore, the form with which V^IV^ reaches the target organs depends on the affinity for each bL and their concentrations. The major bLs of the serum are lactate (lact; 1.51 mM), citrate (citr; 99.0 μM), oxalate (ox; 9.20 μM), and histidine (His; 77.0 μM).^71^ In this work, model calculations using the thermodynamic stability constants of the species revealed in the systems containing V^IV^O^2+^, acac, and bL/proteins were performed to establish the chemical form of V^IV^O^2+^ in the concentration range 1 nM-1 mM.

Concerning the V concentration in the blood serum, it must be considered that for healthy humans it is in the range 1–20 nM.^1c,72^ In studies with STZ-induced diabetic rats, it was reported that upon administration by intraperitoneal injections of [V^IV^O­(ma)_2_] (bis­(maltolato)­oxidovanadium­(IV) or BMOV) or V^IV^OSO_4_, the levels of V could reach millimolar concentrations,^73^ while in other studies, upon oral treatments with V-based drugs, such as BMOV or bis­(ethylmaltolato)­oxidovanadium­(IV) (BEOV), the determined V concentrations were 1–10 μM in humans^74^ and 40–60 μM in rats.^75^ Therefore, the vanadium levels achieved in the blood serum depend on the form of administration of the VC tested. Based on clinical studies with humans, we believe that for chronic administration serum concentrations will typically be significantly below 1 mM and consider V concentrations in the range 10–20 μM as reasonable. However, we present speciation diagrams up to 1 mM concentration, in order to account for the possibility that higher vanadium levels—for example, immediately after administration—may be reached in the blood serum (and in RBCs or cell cancer cytosol, see below) is also covered. The results for the potential drug [V^IV^O­(acac)_2_] are displayed in Figure 17.

**Figure 17:**
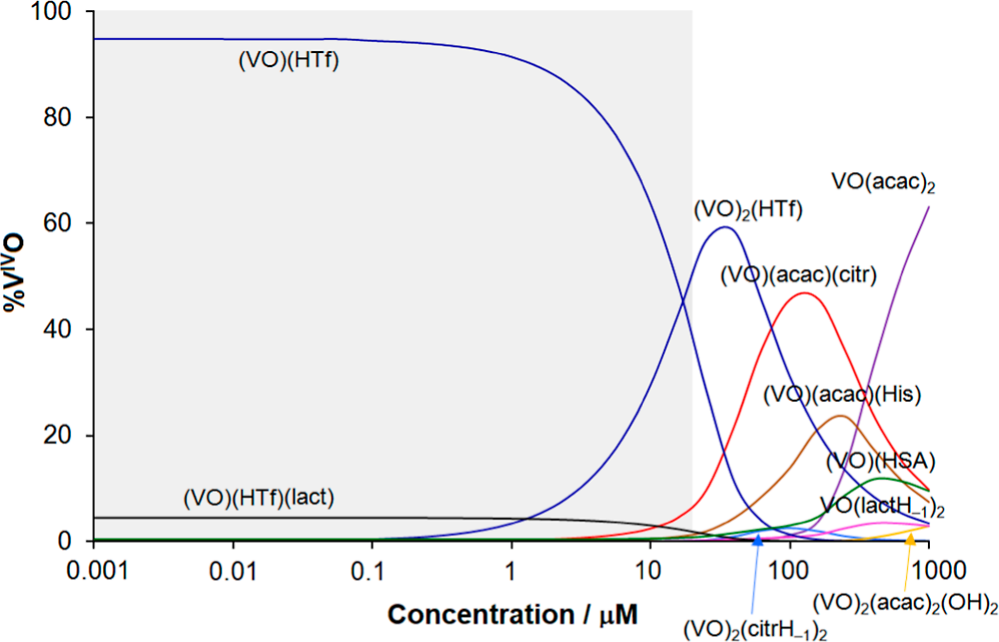
Calculated speciation in the blood serum at pH 7.4 considering addition of [V^IV^O­(acac)_2_] in the concentration range 1 nM–1000 μM. The V^IV^ concentration range below 20 μM is shown with a gray background, but it should be noted that, upon administration by intraperitoneal injections of VCs, the V concentration achieved in the blood serum may be much higher (see text). The concentration of the blood bioligands was taken from refs 71 and 76 . All the bioligand concentration and stability constants used to model the speciation are reported in Tables S2 and S3 of the Supporting Information.

Observing Figure 17, it is clear that for V concentrations above ∼ 30 μM, several V species may form, including mixed-ligand V^IV^O–acac–bL. This may suggest that, in certain conditions, V^IV^O–acac–bL complexes may be present in the blood serum for some periods of time, before being excreted. For V concentrations around 10 μM or lower, the predominant species are (V^IV^O)­(HTf) and (V^IV^O)_2_(HTf), where V^IV^O^2+^ occupies one or both the binding sites of the N- or C-terminal region of transferrin; the sum of their concentration overcomes 90%, with the remaining vanadium­(IV) amount present as (V^IV^O)­(HTf)­(lact), where lactate acts as a synergistic anion,^77^ i.e., behaving similarly to carbonate (Figure 17). It has been pointed out that the formation of (V^IV^O)­(HTf) and/or (V^IV^O)_2_(HTf) would slow down the oxidation of V^IV^ to V^V^.^61a,78^ When V concentration is larger than 10–20 μM, the formation of [V^IV^O­(acac)­(citr)]^2–^ and [V^IV^O­(acac)­(His)] starts being relevant and progressively increases in the range 50–150 μM. In particular, the percentage of [V^IV^O­(acac)­(citr)]^2–^ reaches its maximum value between 100 and 150 μM (∼45%), while that of [V^IV^O­(acac)­(His)] around 250 μM (∼25%), although these values are normally considered beyond the possible physiological concentrations. [V^IV^O­(acac)_2_] should become the major species at V^IV^ concentrations higher than 300 μM (Figure 17). So, it is the total V concentration in the serum that determines which species are formed in the blood, and this depends on the form of administration of [V^IV^O­(acac)_2_]. Based on the studies in the literature, if the concentration is below 20 μM, most of V exists (V^IV^O)­(HTf) and (V^IV^O)_2_(HTf).^39,40^ However, even in these conditions, this does not imply that the original ligand bound to the V^IV^O^2+^ ion is irrelevant; in fact, the coordinated ligand affects the lipophilicity, charge, stability, membrane permeability, and cellular uptake of the V-based drug, i.e., properties which are not captured by equilibrium speciation alone, influencing several processes that may occur before equilibrium with transferrin is reached, for example cellular uptake and bioavailability.

The results here obtained can be used to reinterpret the EPR spectra obtained when blood serum samples are incubated with [V^IV^O­(acac)_2_], discussed in ref 79. The spectra are reported in Figure 18. When the blood serum is incubated with 454.5 μM [V^IV^O­(acac)_2_] (trace b), the resonances of [V^IV^O­(acac)_2_] (**I** in traces a and b) are revealed in agreement with the prediction of Figure 17. Upon the decrease of the [V^IV^O­(acac)_2_] concentration down to 90.9 and 45.4 μM (traces c and d), the spectra were previously interpreted with the presence in the serum of (V^IV^O)­(HTf) and/or (V^IV^O)_2_(HTf) (**IV** in traces c, d, and f).^79^ However, in the light of the data here obtained, it is possible to assume that [V^IV^O­(acac)­(citr)]^2–^ may also exist in solution (**II** in traces b-e). In fact, its resonances (shown in Figure 4 and indicated with the dotted lines in Figure 18) are compatible with those detected in traces b-d, and the simultaneous presence of [V^IV^O­(acac)_2_] and [V^IV^O­(acac)­(citr)]^2–^ for V^IV^ 454.5 μM and (V^IV^O)­(HTf)/(V^IV^O)_2_(HTf) and [V^IV^O­(acac)­(citr)]^2–^ for V^IV^ in the range 45.4–90.9 μM—predicted also by the model in Figure 17—could be postulated.

**Figure 18:**
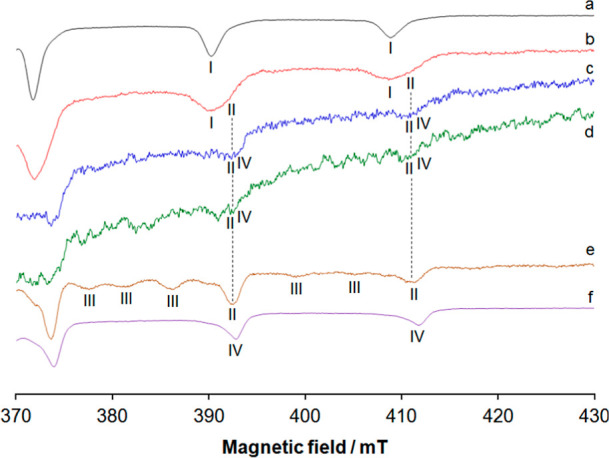
Anisotropic EPR spectra of solutions at pH 7.4 containing: (a) [V^IV^O­(acac)_2_] (*c*_V_ = 1.0 mM); (b) serum incubated with [V^IV^O­(acac)_2_] (*c*_V_ = 454.5 μM); (c) serum incubated with [V^IV^O­(acac)_2_] (*c*_V_ = 90.9 μM); (d) serum incubated with [V^IV^O­(acac)_2_] (*c*_V_ = 45.4 μM); (e) [V^IV^O­(acac)_2_]/citr with molar ratio 1:1 (*c*_V_ = 1 mM); and (f) V^IV^O^2+^/apo-HTf with molar ratio 2:1 (*c*_V_ = 500 μM). **I**, [V^IV^O­(acac)_2_]; **II**, [V^IV^O­(acac)­(citr)]^2–^; **III**, [(V^IV^O)_2_(citrH_–1_)_2_]^4–^; and **IV**, (V^IV^O)­(HTf)/(V^IV^O)_2_(HTf). The *M*_I_ = 5/2, 7/2 resonances of the mixed-ligand species [V^IV^O­(acac)­(citr)]^2–^ are also denoted with the dotted lines. All the spectra were obtained through the acquisition of an appropriate number of scans and using the signal averaging. Adapted with permission from ref 79 Copyright 2017 Elsevier.

### 3.12 Speciation of [V^IV^O­(acac)_2_] in Erythrocyte Cytosol

When vanadium species are present in the bloodstream, a non-negligible fraction can be internalized by RBCs. According to the available literature, vanadium distributes between the plasma and erythrocytes, supporting studies that highlight the importance of considering not only plasma but also RBCs when evaluating the biological and pharmacological behavior of VCs.^7,61b,80^ Overall, findings from in vivo studies in animals are consistent with in vitro experiments in which VCs are introduced into the blood. In particular, several reports indicate that, depending on the specific complex administered, a substantial fraction of vanadium—typically around 50%, and in some cases as high as 70–80%—is found in RBCs following incubation in blood.^24f,24g,61b,80^ Therefore, if a significant proportion of vanadium is sequestered by RBCs, understanding the fate of these V species during phagocytosis becomes essential, although this has been scarcely explored in the literature.^7^

The cytosol of red blood cells contains several biologically relevant ligands capable of interacting with VCs and stabilizing newly formed species. In erythrocytes, the major intracellular components capable of binding V include hemoglobin (5.1 mM), lactate (0.9 mM), histidine (0.09 mM), aspartate (0.3 mM), and ATP (1.4 mM) and, among the reducing agents, GSH (2.3 mM).^81^ Citrate is detected at low abundance in erythrocytes by metabolomic analyses, consistent with the absence of a functional tricarboxylic acid cycle in these cells;^82^ therefore, for modeling purposes, its intracellular concentration was set to 0.05 mM, i.e., within the low micromolar range expected for this metabolite.

For erythrocyte cytosol, the calculated speciation at pH 7.2, with V^IV^ concentrations from 1 nM to 1 mM, is displayed in Figure 19.

**Figure 19:**
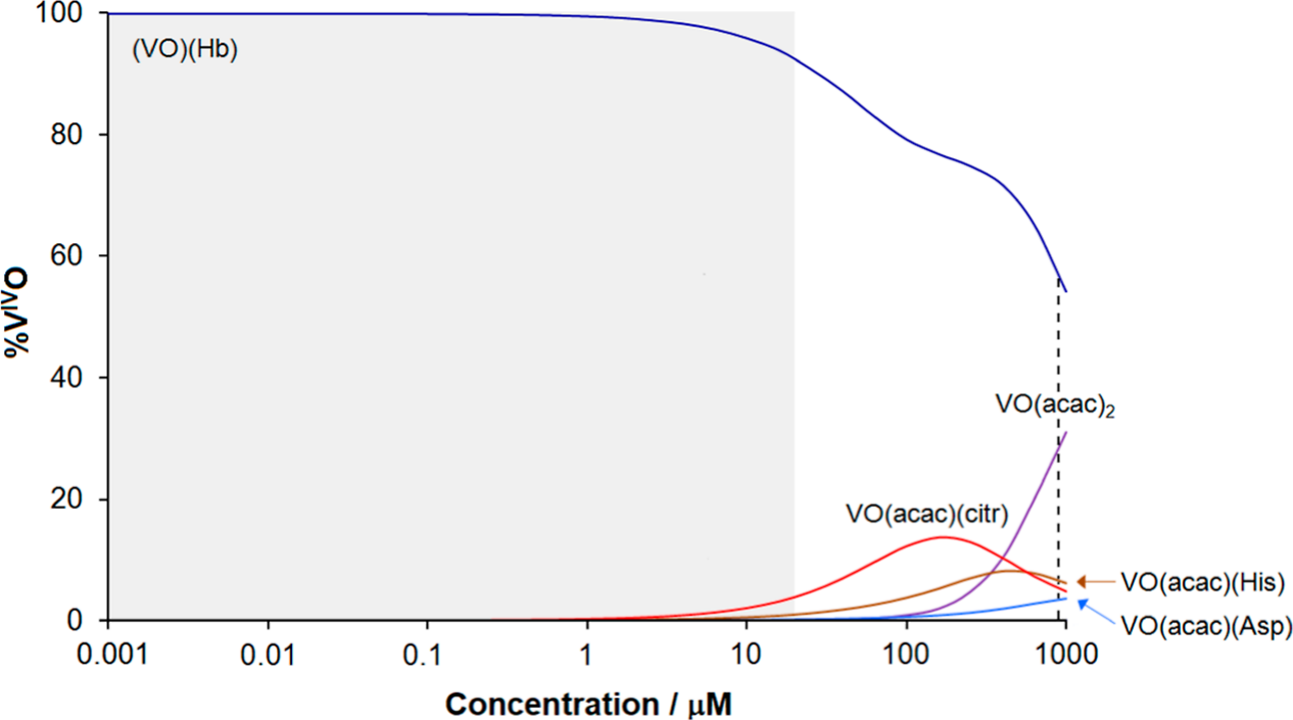
Calculated speciation in RBC cytosol at pH 7.2 considering addition of [V^IV^O­(acac)_2_] in the concentration range 1 nM–1000 μM. The V concentration range below 20 μM is shown with a gray background, while a V concentration of 0.91 mM, studied by EPR in ref 24f is denoted by the dotted line. It must considered that, upon administration by intraperitoneal injections of VCs, the V concentration achieved inside RBCs may be much higher than 20 μM (see text). The concentration of all the cytosol bioligands was taken from ref 81 and that of citrate was set to 0.05 mM. All the bioligand concentration and stability constants used to model the speciation are reported in Tables S4 and S5 of the Supporting Information.

It can be observed that, for V^IV^ concentrations lower than the physiological ones, i.e., 20 μM, the major species is (V^IV^O)­(Hb), with the V bound to the sites β and γ of hemoglobin; a minor amount, less than 10%, should be present as mixed-ligand complexes: [V^IV^O­(acac)­(citr)]^2–^ and [V^IV^O­(acac)­(His)]. With V concentrations above 20 μM, the percent amount of [V^IV^O­(acac)­(citr)]^2–^ and [V^IV^O­(acac)­(His)] increases, and it reaches ca. 20% when added [V^IV^O­(acac)_2_] is 160 μM. From a V concentration of 100 μM, the formation of [V^IV^O­(acac)­(Asp)]^−^ and [V^IV^O­(acac)_2_] starts being relevant, and this latter arrives up to ca. 30% when V is 1 mM (Figure 19).

The quality of the model can be tested by comparing the experimental EPR spectra recorded on RBC incubated with [V^IV^O­(acac)_2_] with a concentration of 0.91 mM, discussed previously in the literature.^24f^ The spectra are displayed in Figure 20. The results can be interpreted considering that when the RBCs are incubated with only vanadate­(V), three species are detected: (i) the adduct (V^IV^O)­(Hb) (**IV** in trace d) with V^IV^ bound to the sites β ((His146, Glu90, and Asp94; H_2_O) coordination, according to the covalent docking calculations^83^) and γ ((His116, His117, and Glu26; H_2_O) coordination^83^); (ii–iii) the two species V^IV^O–bL^1^ and V^IV^O–bL^2^ (**II** and **III**), not yet identified in the literature, where bL can be citrate, a carbohydrate, a 2-hydroxyacid derivative, or a membrane protein.^24f,80^ It must be noted that the ratio between the spectral intensity of these species remains the same, independently of the studied V system. When the erythrocytes are incubated with [V^IV^O­(acac)_2_], the resonances of this latter species add to the spectrum (**I** in trace c). In particular, the *M*_I_ = 5/2, 7/2 resonances at 390.4 and 408.6 mT, denoted by the dotted line in Figure 20, and not detected in the absence of [V^IV^O­(acac)_2_], are well evident. Notably, the intensity of the absorptions of [V^IV^O­(acac)_2_] is slightly lower than that of (V^IV^O)­(Hb), in agreement with the prediction shown in Figure 19, where the used concentration, 0.91 mM, is shown by the dotted line. The percentages predicted for (V^IV^O)­(Hb) and [V^IV^O­(acac)_2_], based on our model, are 55% and 31%, which would correspond to a ratio of the EPR intensities of ca. 1.8.

**Figure 20:**
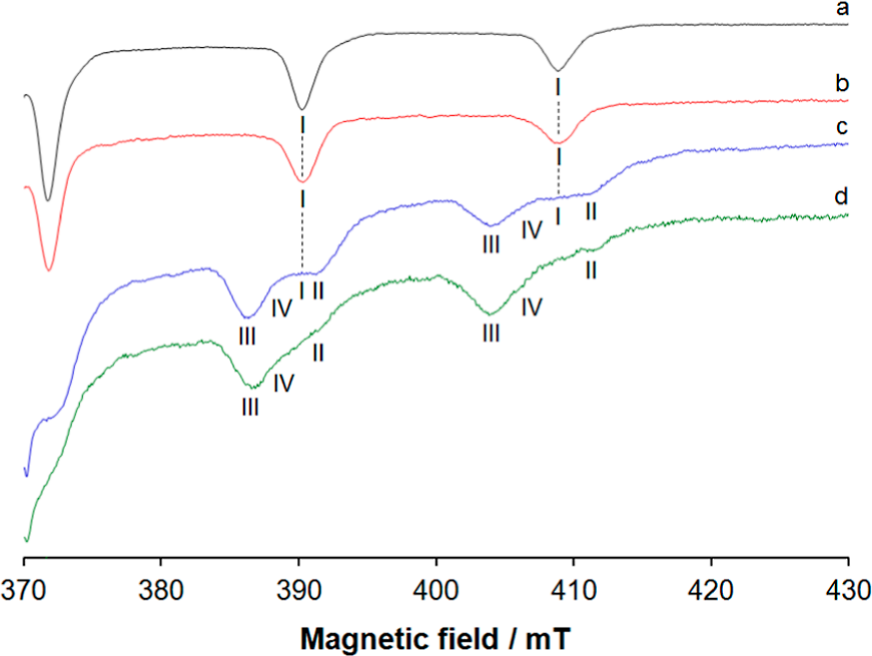
Anisotropic EPR spectra of solutions containing: (a) [V^IV^O­(acac)_2_] (*c*_V_ = 1.0 mM); (b) [V^IV^O­(acac)_2_]/Hb with molar ratio 2:1 (*c*_V_ = 0.62 mM); (c) lysate samples of RBC incubated with [V^IV^O­(acac)_2_] for 1 h (*c*_V_ = 0.91 mM); and (d) lysate samples of RBC incubated with vanadate­(V) for 1 h (*c*_V_ = 0.91 mM). **I**, [V^IV^O­(acac)_2_]; **II**, V^IV^O–bL^1^; **III**, V^IV^O–bL^2^; and **IV**, (V^IV^O)­(Hb). The *M*_I_ = 5/2, 7/2 resonances of the species [V^IV^O­(acac)_2_] are also denoted with the dotted lines. All the spectra were obtained through the acquisition of an appropriate number of scans and using the signal averaging. Adapted from ref 24f Copyright 2014 American Chemical Society.

To have a final overall correct vanadium speciation, the identity and stability of the two species, V^IV^O–bL^1^ and V^IV^O–bL^2^, must be ascertained to improve the model and predict the fate of [V^IV^O­(acac)_2_] in the cytosol of erythrocytes, but this improvement would not change the observed ratio (V^IV^O)­(Hb)/[V^IV^O­(acac)_2_] which should remain close to that detected in Figure 20.

### 3.13 Speciation of [V^IV^O­(acac)_2_] in the Cancer Cells

Considering the anticancer action of [V^IV^O­(acac)_2_], it is important to also model its speciation in cancer cells. It is known that upon incubation of A2780 ovarian cells with [V^IV^O­(acac)_2_] significant amounts of vanadium are detected inside cells.^32^ Recently, it was tested against glioblastoma through intratumoral injections;^17^ this procedure allows one to achieve high concentrations of cytotoxic drugs in the tumor, keeping their levels in the bloodstream—where hydrolysis and biotransformation processes may occur—low.^67h^ However, to date, there is no study trying to address the nature of the species formed after the administration of [V^IV^O­(acac)_2_] with these or other types of cancer cells. To this, it must be added that the composition of the cancer cells vary with the cellular line and condition of the patient and, for this reason, only a rough description can be provided here.

To model the possible speciation of [V^IV^O­(acac)_2_] in the cancer cells, the following concentrations are used (see also Table S6 of the Supporting Information): aspartate, 1.0 mM;^84^ ATP, 6 mM;^85^ citrate, 1 mM;^86^ cysteine, 50 μM;^87^ GSH, 5 mM;^88^ histidine, 50 μM;^89^ inorganic phosphate, 5 mM;^90^ lactate, 15 mM;^91^ and oxalate, 50 μM.^92^ High levels of pyrophosphate are characteristic of cells in an exponential growth phase, being the byproduct of numerous biosynthetic reactions (such as DNA/RNA polymerizations and protein synthesis) that are upregulated in cancer, and values between 0.1 and 2 mM are reported;^93^ following the discussion in the literature,^94^ a concentration of 50 μM was fixed. The stability constants of V^IV^O–acac, V^IV^O–bL, and V^IV^O–acac–bL species were taken from this work and literature data (Table S7 of the Supporting Information). Finally, for proteins, a total concentration of 5 mM was fixed with a value of conditional binding constant, logβ, of 9.0, which can be considered acceptable for a nonspecific binding of His-N. The pH was set to 7.3.

The results are shown in Figure 21. They suggest that, for [V^IV^O­(acac)_2_] concentrations up to 0.1 μM, V^IV^O distributes between lactate in the form [V^IV^O­(lactH_–1_)_2_]^2–^ with a α-hydroxycarboxylate coordination, 2 × (COO^–^, O^–^), [V^IV^O­(P_2_O_7_)_2_]^6–^, and (V^IV^O)­(Protein) (it must be observed that the importance of this latter species could be greater if the proteins have intermediate-strong binding modes toward V^IV^O^2+^). The importance of V^IV^O–lact and V^IV^O–P_2_O_7_ species at these V concentrations can be attributed to the very large concentration of lactate reported in cancer cells on one hand^91^ and to the binding strength of pyrophosphate toward V^IV^O^2+^ ion on the other. For V concentrations larger than 0.1 μM, the formation of two citrate-containing complexes, namely the dinuclear species [(V^IV^O)_2_(citrH_–1_)_2_]^4–^ and the mixed-ligand complex [V^IV^O­(acac)­(citr)]^2–^, plus [V^IV^O­(acac)­(P_2_O_7_)]^3–^ start being relevant and they would coexist with [V^IV^O­(lactH_–1_)_2_]^2–^ and (V^IV^O)­(Protein) in the pharmacologically relevant [V^IV^O­(acac)_2_] concentration, between 1 and 20 μM. At 20 μM, the predicted amounts of [V^IV^O­(acac)­(citr)]^2–^, [V^IV^O­(acac)­(P_2_O_7_)]^3–^, [V^IV^O­(lactH_–1_)_2_]^2–^, [V^IV^O­(P_2_O_7_)_2_]^6–^, [(V^IV^O)_2_(citrH_–1_)_2_]^4–^, and (V^IV^O)­(Protein) are ca. 41, 19, 16, 9, 6, and 5%, respectively.

**Figure 21:**
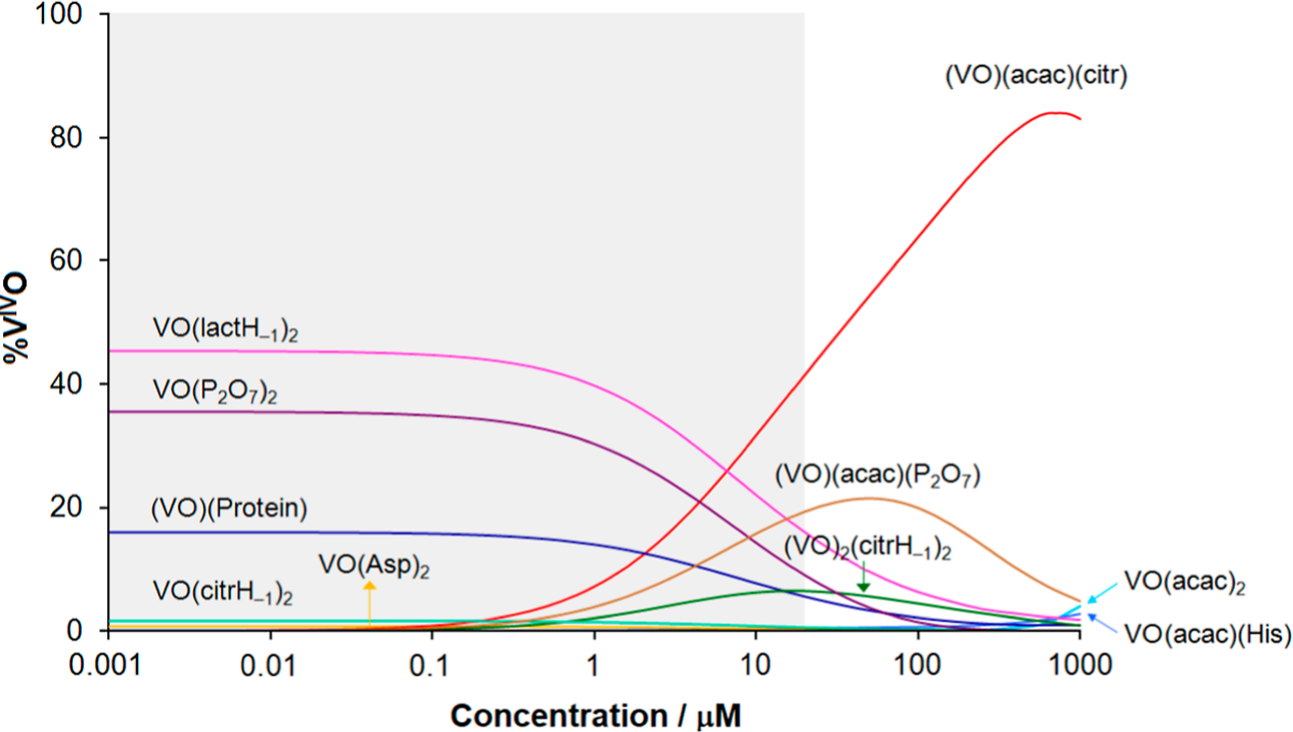
Calculated speciation in cancer cells at pH 7.3 considering the presence of [V^IV^O­(acac)_2_] in the range 1 nM–1000 μM. The V concentration range below 20 μM is shown with a gray background, but it should be observed that, upon administration by intraperitoneal or intratumoral injections of VCs, the V concentration reached inside the cancer cells may possibly be much higher than 20 μM (see text). The concentration of the bioligands was taken from refs 84–94 , while for proteins a total concentration of 5 mM was fixed. All the bioligand concentration and stability constants used to model the speciation are reported in Tables S6 and S7 of the Supporting Information.

A last comment concerns the possible formation of V^IV^O–acac–Protein adducts, demonstrated in various systems by spectroscopic and spectrometric techniques.^32,35^ A rough estimate of the conditional binding constant (logβ) for V^IV^O­(acac)­(Protein) from literature data^35b^ is 14.5 ± 2.0, the exact value depending on the binding site(s) available on the protein. However, this constant can be used to model speciation in cancer cells to account for the formation of V^IV^O­(acac)­(Protein) species inside cells. The results are presented in Figure S51 of the Supporting Information and must be considered as a preliminary description to be improved with more accurate determination of logβ for plausible V^IV^O­(acac)­(Protein) species and, also, for V^IV^O­(acac)_2_(Protein). From this approximate analysis, it emerges that up to 20–30 μM V concentration, the model coincides with that shown in Figure 21 without considering the formation of V^IV^O­(acac)­(Protein) species. The formation of V^IV^O–acac–Protein adducts should be relevant only for V concentrations larger than 100 μM in conjunction with the involvement of acac^–^ in the coordination of the V^IV^O^2+^ ion.

## 4 Conclusions

Understanding how metal-based therapeutic drugs behave and undergo changes in biological media, particularly in the case of labile metal complexes, is essential if we want to identify the active species and propose plausible mechanisms of action. It is known that if a vanadium compound is added to a water-containing environment, it will undergo several types of transformations, such as ligand exchange, hydrolysis, and redox processes, these depending on the characteristics of the media and of the compound, as well as on its concentration.^7^ Moreover, once a VC, or any other labile metal complex, is added to a biological fluid that contains several potential ligands, the nature of the species that predominate may significantly differ from the original compound added. Consequently, the complexity of factors to be analyzed when trying to discuss any type of biological effect observed normally corresponds to a difficult task.

This work provides the first systematic and comprehensive study of the ternary systems formed by [V^IV^O­(acac)_2_] with relevant low-molecular-mass blood and intracellular bioligands. Moreover, it highlights the changes that any metal compound may undergo once inside the human body and how these may be anticipated. Upon the use of an integrated multitechnique approach based on pH-potentiometry, mass spectrometry, EPR, UV–vis, and CD spectroscopy, we demonstrated the robustness and capacity of [V^IV^O­(acac)_2_] to form stable mixed-ligand complexes with biologically relevant ligands, these insights being essential for understanding how it may survive, transform, and act in vivo.

The stability constants of [V^IV^O­(acac)]^+^ and [V^IV^O­(acac)_2_] confirm that acetylacetonate forms very stable V^IV^O^2+^ complexes, but—in addition—the hydrolytic dinuclear species [(V^IV^O)_2_(acac)_2_(OH)_2_] was also detected from pH 5 to 9. Mixed-ligand species V^IV^O­(acac)­(bL) were observed with lactate, citrate, oxalate, histidine, cysteine, aspartate, pyrophosphate, ATP, and GSH. Speciation diagrams show that ternary complexes predominate around physiological pH for the systems containing citrate, histidine, aspartate, pyrophosphate, and ATP. Moreover, the potentiometric data reveal that citr-, His-, and ATP-based mixed-ligand complexes are particularly stable, suggesting their potential biological relevance. In particular, [V^IV^O­(acac)­(citr)]^2–^ reaches a significant amount when *c*_V_ = 20–50 μM in the blood serum and RBCs, and it is the major species in the model applied to cancer cells in the mentioned concentration range. Notably, EPR spectra recorded on real blood serum samples display characteristic resonances attributable to the [V^IV^O­(acac)­(citr)]^2–^ complex, providing direct experimental confirmation that such mixed-ligand species can persist and dominate when the [V^IV^O­(acac)_2_] concentration is between 50 and 100 μM, in agreement with the speciation models obtained using the bL amounts and thermodynamic stability constants of the compounds formed in biological fluids.

From this study, it is clear that the type of species formed upon administration of [V^IV^O­(acac)_2_] (and of other VCs) strongly depends on the total V concentration achieved in the biological system considered. At low μM concentrations, the original ligand bound to the V^IV^O^2+^ ion will mostly be lost; this is what is globally predicted when a VC is administered orally for long periods of time. However, if the VC is given by injection, the individual doses may be significantly higher, the total concentration in blood and cytosol may be in the mM range,^73^ and the presence of binary and mixed-ligand V^IV^O complexes containing the original ligand and/or some specific bioligand may become relevant. This helps explaining the finding of several reports that the administration of VCs is beneficial when compared with the use of simple vanadium salts (e.g., V^IV^OSO_4_).^67^

Overall, this work clarifies the solution chemistry of [V^IV^O­(acac)_2_] in biologically relevant environments and demonstrates its adaptive coordination behavior toward diverse donor sets. If introduced into biological media in some type of encapsulated form or not, the present results strongly suggest that the pharmacological activity of [V^IV^O­(acac)_2_] may derive, not only from the intact complex but also from its capacity to form V^IV^O–acac–bL compounds under physiological conditions. Therefore, the formation of these mixed-ligand complexes should be considered in the studies on the anticancer activity of [V^IV^O­(acac)_2_], keeping in mind that such species could play a not negligible role in transport, bioavailability, and molecular mechanisms of this V-based therapeutic compound.

## Supplementary Material



## Notes

Table with species derived from ligands, metal ions, and binary V^IV^O complexes identified by ESI-MS spectrometry, bioligand concentration and stability constants used to model the speciation of [V^IV^O­(acac)_2_] in the blood serum and cytosol, ESI-MS­(+) spectra recorded on the V^IV^O^2+^/acac and V^IV^O^2+^/acac/bL systems, experimental and calculated isotopic pattern for the peak of [V^IV^O­(acac)_2_] and V^IV^O–acac–bL mixed-ligand species, anisotropic EPR spectra of the V^IV^O^2+^/acac/bL systems, visible absorption and CD spectra recorded on the V^IV^O^2+^/acac/bL systems, and calculated speciation in cancer cells of [V^IV^O­(acac)_2_] assuming the formation of V^IV^O­(acac)­(Protein) (PDF)
