# Supplementary material for: Mixed-Ligand Complex Formation in the Systems Containing the Potential Drug [VIVO(acac)2] with Blood and Cellular Bioligands. Implications on the Active Species Formed in the Organism
Source: Inorg Chem. 2026 Jul 22;65(31):18041–63. doi: 10.1021/acs.inorgchem.6c02223 (PMC13463689; doi:10.1021/acs.inorgchem.6c02223)
Supplement: Supplementary file 1 [file ic6c02223_si_001.pdf]

SUPPORTING INFORMATION

Mixed-ligand Complex Formation in the Systems Containing the Potential Drug [$V^{IV}O(acac)_2$] with Blood and Cellular Bioligands. Implications on the Active Species Formed in the Organism

Daniele Sanna,[a] Valeria Ugone,[a] Péter Buglyó,[b] Nóra Ildikó Kovács,[b] Sándor Nagy,[b] Nádia Ribeiro,[c,d] Isabel Correia,[c] João Costa Pessoa,[c]* Federico Pisanu,[e] Eugenio Garribba,[e]*

[a] Consiglio Nazionale delle Ricerche, Istituto di Chimica Biomolecolare, Traversa La Crucca 3, I-07100 Sassari, Italy

[b] Department of Inorganic and Analytical Chemistry, University of Debrecen, H-4032 Debrecen, Egyetem tér 1, Hungary

[c] Centro de Química Estrutural, Institute of Molecular Sciences and Departamento de Engenharia Química, Instituto Superior Técnico, Universidade de Lisboa, Av. Rovisco Pais, 1049-001 Lisboa, Portugal

[d] Now at Prof. Silva Pereira's Applied and Environmental Mycology Group (AEM), Instituto de Tecnologia Química e Biológica (ITQB), Av. da República, 2780-157 Oeiras, Portugal

[e] Dipartimento di Medicina, Chirurgia e Farmacia, Università di Sassari, Viale San Pietro, I-07100 Sassari, Italy

***** Correspondence to:
João Costa Pessoa (joao.pessoa@ist.utl.pt)
Eugenio Garribba (garribba@uniss.it)

Table S1. Species derived from ligands, free metal ions and binary $V^{IV}O$ complexes identified by ESI-MS spectrometry in the studied systems.

Ion	Composition	Exptl m/z [a]	Calcd m/z [a]	Error (ppm) [b]
$[acac+2H]^+$	$C_5H_9O_2$	101.0602	101.0597	4.7
$[acac+H+Na]^+$	$C_5H_8O_2Na$	123.0420	123.0417	2.6
$[V^{IV}O(acac)_2+H]^+$	$C_{10}H_{15}O_5V$	266.0355	266.0354	0.6
$[V^{IV}O(acac)_2+Na]^+$	$C_{10}H_{14}O_5VNa$	288.0175	288.0173	0.7
$[V^{IV}O(acac)_2–H]^–$	$C_{10}H_{13}O_5V$	264.0212	264.0208	1.5
$[H_2V^VO_4]^–$ [c]	H_2O_4V	116.9388	116.9398	-9.1
$[lact+H+Na]^+$	$C_3H_6O_3Na$	113.0213	113.0209	3.5
$[lact]^–$	$C_3H_5O_3$	89.0232	89.0244	-13.8
$[V^VO_2(lact)–H]^–$	$C_3H_4O_5V$	170.9497	170.9504	-4.2
$[citr+3H+Na]^+$	$C_6H_8O_7Na$	215.0164	215.0162	0.8
$[(V^{IV}O)_2(citr)_2]^{2–}$	$C_{12}H_{10}O_{16}V_2$	255.9432	255.9430	0.9
$[ox+H]^–$	C_2O_4H	88.9868	88.9880	-13.9
$[V^VO_2(ox)]^–$	C_2O_6V	170.9133	170.9140	-4.3
$[HHis+H]^+$	$C_6H_{10}N_3O_2$	156.0769	156.0768	1.2
$[HHis+Na]^+$	$C_6H_9N_3O_2Na$	178.0589	178.0587	0.9
$[His]^–$	$C_6H_8N_3O_2$	154.0614	154.0622	-5.1
$[H_2Cys+H]^+$	$C_3H_8NO_2S$	122.0274	122.0270	3.0
$[H_2Cys+Na]^+$	$C_3H_7NO_2SNa$	144.0092	144.0090	1.3
$[HCys]^–$	$C_6H_8N_3O_2$	120.0114	120.0125	-8.9
$[H_2Asp+H]^+$	$C_4H_8NO_4$	134.0451	134.0448	2.2
$[H_2Asp+Na]^+$	$C_4H_7NO_4Na$	156.0269	156.0267	1.2
$[HAsp]^–$	$C_4H_6NO_4$	132.0292	132.0302	-7.6
$[H_3ATP]^–$	$C_{10}H_{15}O_{13}N_5P_3$	505.9888	505.9885	0.7
$[H_2ATP]^{2–}$	$C_{10}H_{13}O_{14}N_5P_3$	252.4907	252.4906	0.2
$[V^{IV}O(ATP)]^{2–}$	$C_{10}H_{12}O_{14}N_5P_3V$	284.9525	284.9522	0.8
$[V^{IV}O(ATP)+H]^–$	$C_{10}H_{13}O_{14}N_5P_3V$	570.9126	570.9117	1.5
$[H_3GSH+H]^+$	$C_{10}H_{18}N_3O_6S$	308.0912	308.0911	0.4
$[H_3GSH+Na]^+$	$C_{10}H_{17}N_3O_6SNa$	330.0731	330.0730	0.2
$[H_2GSH]^–$	$C_{10}H_{16}N_3O_6S$	306.0767	306.0765	0.5
$[HGSH]^{2–}$	$C_{10}H_{15}N_3O_6S$	152.5337	152.5346	-6.4

[a] The experimental and calculated m/z values refer to the representative peak with the monoisotopic mass. [b] Deviation in ppm from the calculated values, determined as $10^6 \times$ [(Exptl m/z – Calcd m/z) / Calcd m/z]. [c] Species observed in almost all systems.

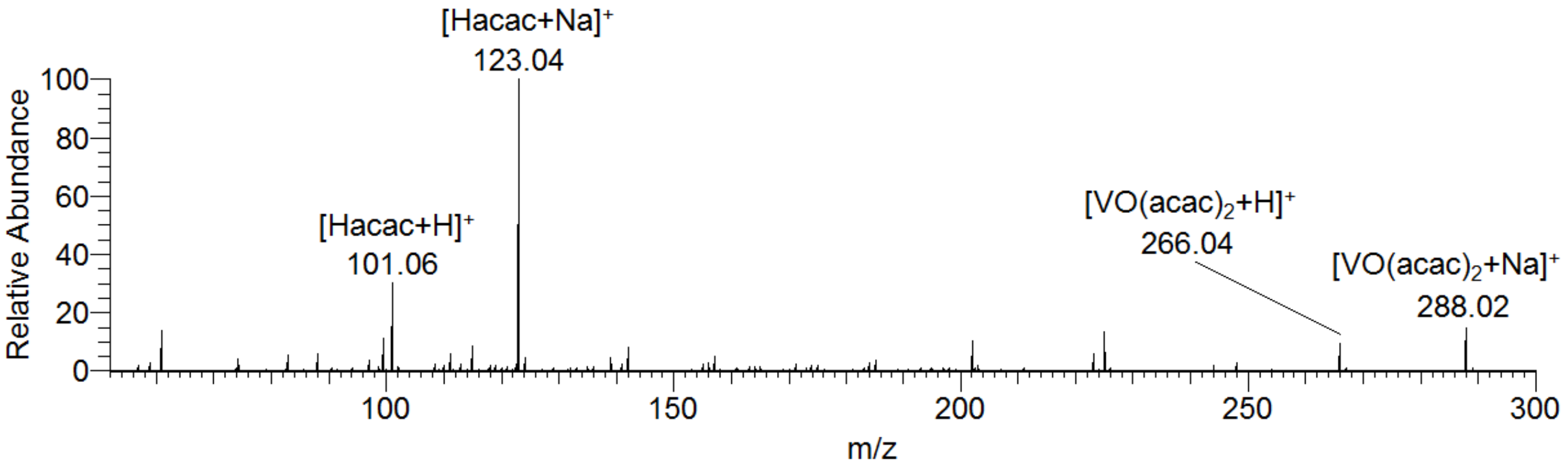

Figure S1. ESI-MS(+) spectrum of the $V^{IV}O^{2+}$/acac system with molar ratio 1:2 recorded at pH 5.50 in ultrapure LC-MS water (c_V = 5.0 × 10^{-6} M). Only two decimal numbers are reported for clarity.

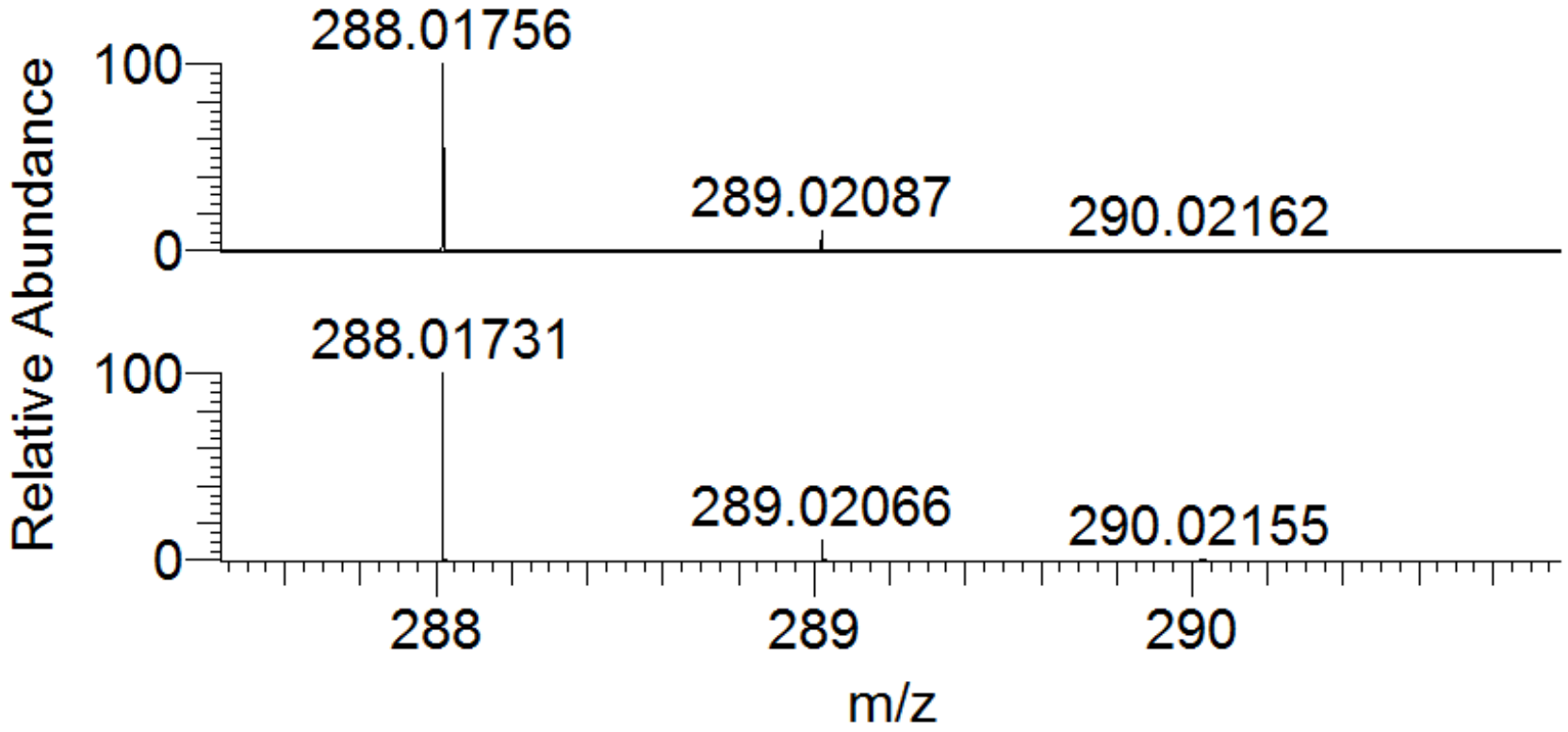

Figure S2. Experimental (above) and calculated (below) isotopic pattern for the peak of $[V^{IV}O(acac)_2+Na]^+$ revealed at m/z = 288.0176 in the positive ESI-MS spectrum recorded on the $V^{IV}O^{2+}$/acac system with molar ratio 1:2 (ultrapure LC-MS water, pH = 5.50, $c_V = 5.0 \times 10^{-6}$ M).

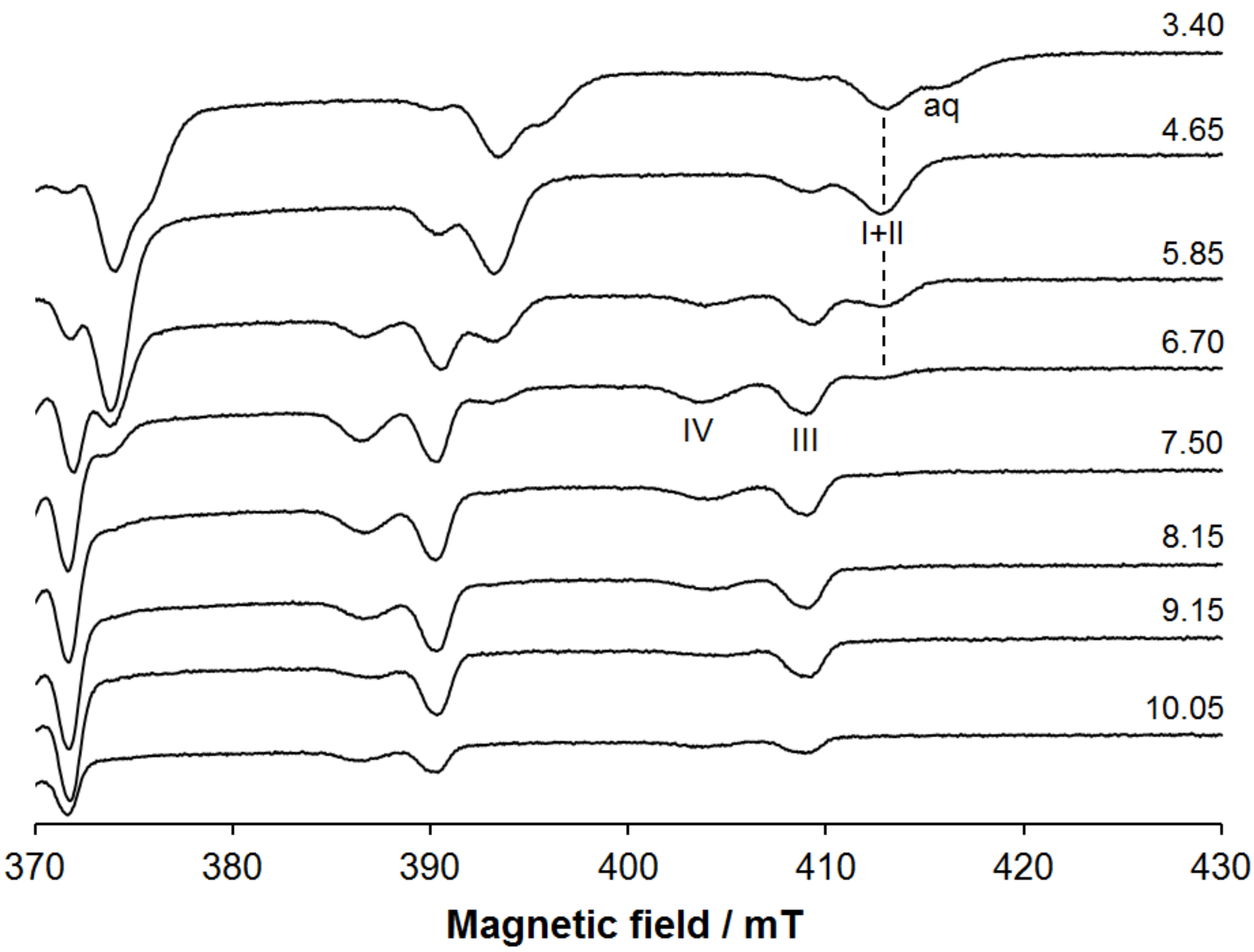

Figure S3. Anisotropic EPR spectra of solutions containing $V^{IV}O^{2+}$, acac and lact with molar ratio 1:1:1 and c_V = 2.0 × 10^{-3} M at various pH values. The resonances of these species are indicated: **aq**, $[V^{IV}O(H_2O)_5]^{2+}$; **I**, $[V^{IV}O(acac)(H_2O)_2]^+$; **II**, $[V^{IV}O(acac)(lact)]$; **III**: $[V^{IV}O(acac)_2]$; **IV**, $[V^{IV}O(lactH_{-1})_2]^{2-}$. The resonances of the mixed-ligand species $[V^{IV}O(acac)(lact)]$ are also denoted with the dotted line.

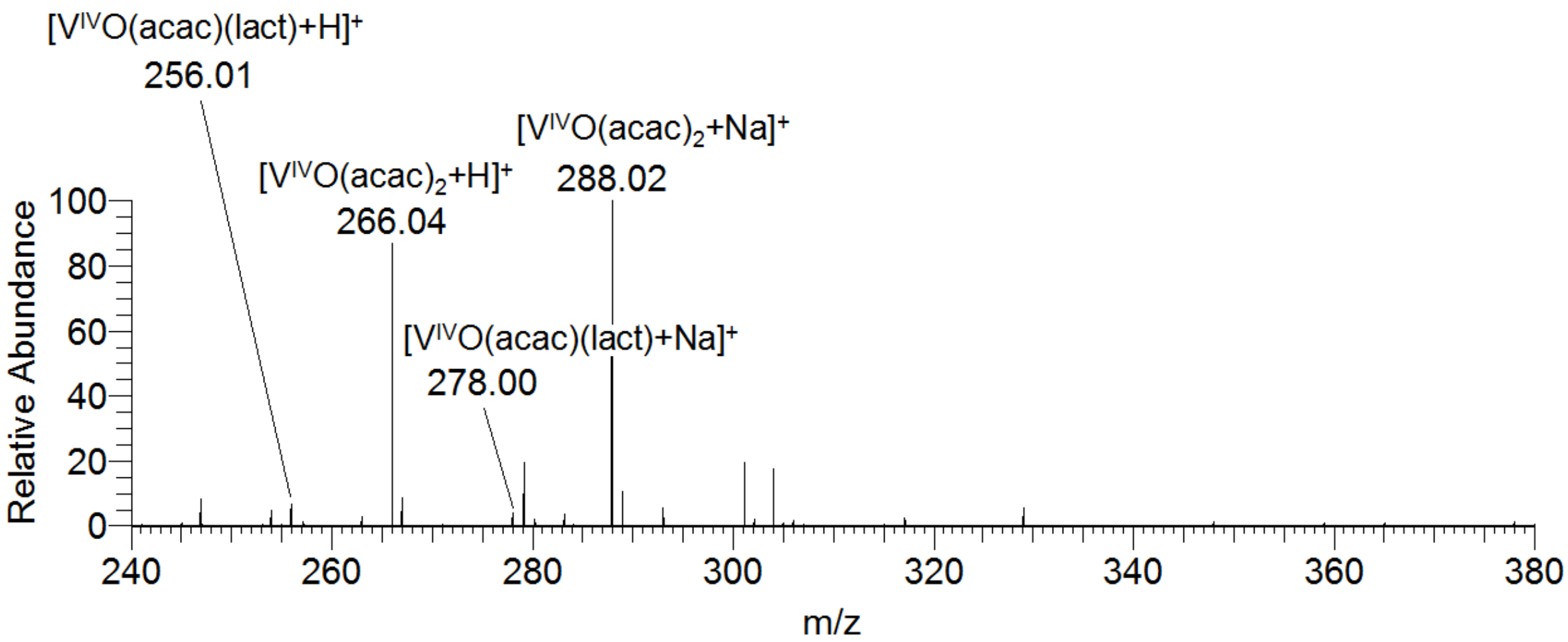

Figure S4. ESI-MS(+) spectrum of the $V^{IV}O^{2+}$/acac/lact system with molar ratio 1:2:2 recorded at pH 5.0 in ultrapure LC-MS water (c_V = 5.0 × 10^{-6} M). Only two decimal numbers are reported for clarity.

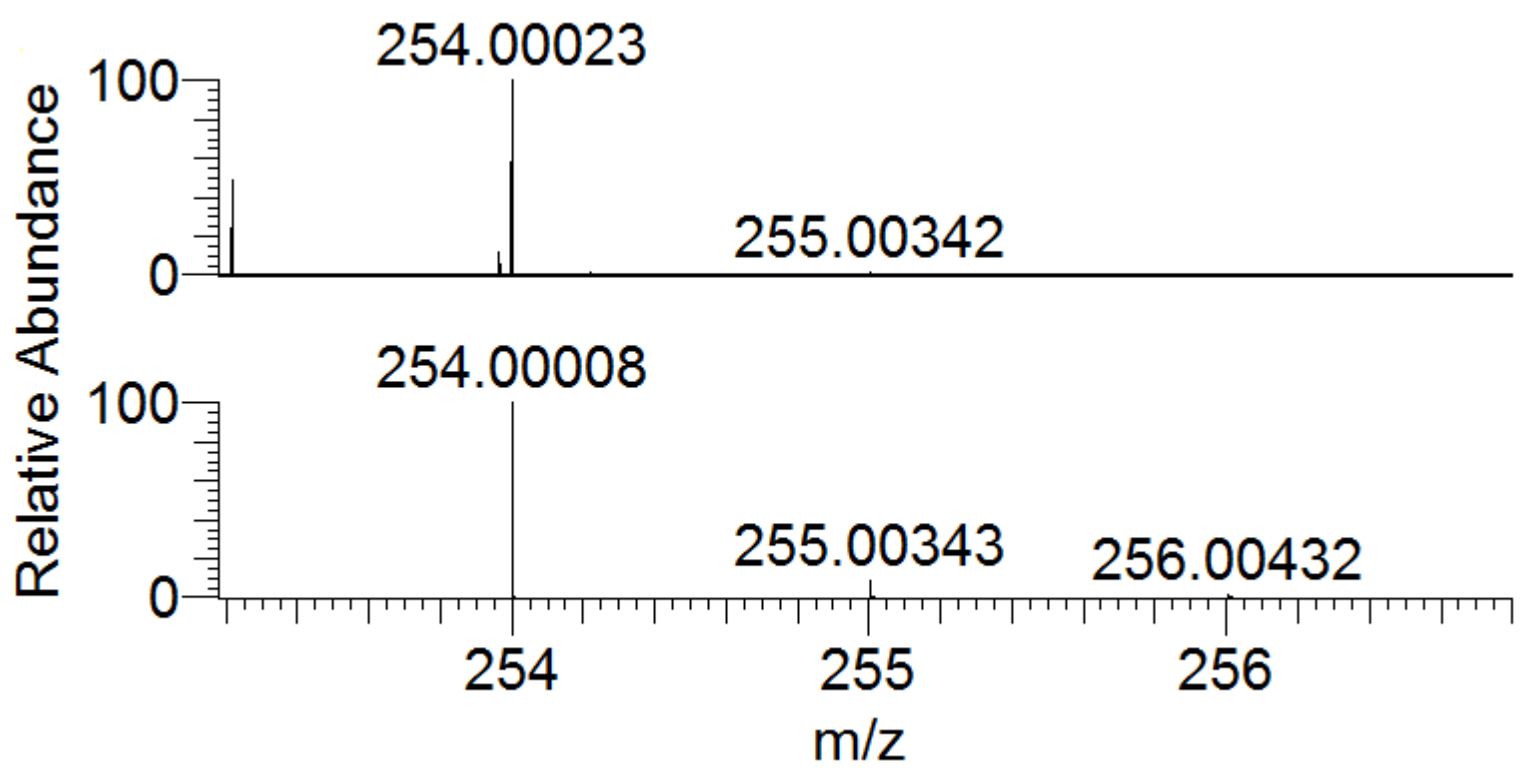

Figure S5. Experimental (above) and calculated (below) isotopic pattern for the peak of $[V^{IV}O(acac)(lact)–H]^{–}$ revealed at m/z = 254.0002 in the negative ESI-MS spectrum recorded on the $V^{IV}O^{2+}$/acac/lact system with molar ratio 1:2:2 (ultrapure LC-MS water, pH = 5.00, $c_V = 5.0 \times 10^{-6}$ M).

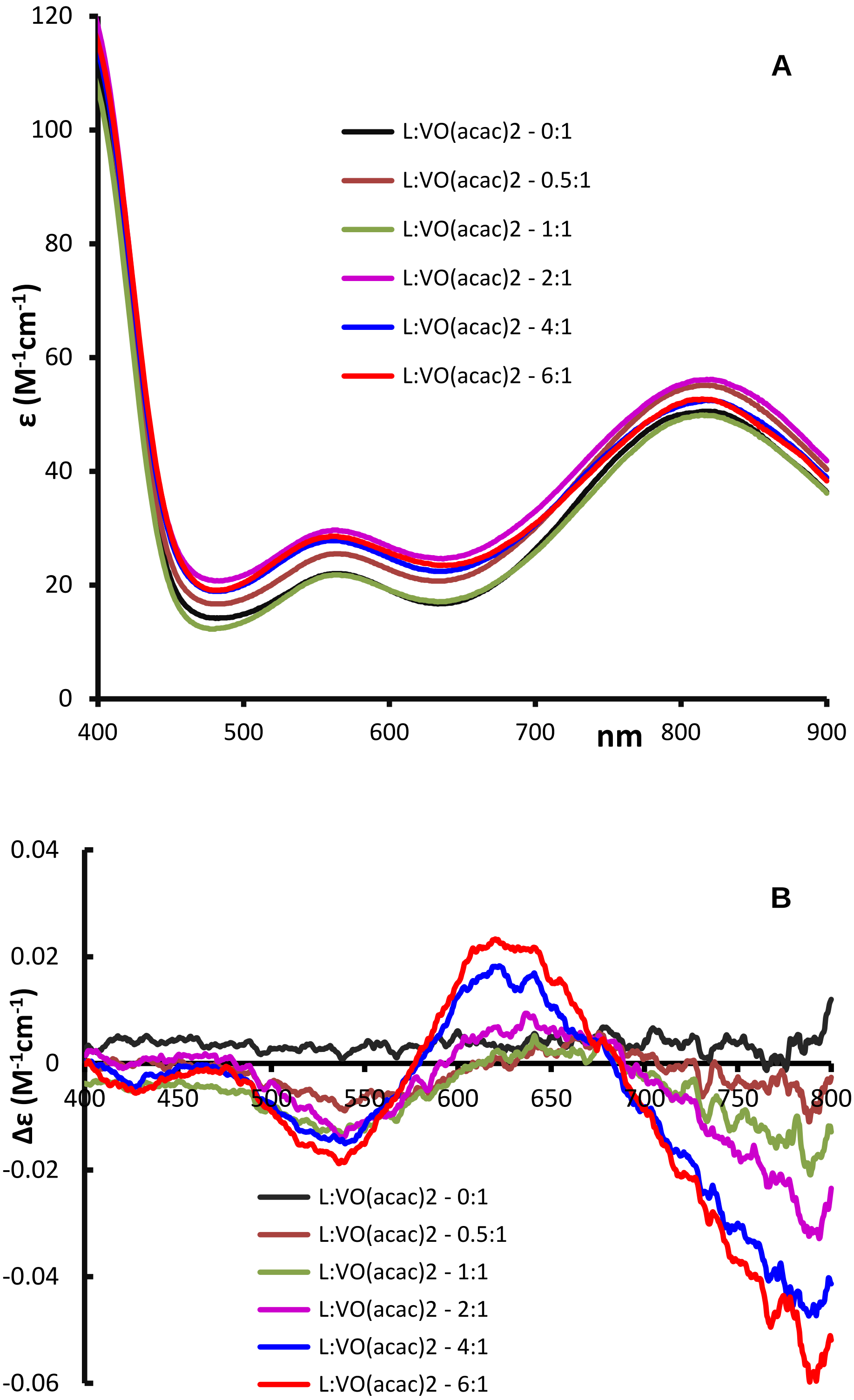

Figure S6. (A) Visible absorption and (B) circular dichroism spectra of aqueous solutions (with 2.7% MeOH) containing [$V^{IV}O(acac)_2$] and lactate (L) with $V^{IV}O$:acac:L molar ratios of 1:2:n. The value of pH = 6.0 ± 0.2 and $c_V = 2.05 \times 10^{-3}$ M. Each solution was prepared independently in 10.00 mL volumetric flasks. As no CD spectrum is expected for [$V^{IV}O(acac)_2$], the black line corresponds to the baseline.

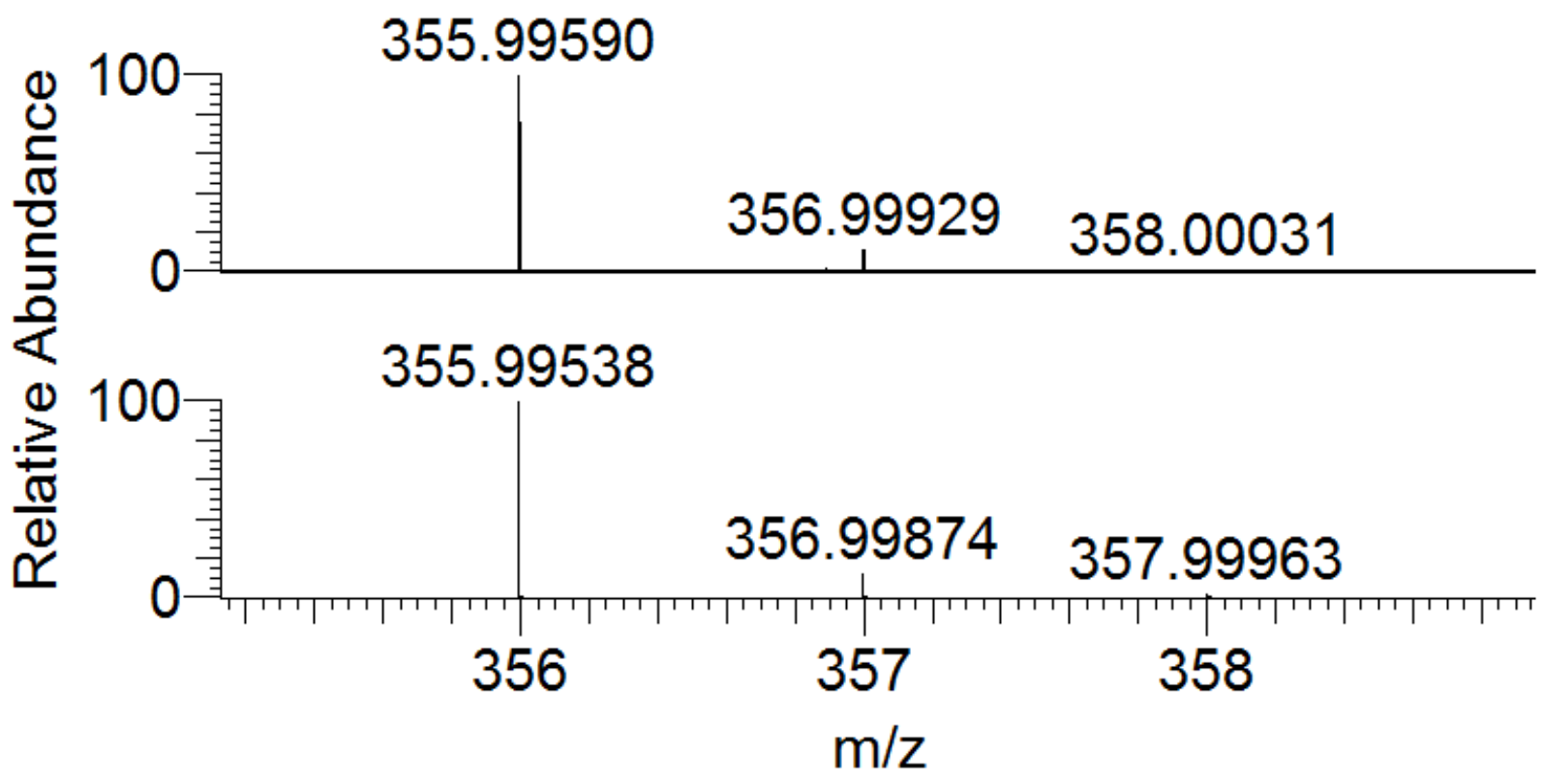

Figure S7. Experimental (above) and calculated (below) isotopic pattern for the peak of $[V^{IV}O(acac)(citr)+H]^-$ revealed at m/z = 355.9959 in the ESI-MS(–) spectrum recorded on the $V^{IV}O^{2+}$/acac/citr system with molar ratio 1:2:2 (ultrapure LC-MS water, pH = 5.15, $c_V = 5.0 \times 10^{-6}$ M).

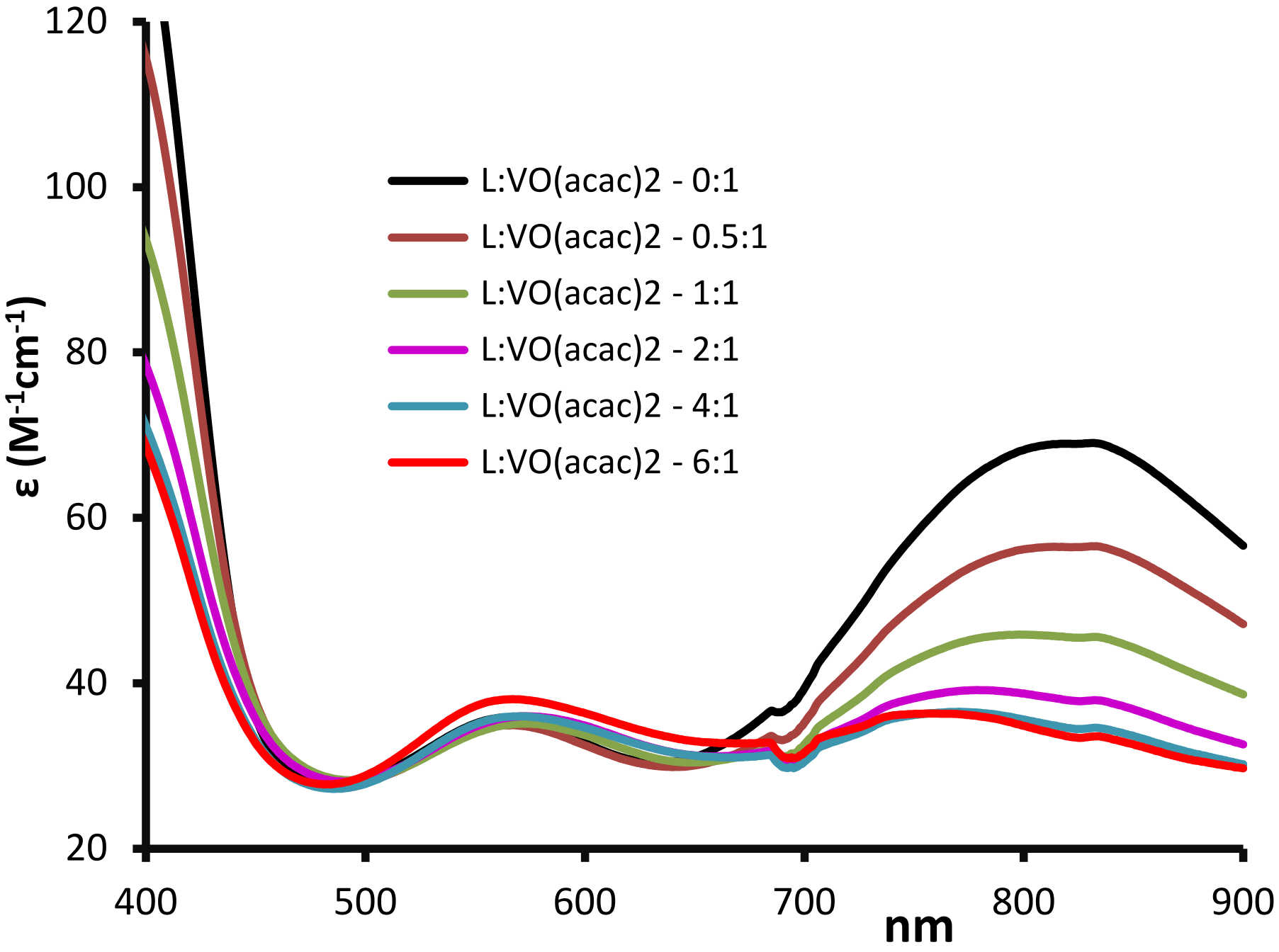

Figure S8. Visible absorption spectra of aqueous solutions (with 2.7% MeOH) containing $[V^{IV}O(acac)_2]$ and citrate (L) with VO:acac:citr molar ratios of 1:2:n. The value of pH = 5.30 ± 0.05 and $c_V = 2.05 \times 10^{-3}$ M.

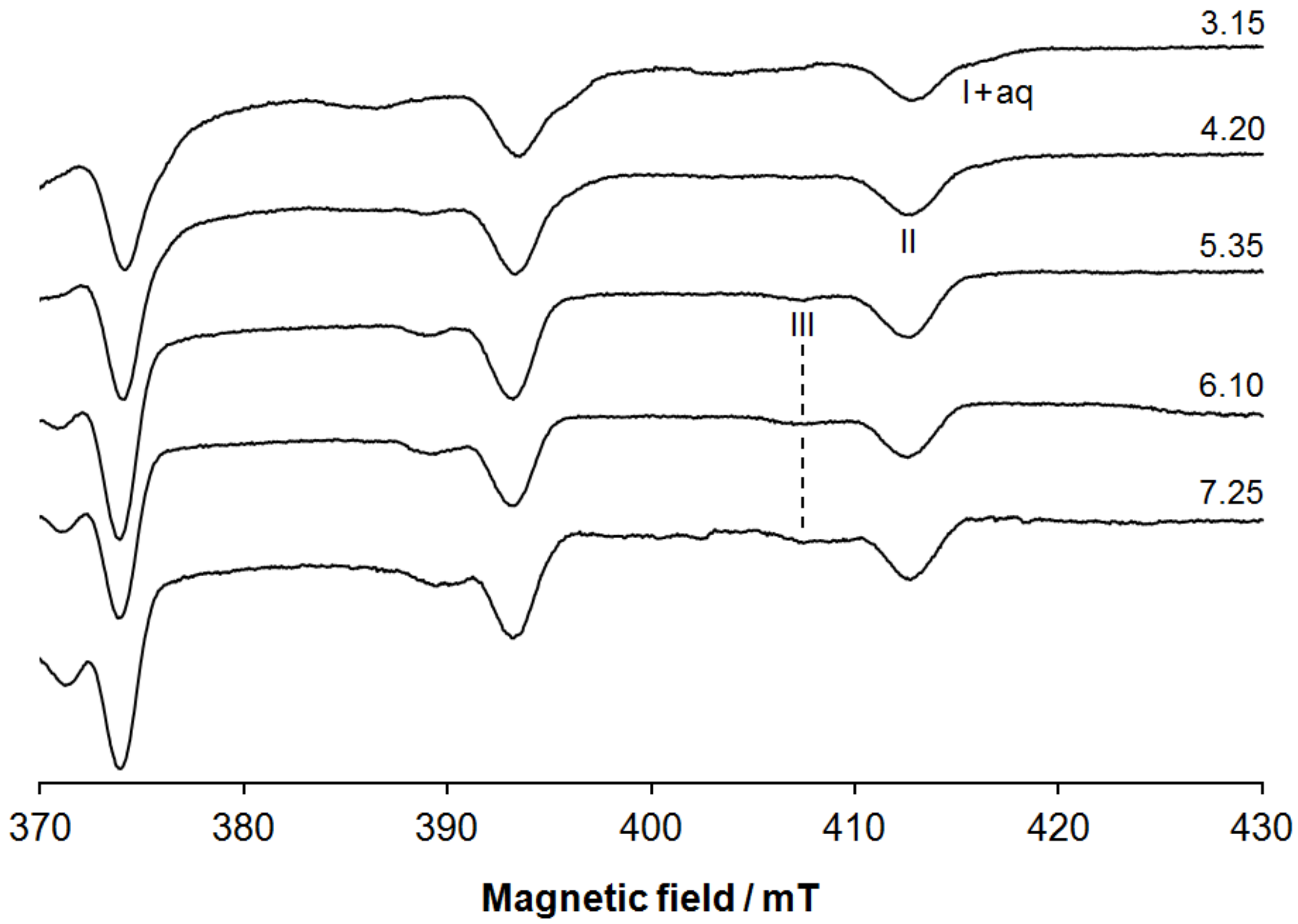

Figure S9. Anisotropic EPR spectra of solutions containing $V^{IV}O^{2+}$, acac and ox with molar ratio 1:1:1 and $c_V = 2.0 \times 10^{-3}$ M at several pH values. The resonances of these species are indicated: **aq**, $[V^{IV}O(H_2O)_5]^{2+}$; **I**, $[V^{IV}O(ox)]$; **II**, $[V^{IV}O(ox)_2(H_2O)]^{2-}$; **III**, $[V^{IV}O(acac)(ox)]^{-}$. The resonances of the mixed-ligand species $[V^{IV}O(acac)(ox)]^{-}$ are also denoted with the dotted line.

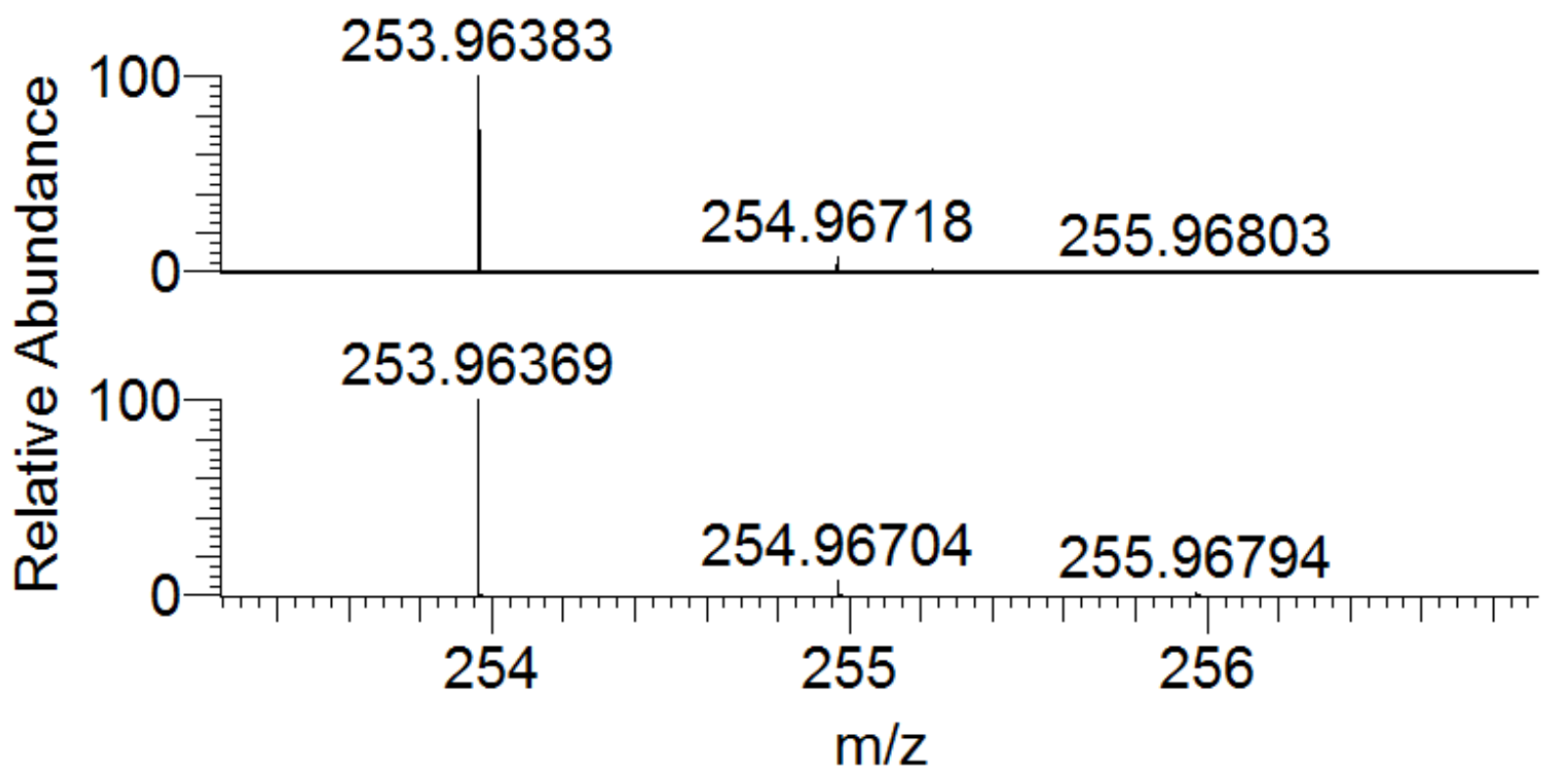

Figure S10. Experimental (above) and calculated (below) isotopic pattern for the peak of $[V^{IV}O(acac)(ox)]^{-}$ revealed at m/z = 253.9638 in the negative ESI-MS(–) spectrum recorded on the $V^{IV}O^{2+}$/acac/ox system with molar ratio 1:1:1 (ultrapure LC-MS water, pH = 4.95, $c_V = 5.0 \times 10^{-6}$ M).

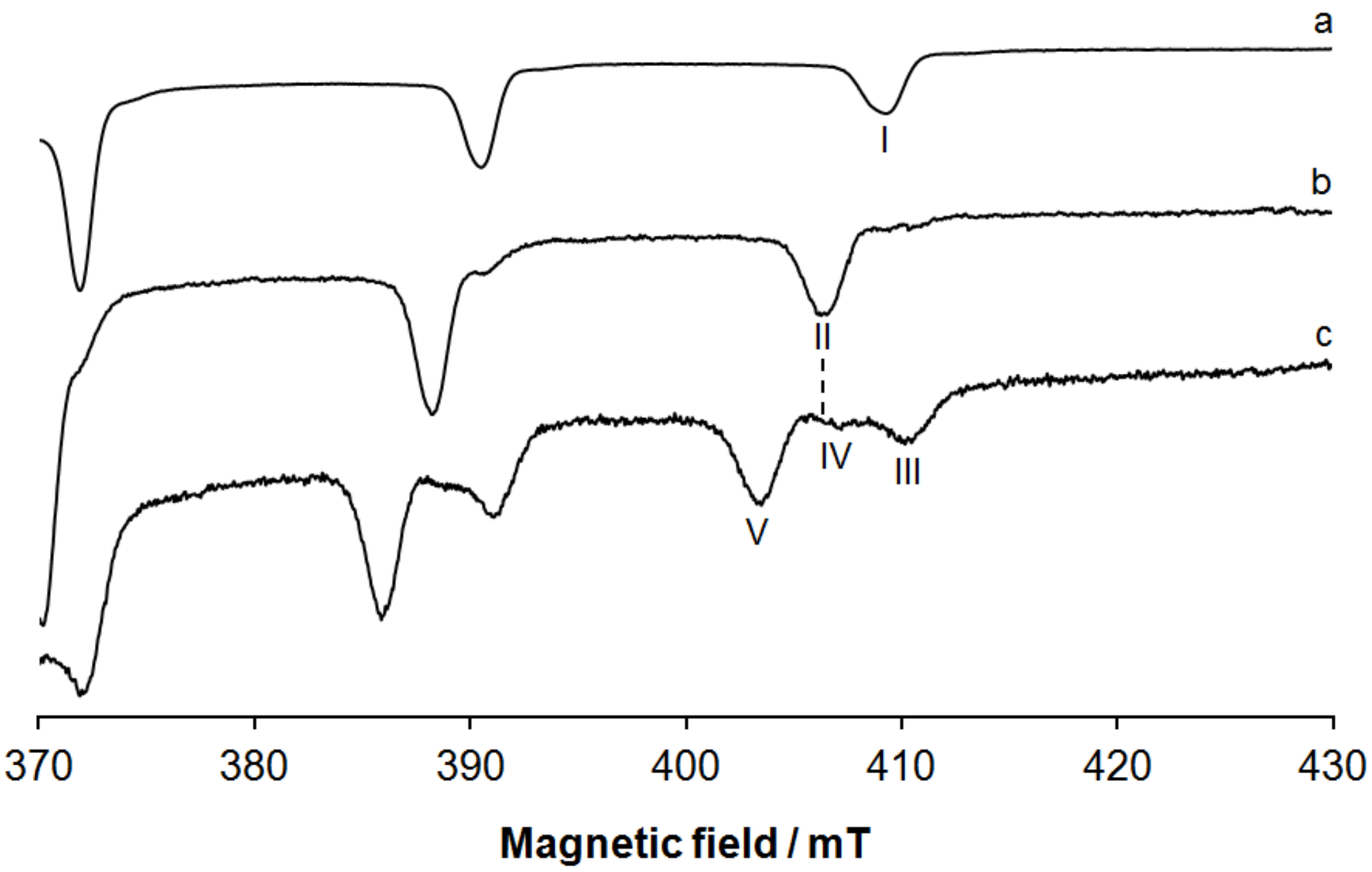

Figure S11. Anisotropic EPR spectra of solutions containing: (a) $V^{IV}O^{2+}$ and acac with a molar ratio 1:2 at pH 6.50; (b) $V^{IV}O^{2+}$, acac and His with molar ratio 1:1:1 at pH 6.50; (c) $V^{IV}O^{2+}$ and His with molar ratio 1:2 at pH 6.25. $c_V = 2.0 \times 10^{-3}$ M in all the systems. The resonances of these species are indicated: **I**, $[V^{IV}O(acac)_2]$; **II**, $[V^{IV}O(acac)(His)]$; **III**, $[V^{IV}O(His)]^+$; **IV**, $[V^{IV}O(His)_2]$ with 2 × (NH_2, COO^-) coordination of histidine; **V**, $[V^{IV}O(His)_2]$ with mixed binding mode of histidine, [(NH_2, COO^-) + (NH_2, N_{im})]. The resonances of the mixed-ligand species $[V^{IV}O(acac)(His)]$ are also denoted with the dotted line.

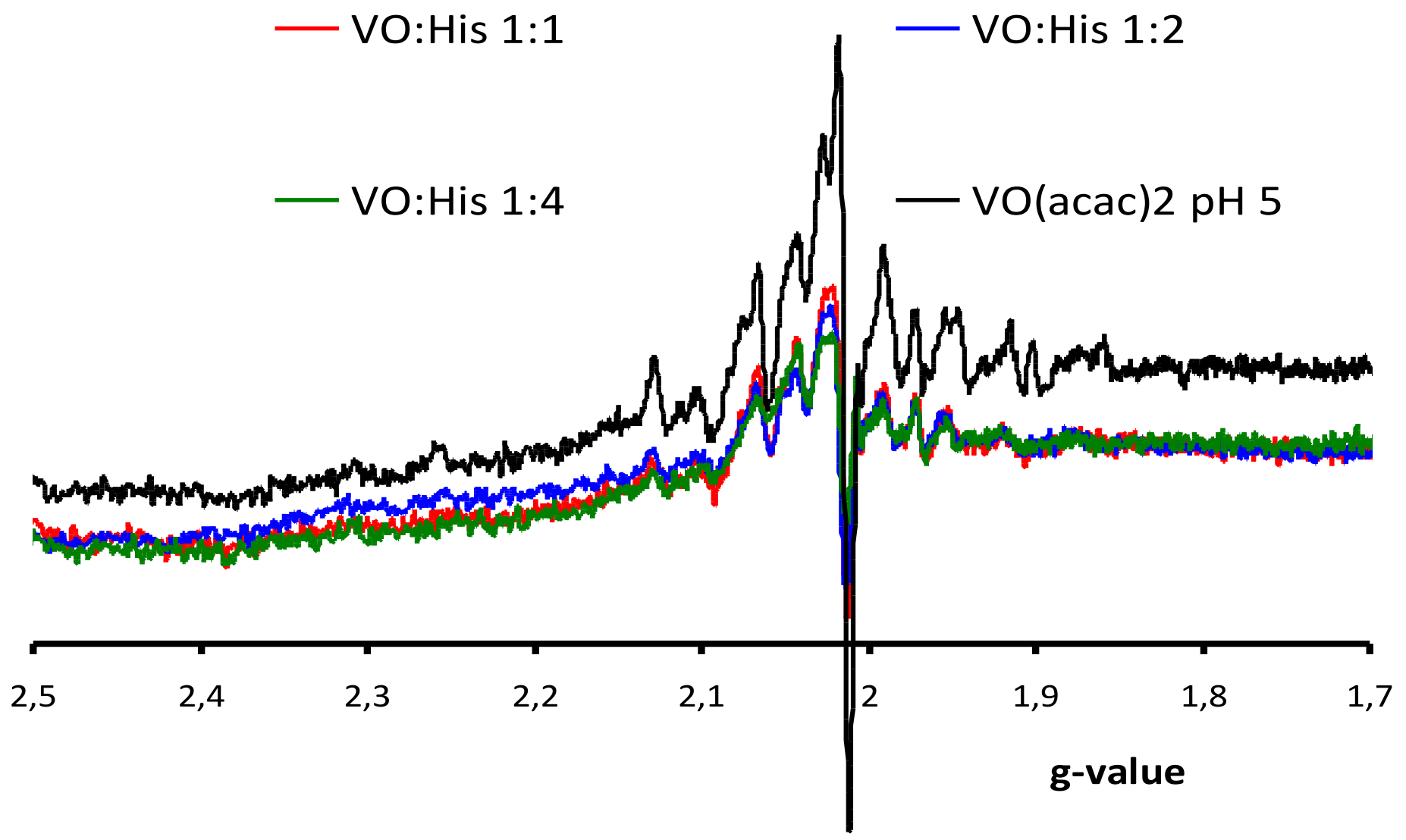

Figure S12. Anisotropic EPR spectra of solutions containing [$V^{IV}O(acac)_2$] and additions of L-His at pH = 7.0 (c_V = 2.0 × 10^{-3} M). The anisotropic EPR spectrum of a solution containing [$V^{IV}O(acac)_2$] at pH = 5 is included for comparison with those of solutions also containing His.

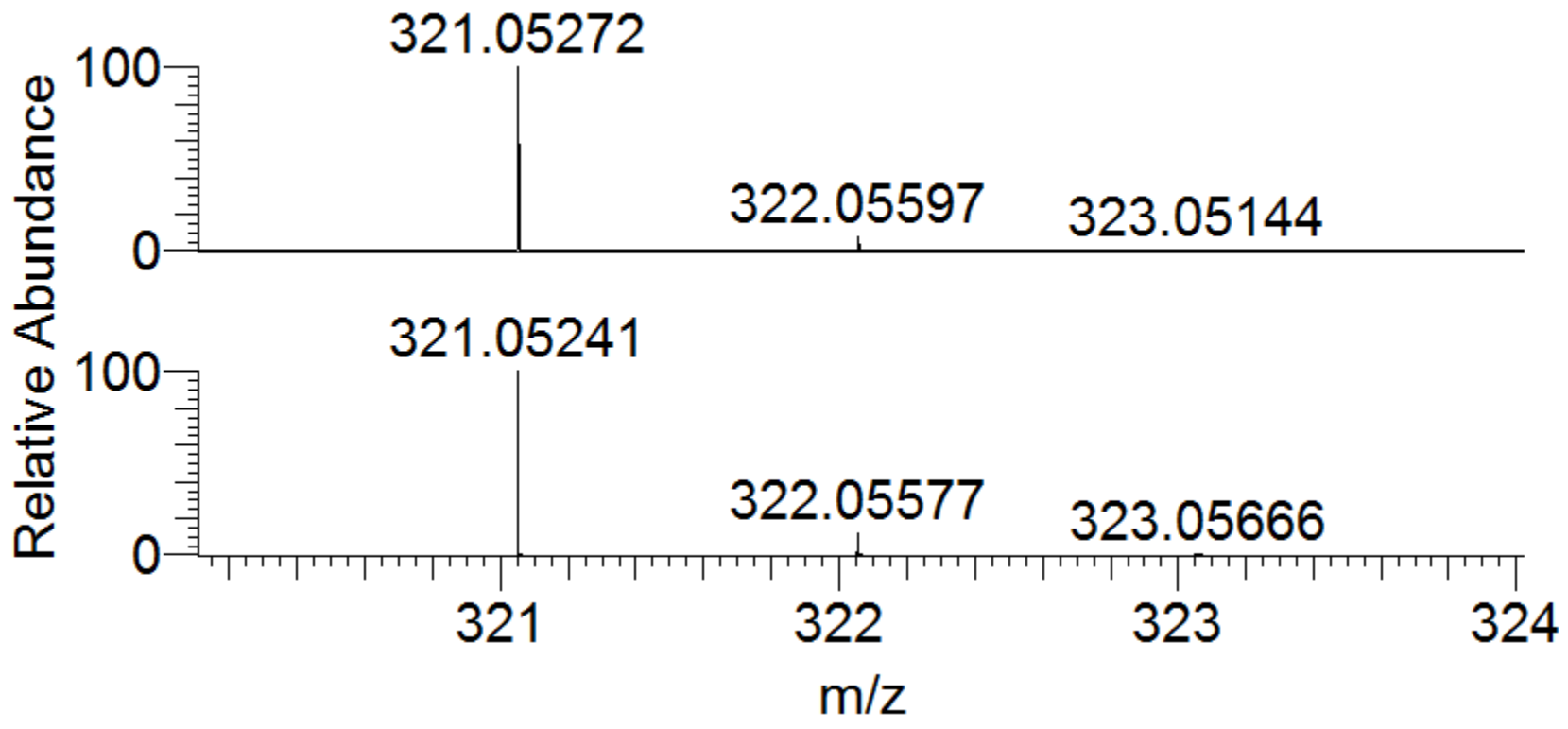

Figure S13. Experimental (above) and calculated (below) isotopic pattern for the peak of $[V^{IV}O(acac)(His)+H]^+$ revealed at m/z = 321.0527 in the ESI-MS(+) spectrum recorded on the $V^{IV}O^{2+}$/acac/His system with molar ratio 1:2:2 (ultrapure LC-MS water, pH = 7.20, $c_V = 5.0 \times 10^{-6}$ M).

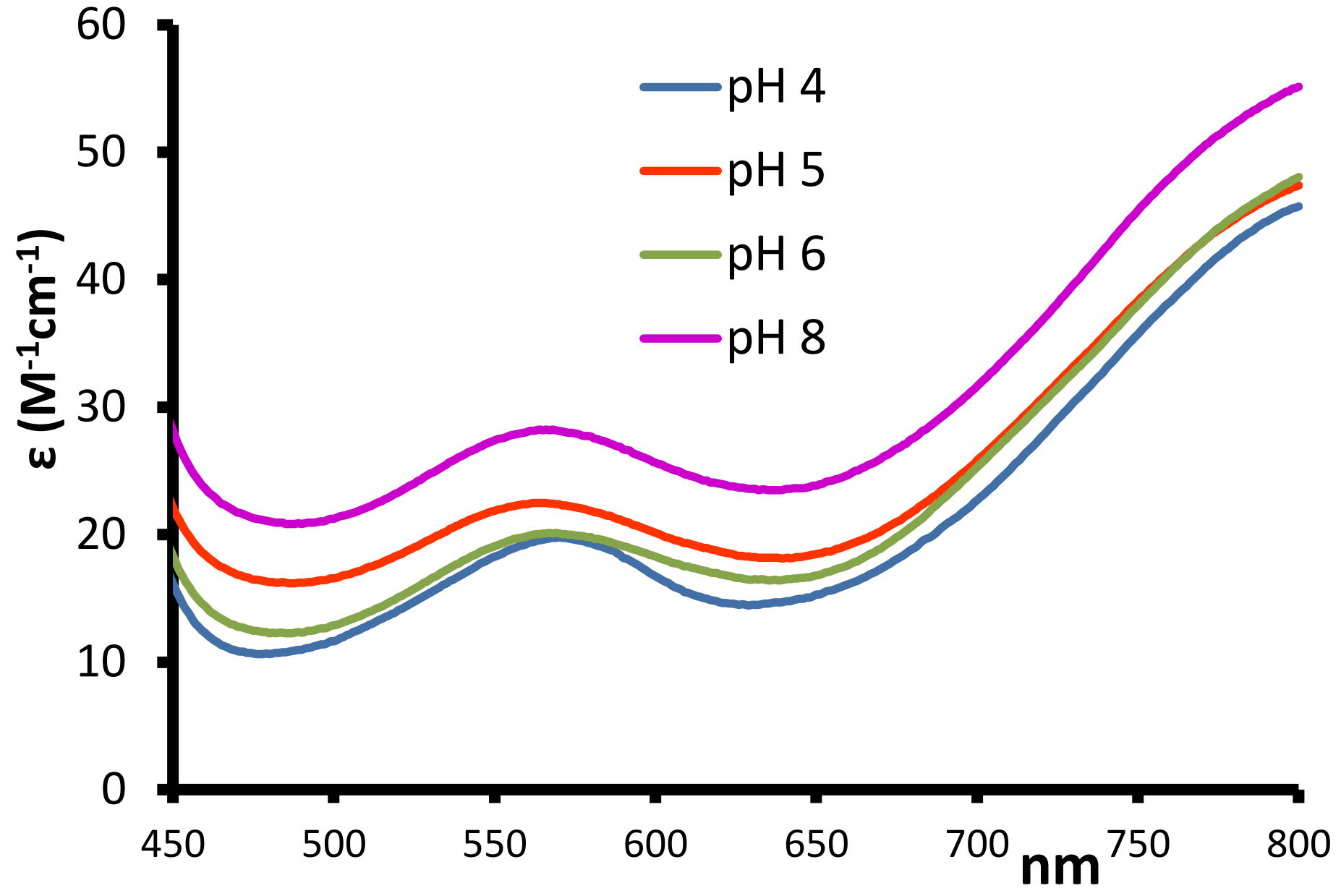

Figure S14. Visible absorption spectra of aqueous solutions (with 4.8% MeOH) containing $[V^{IV}O(acac)_2]$ at several pH values and $c_V = 2.0 \times 10^{-3}$ M.

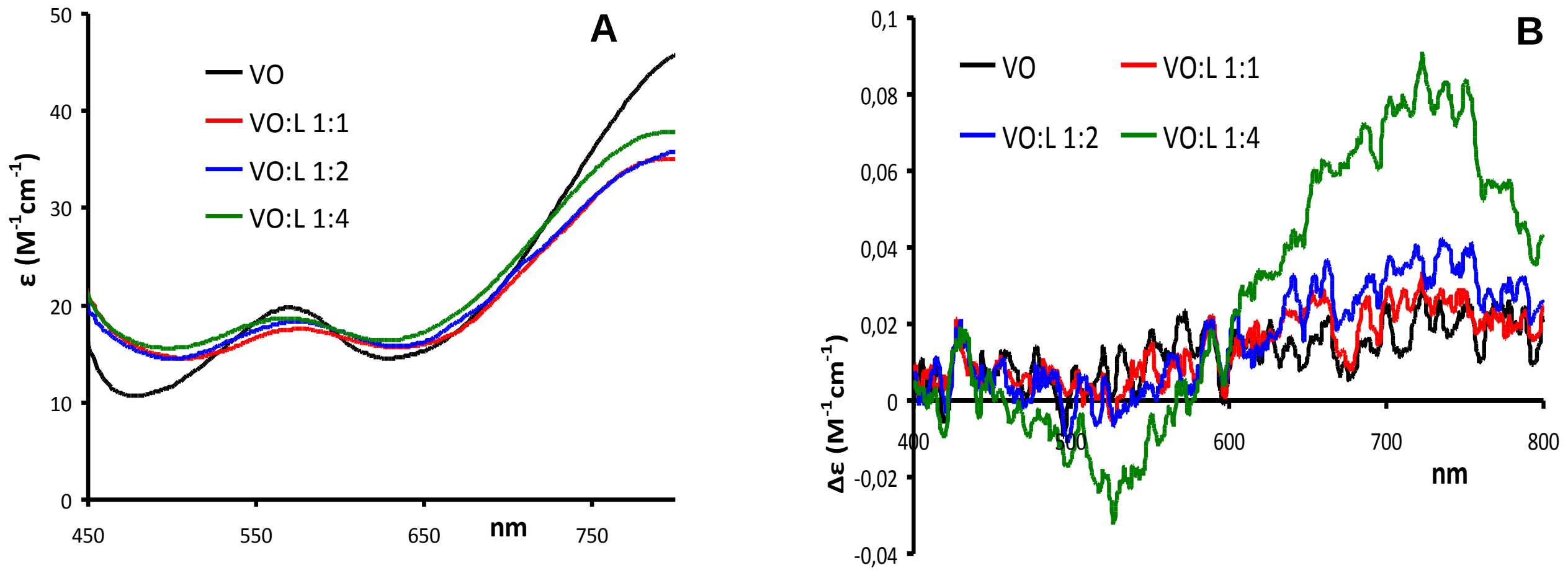

Figure S15. Spectra of solutions containing [$V^{IV}O(acac)_2$] and additions of His (L) at pH = 4.0 ± 0.3. The indicated VO:L ratios correspond to [$V^{IV}O(acac)_2$]:His molar ratios of independently prepared samples. (A) Visible absorption spectra; (B) circular dichroism spectra (note that the black spectrum corresponds to the baseline).

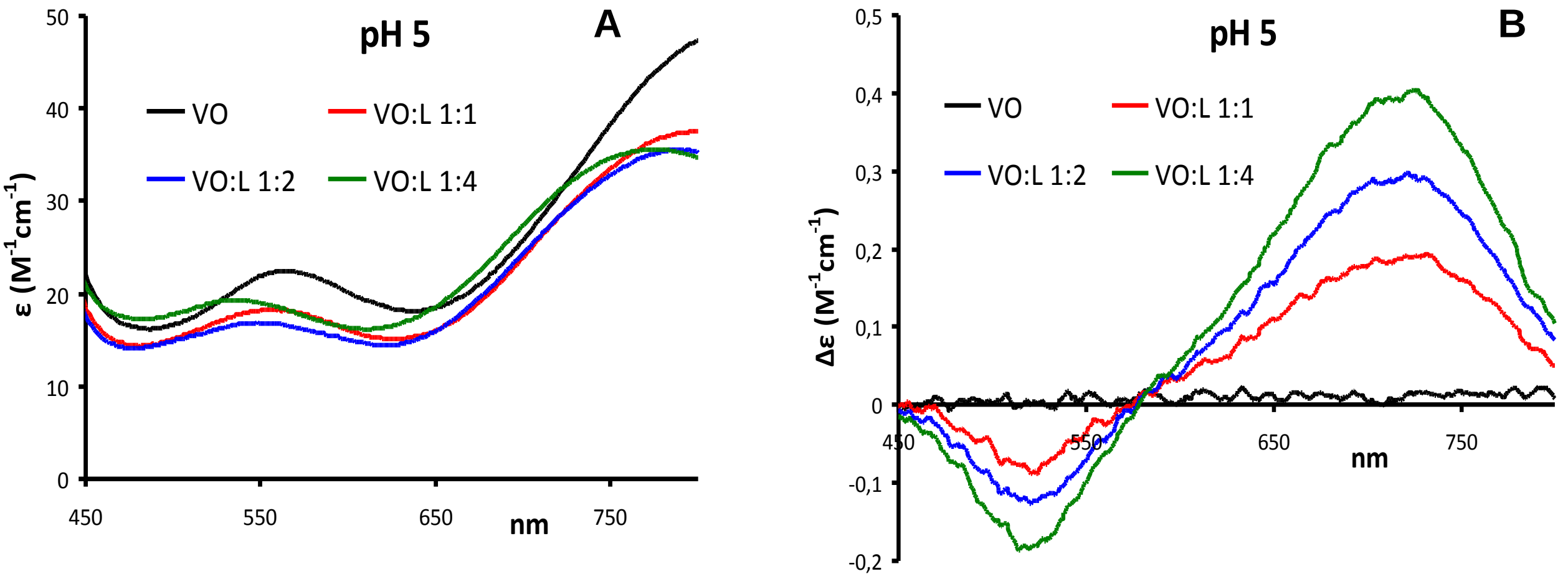

Figure S16. Spectra of solutions containing [$V^{IV}O(acac)_2$] and additions of His (L) at pH = 5.0 ± 0.3. The V^{IV} concentration is ~2.1 mM, and the indicated VO:L ratios correspond to [$V^{IV}O(acac)_2$]:His molar ratios of independently prepared samples. (A) Visible absorption spectra; (B) circular dichroism spectra (note that the black spectrum corresponds to the baseline).

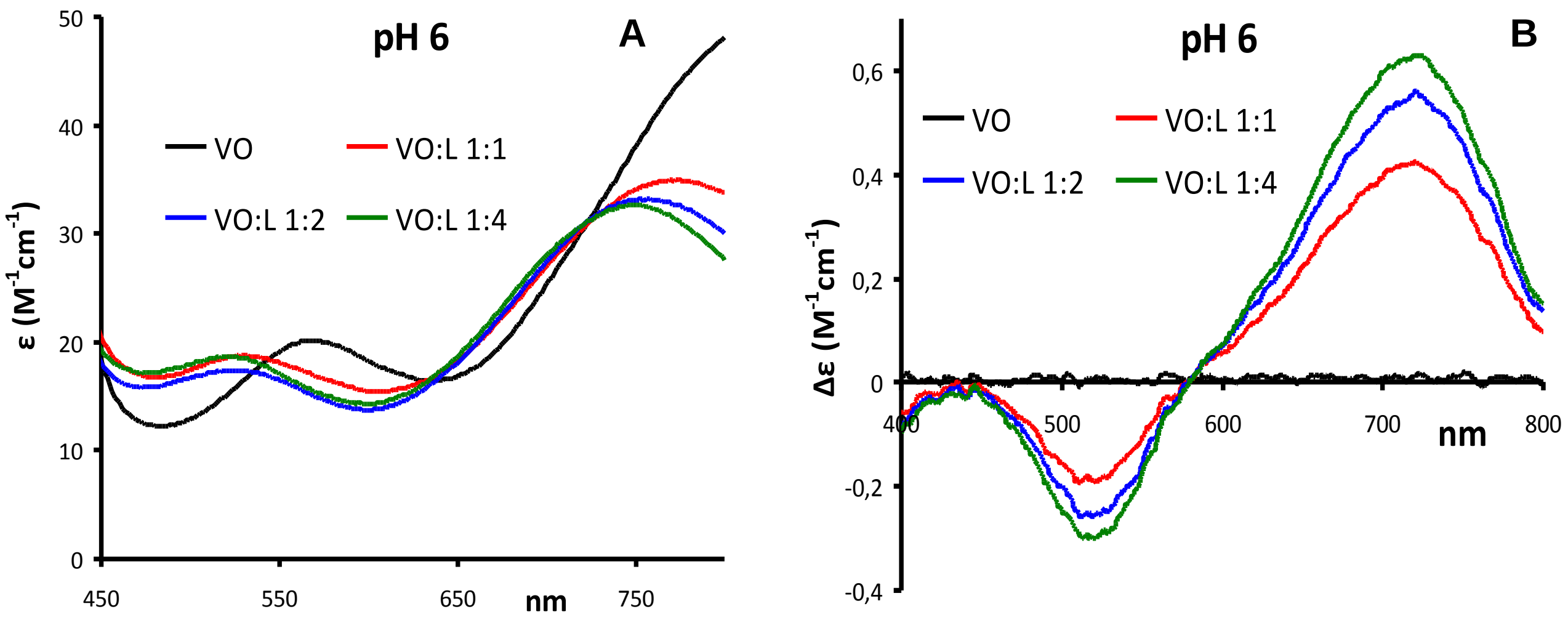

Figure S17. Spectra of solutions containing [$V^{IV}O(acac)_2$] and additions of His (L) at pH = 6.0 ± 0.3. The V^{IV} concentration is ~2.1 mM, and the indicated VO:L ratios correspond to [$V^{IV}O(acac)_2$]:His molar ratios of independently prepared samples. (A) Visible absorption spectra; (B) circular dichroism spectra (note that the black spectrum corresponds to the baseline).

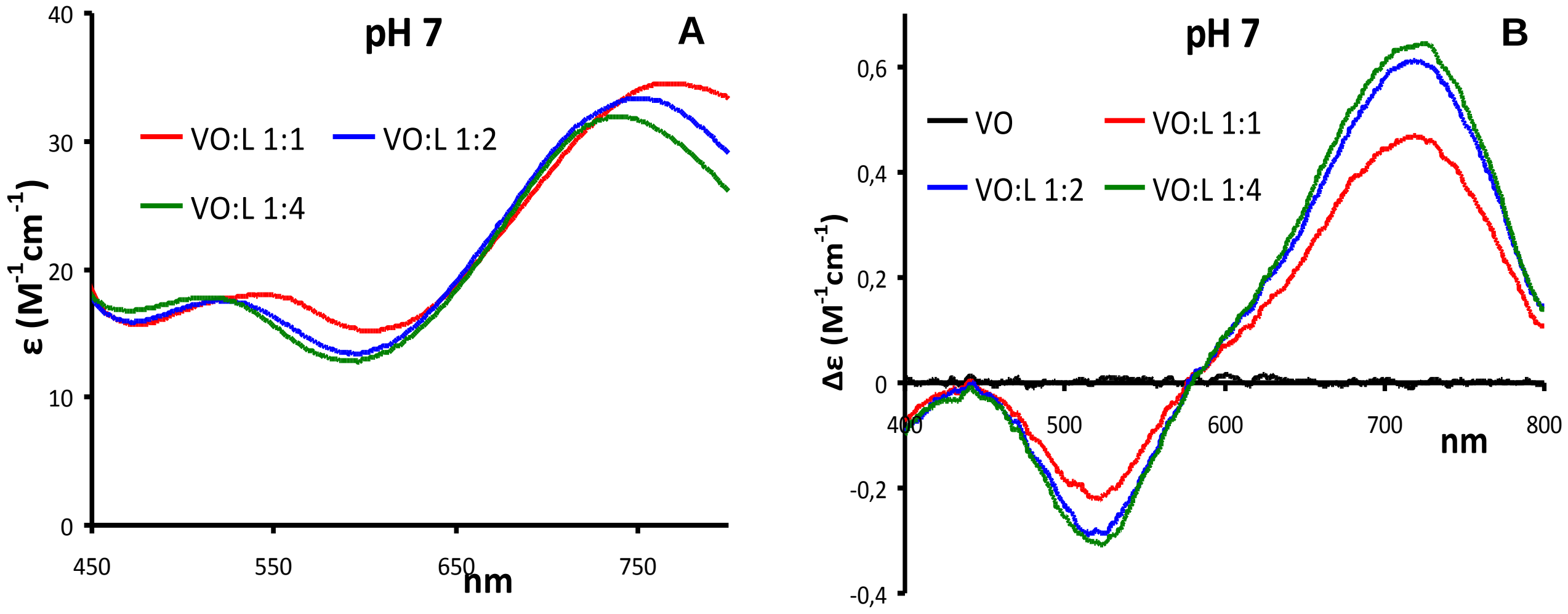

Figure S18. Spectra of solutions containing [$V^{IV}O(acac)_2$] and additions of His (L) at pH = 7.0 ± 0.1. The V^{IV} concentration is ~2.1 mM, and the indicated VO:L ratios correspond to [$V^{IV}O(acac)_2$]:His molar ratios of independently prepared samples. (A) Visible absorption spectra; (B) circular dichroism spectra (note that the black spectrum corresponds to the baseline).

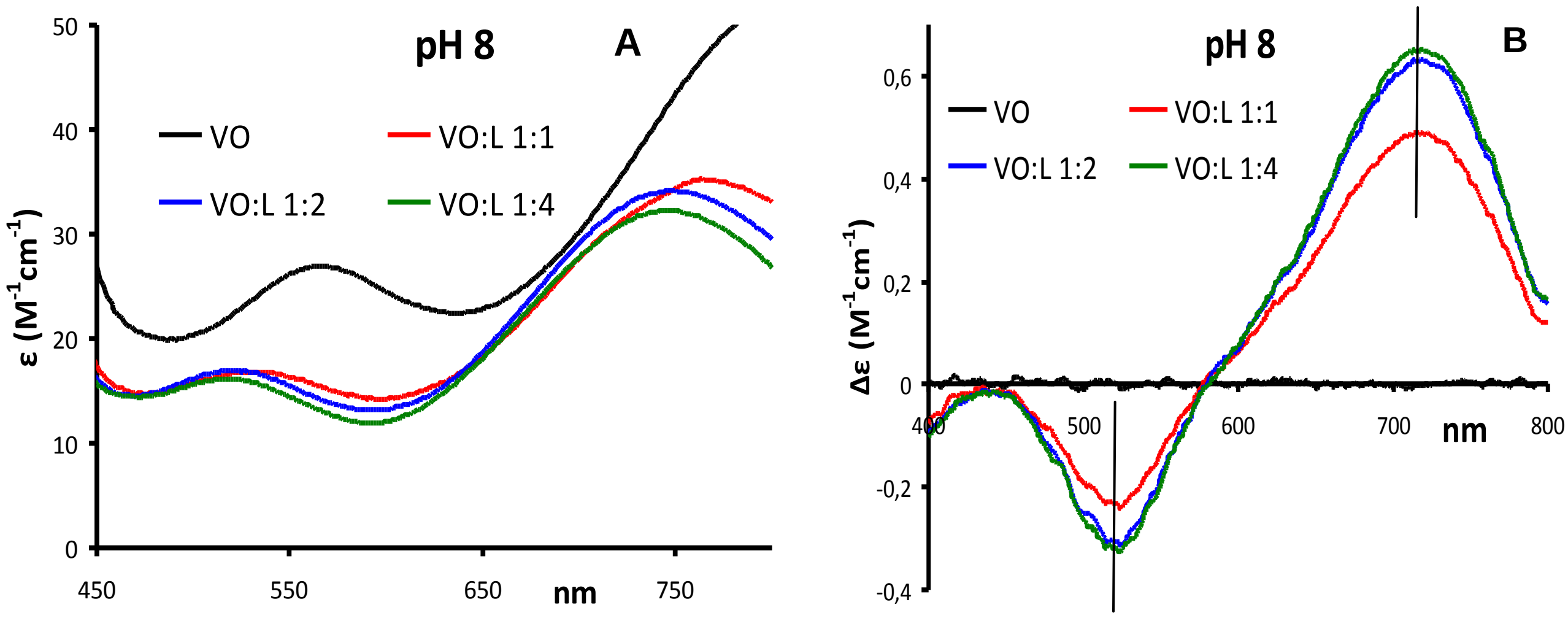

Figure S19. Spectra of solutions containing [$V^{IV}O(acac)_2$] and additions of His (L) at pH = 8.05 ± 0.15. The V^{IV} concentration is ~2.1 mM, and the indicated VO:L ratios correspond to [$V^{IV}O(acac)_2$]:His molar ratios of independently prepared samples. (A) Visible absorption spectra; (B) circular dichroism spectra (note that the black spectrum corresponds to the baseline).

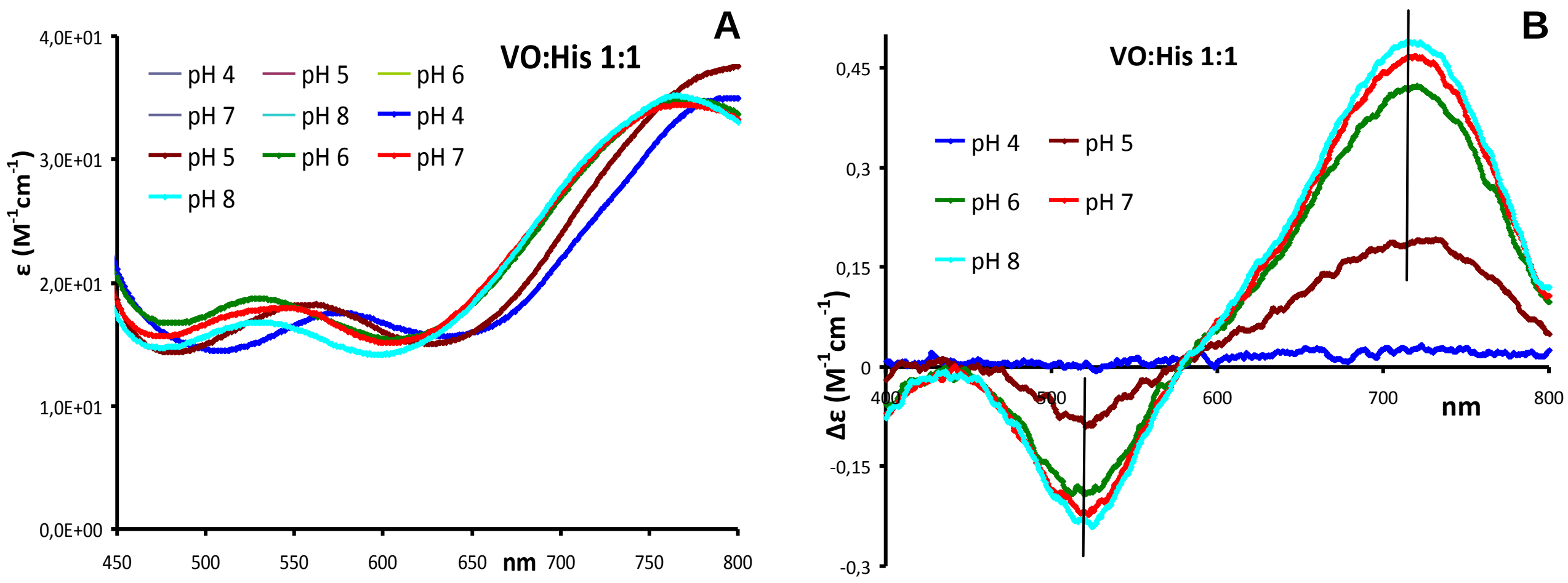

Figure S20. Spectra of solutions containing [$V^{IV}O(acac)_2$] and His (L) with a [$V^{IV}O(acac)_2$]:His molar ratio of 1:1 at several pH values. The V^{IV} concentration is ~2.1 mM, and the spectra correspond to those included in Figures S15-S19. (A) Visible absorption spectra; (B) circular dichroism spectra (the approximate positions of the λ_{max} of bands are indicated). Note that for CD spectra in the part B, the baseline (not shown) was subtracted from all the recorded spectra.

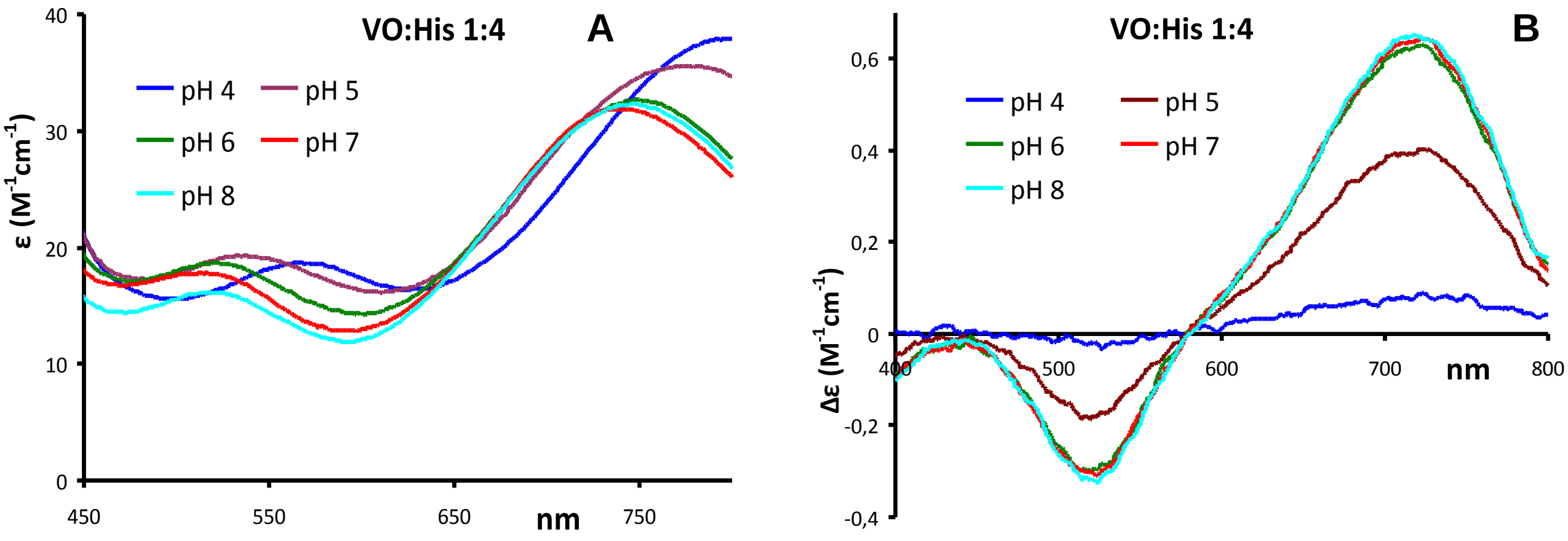

Figure S21. Spectra of solutions containing [$V^{IV}O(acac)_2$] and His (L) with a [$V^{IV}O(acac)_2$]:His molar ratio of 1:4 at several pH values. The V^{IV} concentration is ~2.1 mM, and the spectra correspond to those included in Figures S15-S19. (A) Visible absorption spectra; (B) circular dichroism spectra.

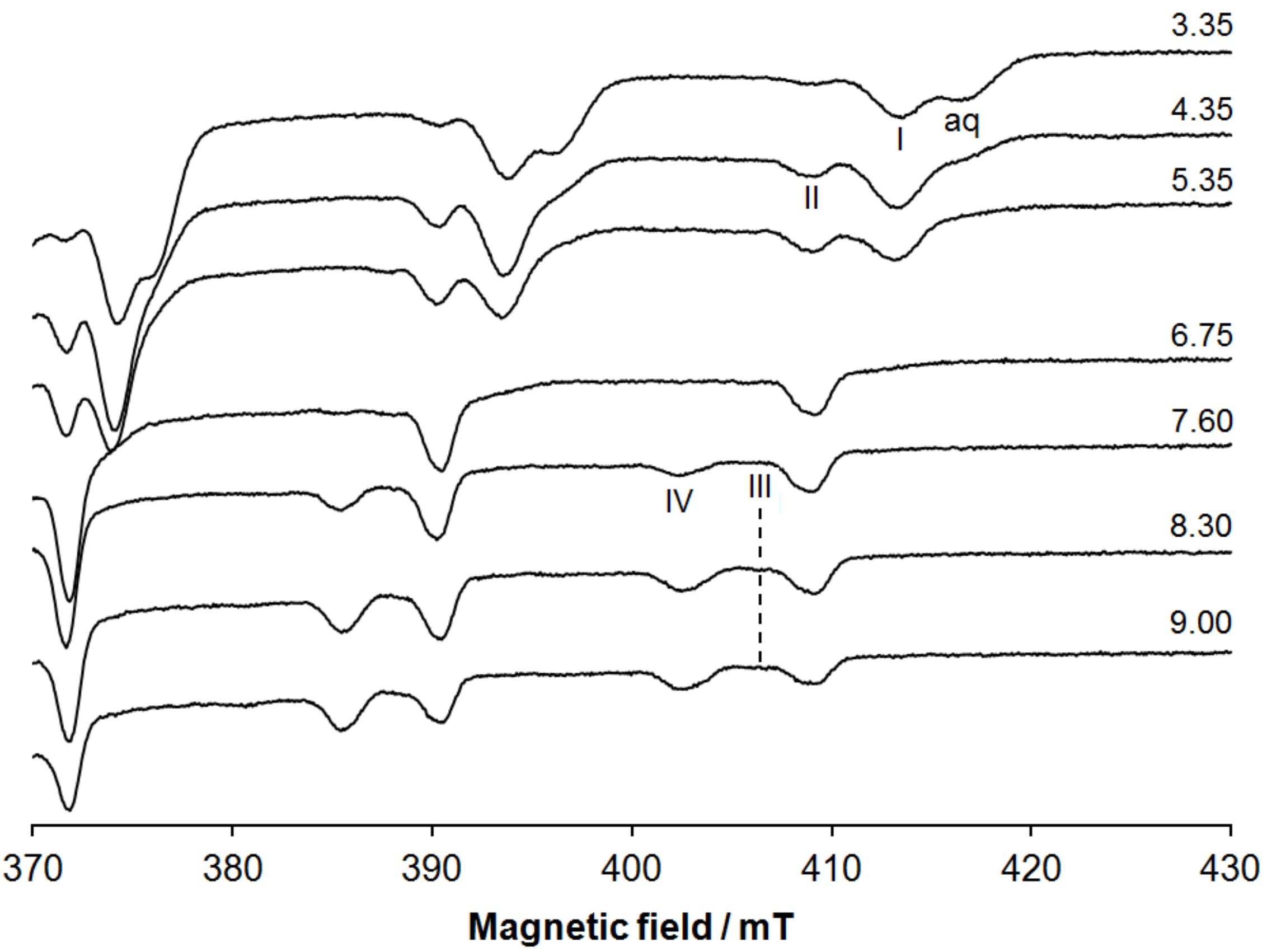

Figure S22. Anisotropic EPR spectra of solutions containing $V^{IV}O^{2+}$, acac and Cys with molar ratio 1:1:1 and $c_V = 2.0 \times 10^{-3}$ M at several pH values. The resonances of these species are indicated: **aq**, $[V^{IV}O(H_2O)_5]^{2+}$; **I**, $[V^{IV}O(acac)]^+$; **II**, $[V^{IV}O(acac)_2]$; **III**, $[V^{IV}O(acac)(Cys)]^-$; **IV**, $[V^{IV}O(Cys)_2]^{2-}$. The resonances of the mixed-ligand species $[V^{IV}O(acac)(Cys)]^-$ are also denoted with the dotted line.

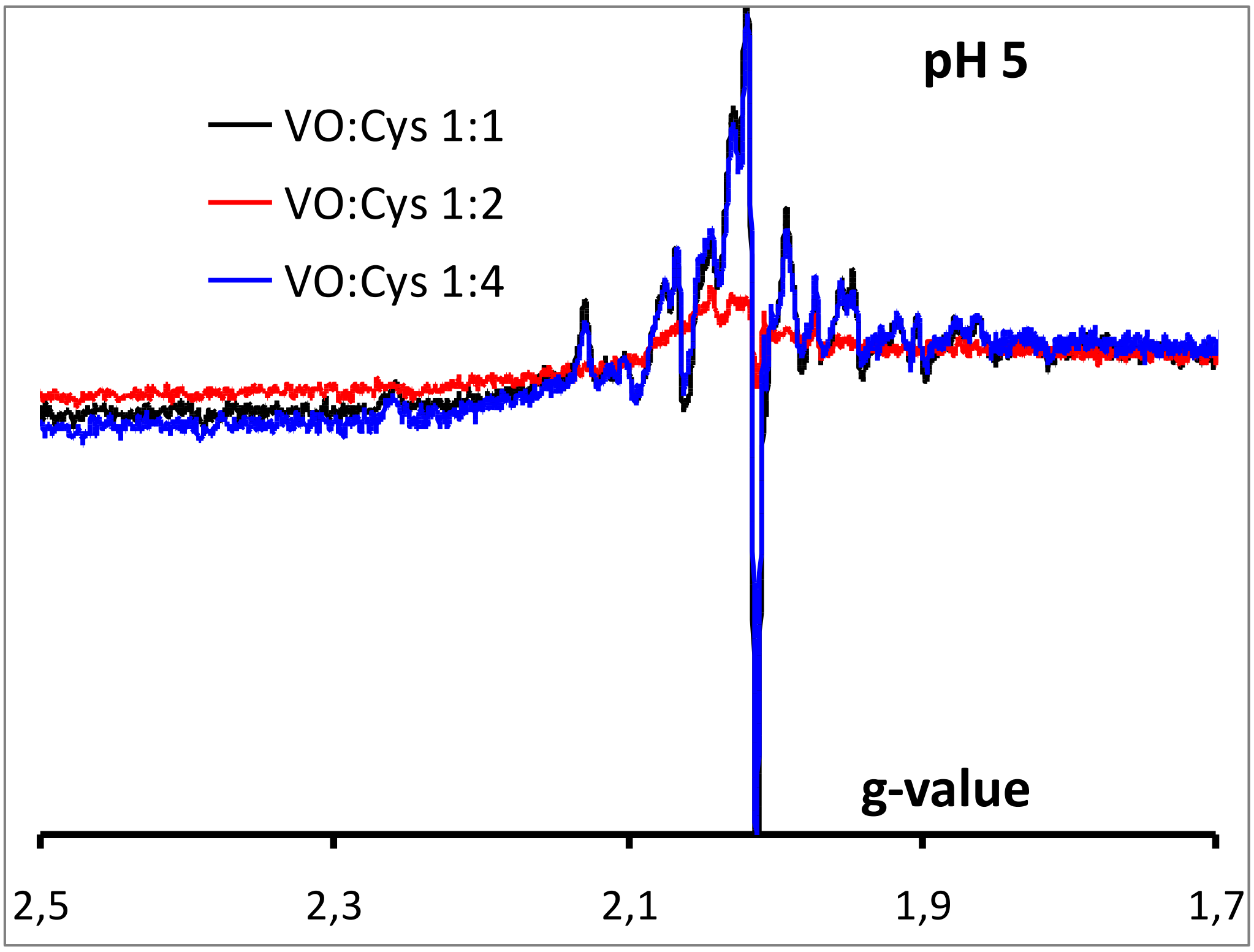

Figure S23. EPR spectra of solutions containing [$V^{IV}O(acac)_2$] and Cys at pH = 5.00 ± 0.12 varying the molar ratio with c_V ~2.0 mM.

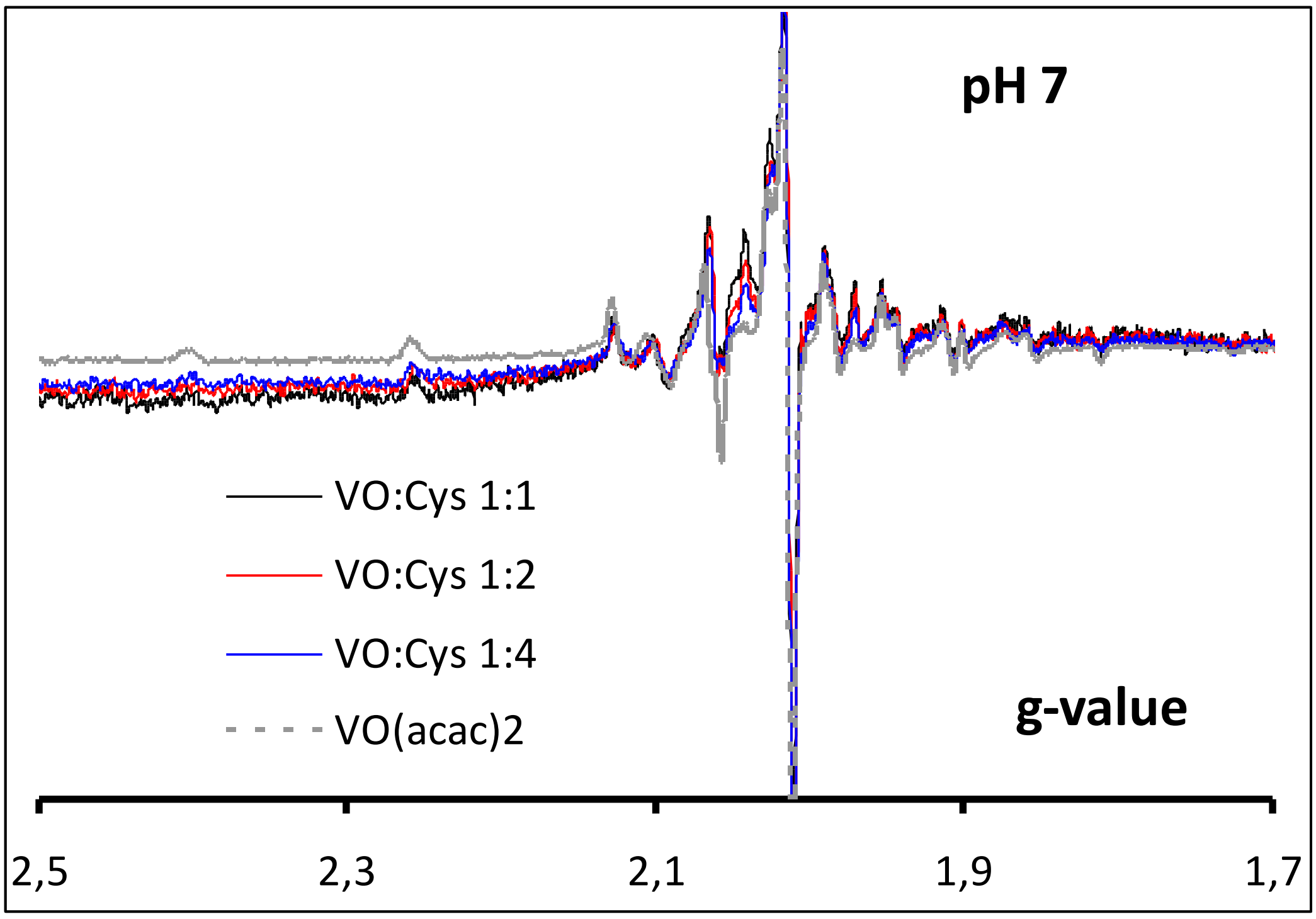

Figure S24. EPR spectra of solutions containing [$V^{IV}O(acac)_2$] (VO) and Cys at pH = 7.00 ± 0.09 varying the [$V^{IV}O(acac)_2$]:Cys molar ratio with c_V ~2.0 mM.

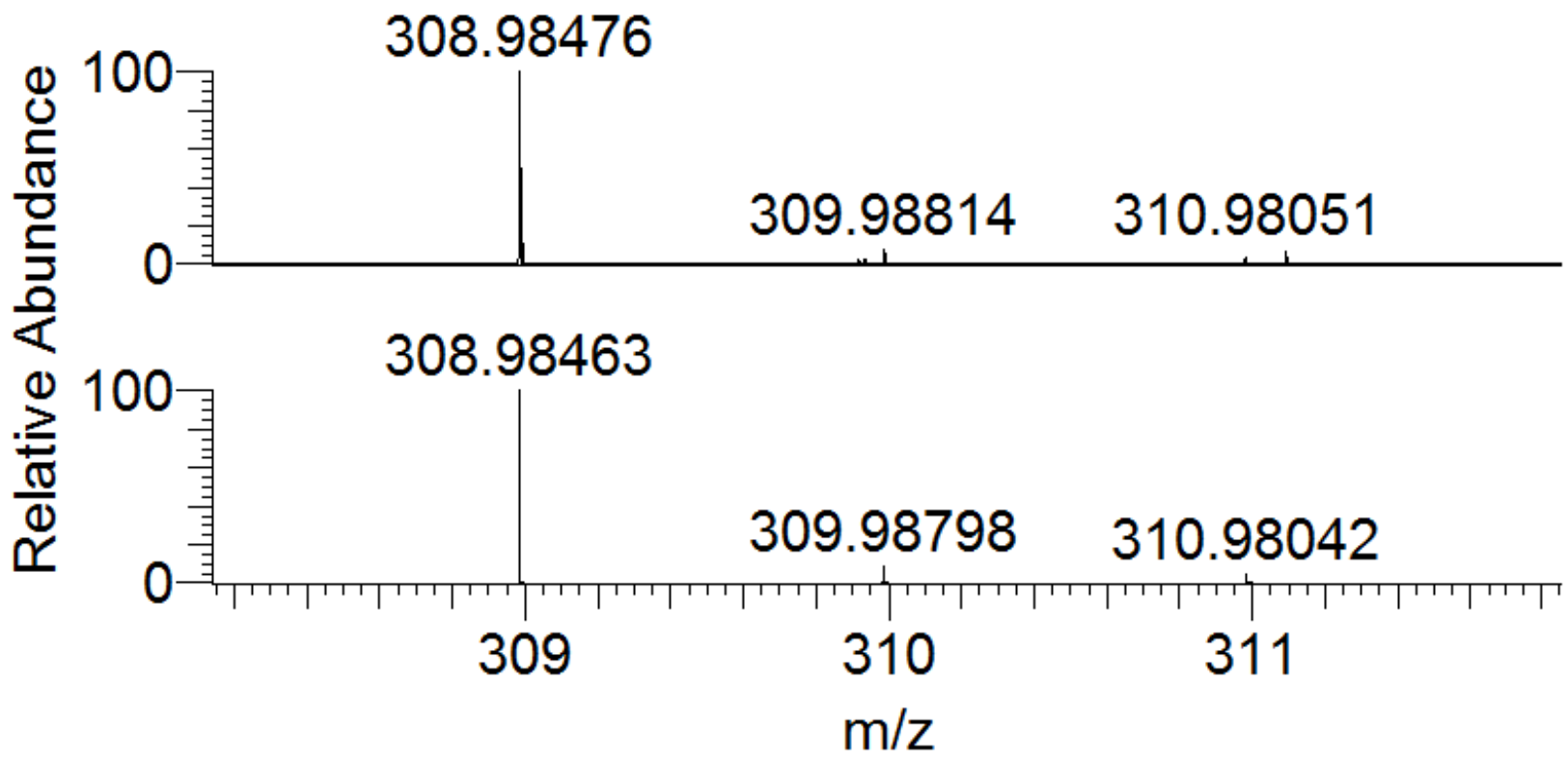

Figure S25. Experimental (above) and calculated (below) isotopic pattern for the peak of $[V^{IV}O(acac)(Cys)+H+Na]^+$ revealed at m/z = 308.9848 in the positive ESI-MS(+) spectrum, recorded on the $V^{IV}O^{2+}$/acac/Cys system with molar ratio 1:1:1 (ultrapure LC-MS water, pH = 7.50, $c_V = 5.0 \times 10^{-6}$ M).

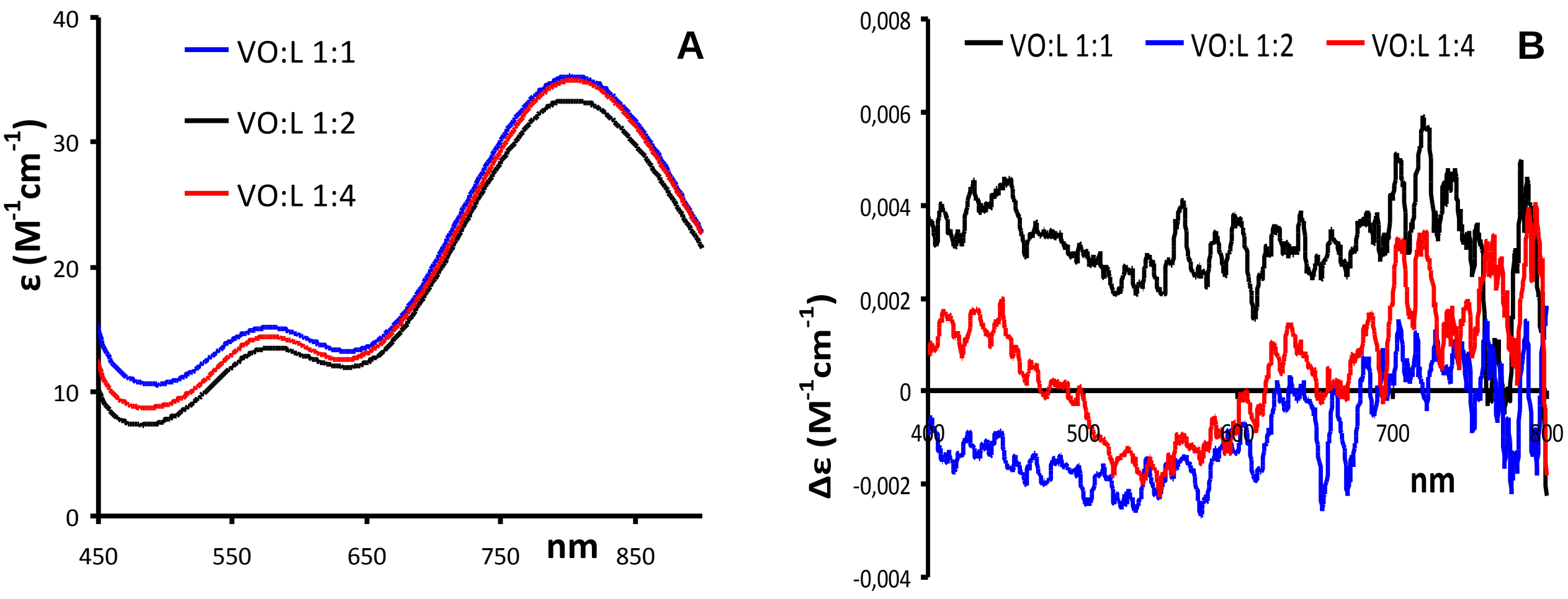

Figure S26. Spectra of solutions containing $[V^{IV}O(acac)_2]$ and additions of Cys (L) at pH = 4.06 ± 0.02 and c_V ~2.0 mM. The VO:L ratios indicated correspond to $[V^{IV}O(acac)_2]$:Cys molar ratios. (A) Absorption spectra in the range 450-900 nm; (B) circular dichroism spectra in the range 400-800 nm. Note that the Δε values are extremely low, therefore the signal to noise ratio in the CD spectra does not allow stating if there is any genuine CD band being measured.

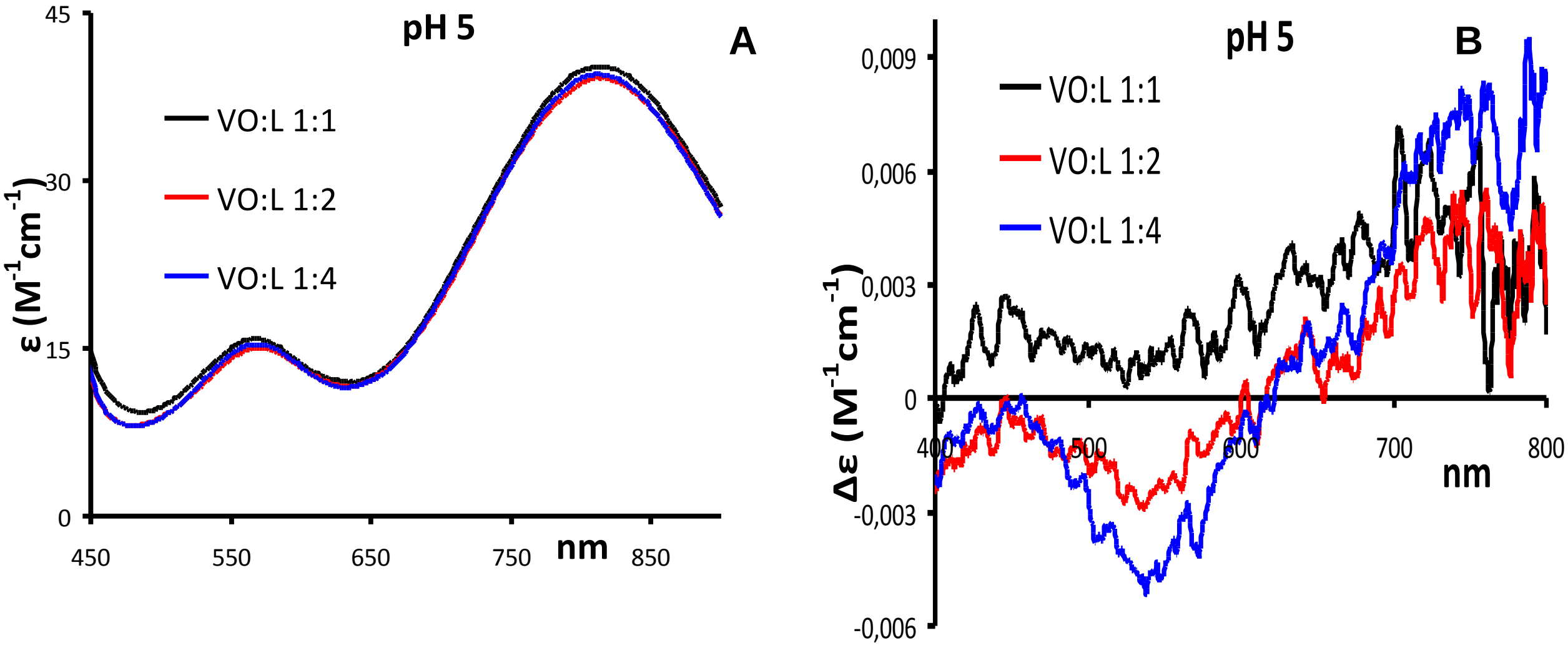

Figure S27. Spectra of solutions containing $[V^{IV}O(acac)_2]$ and additions of Cys (L) at pH = 5.08 ± 0.05 and c_V ~2.0 mM. The VO:L ratios indicated correspond to $[V^{IV}O(acac)_2]$:Cys molar ratios. (A) Absorption spectra in the range 450-900 nm; (B) circular dichroism spectra in the range 400-800 nm. Note that the Δε values are quite low, but demonstrate the formation of low amounts of species with Cys bound to $V^{IV}O^{2+}$.

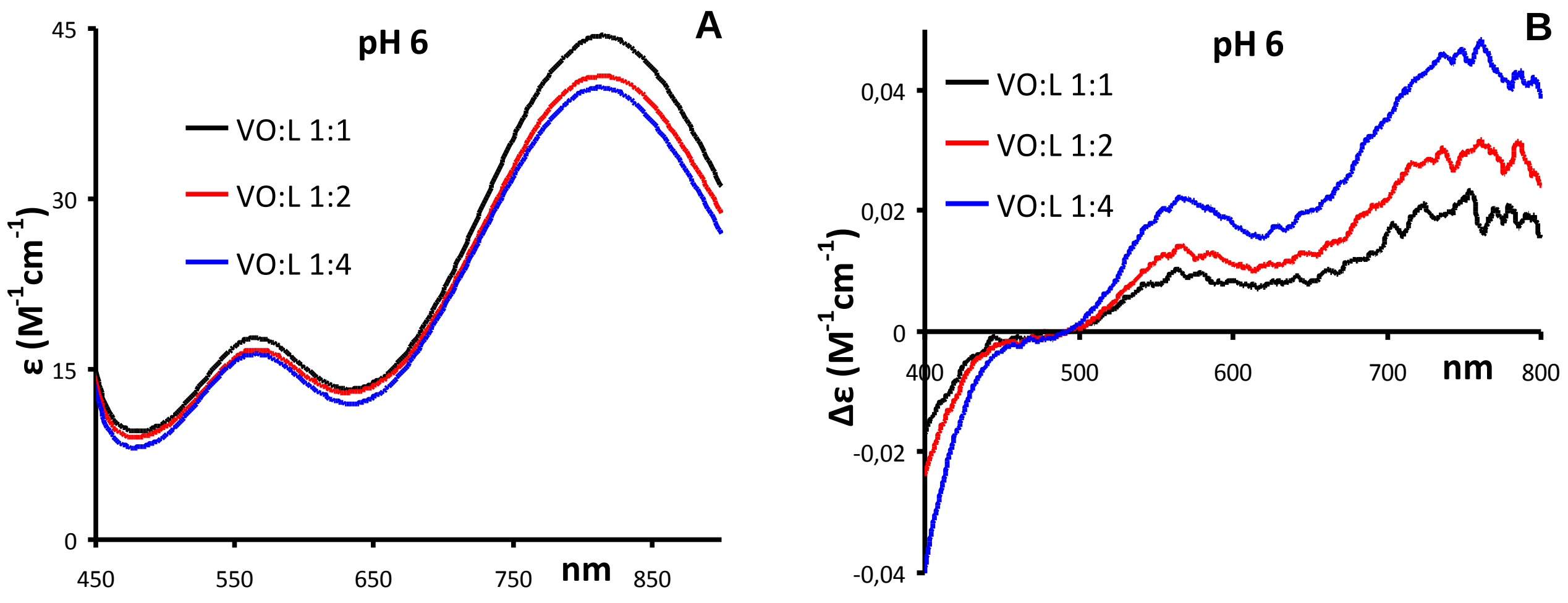

Figure S28. Spectra of solutions containing [$V^{IV}O(acac)_2$] and additions of Cys (L) at pH = 6.09 ± 0.02 and c_V ~2.0 mM. The VO:L ratios indicated correspond to [$V^{IV}O(acac)_2$]:Cys molar ratios. (A) Absorption spectra in the range 450-900 nm; (B) circular dichroism spectra in the range 400-800 nm. Note that the Δε values are quite low but demonstrate the formation of low amounts of species with Cys bound to $V^{IV}O^{2+}$.

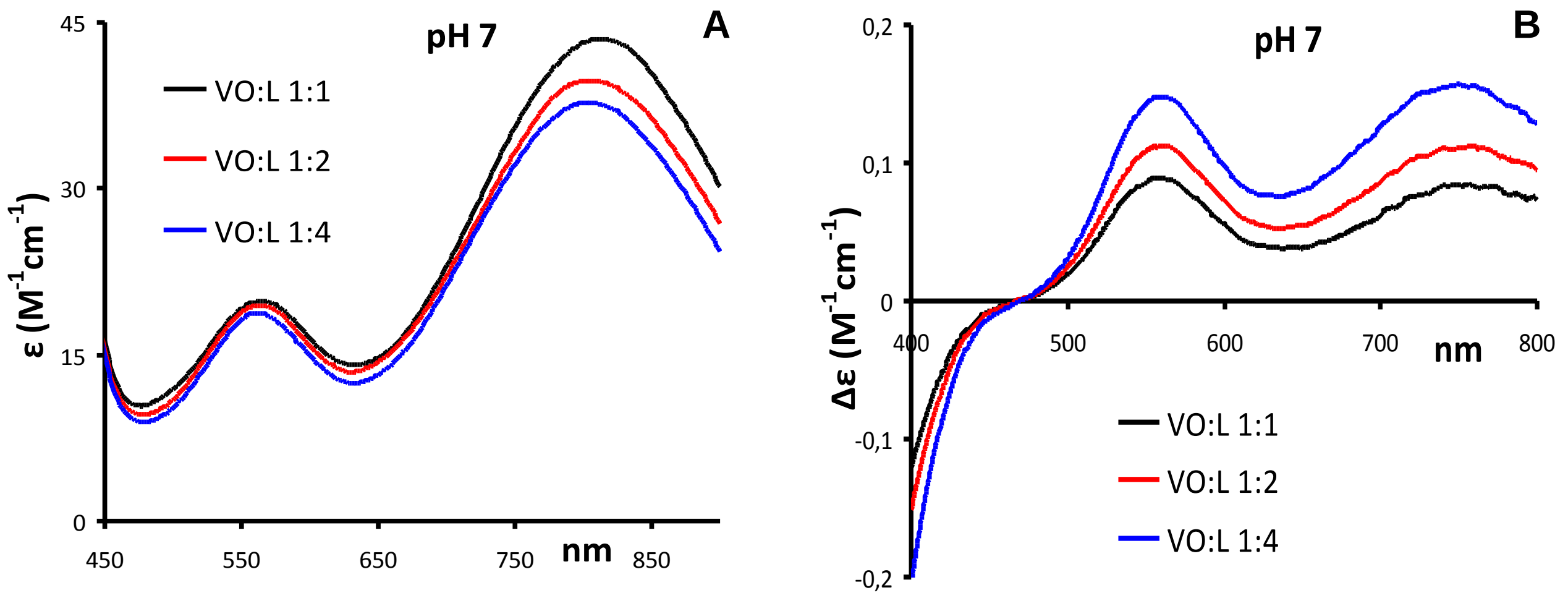

Figure S29. Spectra of solutions containing [$V^{IV}O(acac)_2$] and additions of Cys (L) at pH = 7.08 ± 0.02 and c_V ~2.0 mM. The VO:L ratios indicated correspond to [$V^{IV}O(acac)_2$]:Cys molar ratios. (A) Absorption spectra in the range 450-900 nm; (B) circular dichroism spectra in the range 400-800 nm. Note that the |Δε| values are much higher than those measured at pH = 6.0 and that the three spectra all cross Δε = 0 at λ ~470 nm.

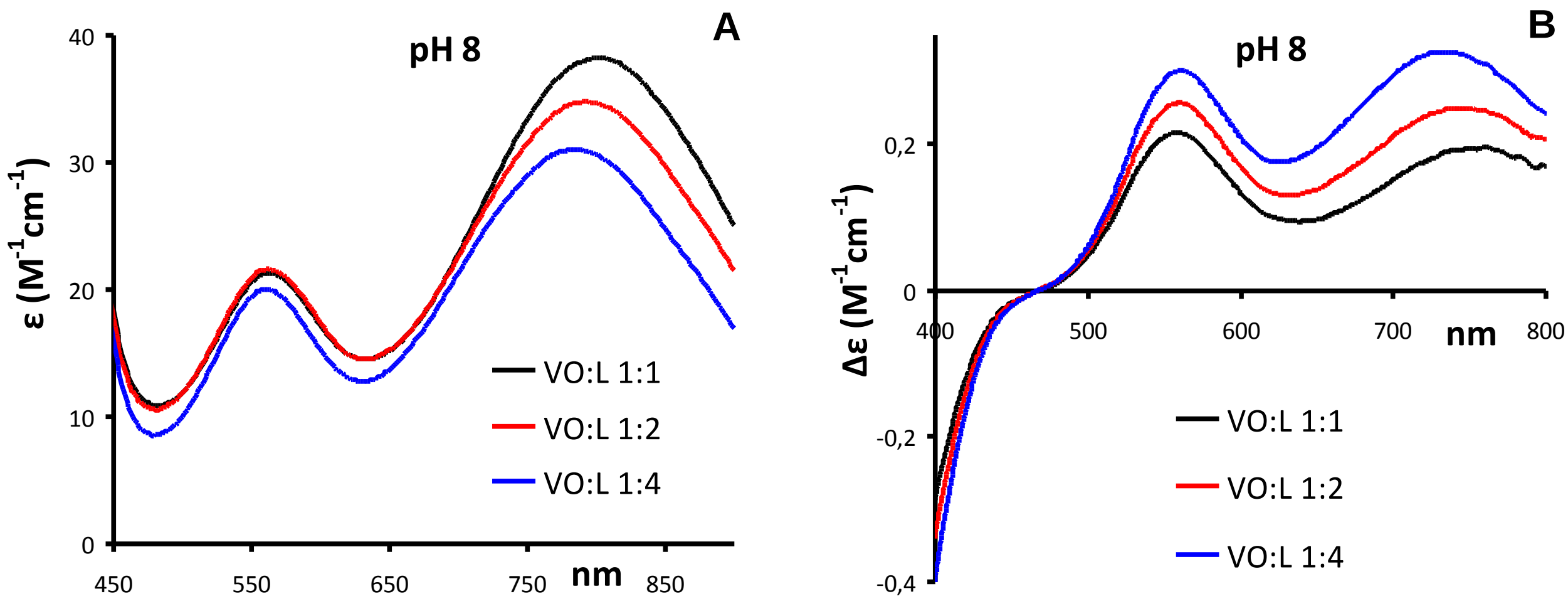

Figure S30. Spectra of solutions containing [$V^{IV}O(acac)_2$] and additions of Cys (L) at pH = 8.05 ± 0.05 and c_V ~2.0 mM. The VO:L ratios indicated correspond to [$V^{IV}O(acac)_2$]:Cys molar ratios. (A) Absorption spectra in the range 450-900 nm; (B) circular dichroism spectra in the range 400-800 nm. Note that the |Δε| values are higher than those measured at pH = 7.0 and that the three spectra all cross Δε = 0 at λ ~470 nm.

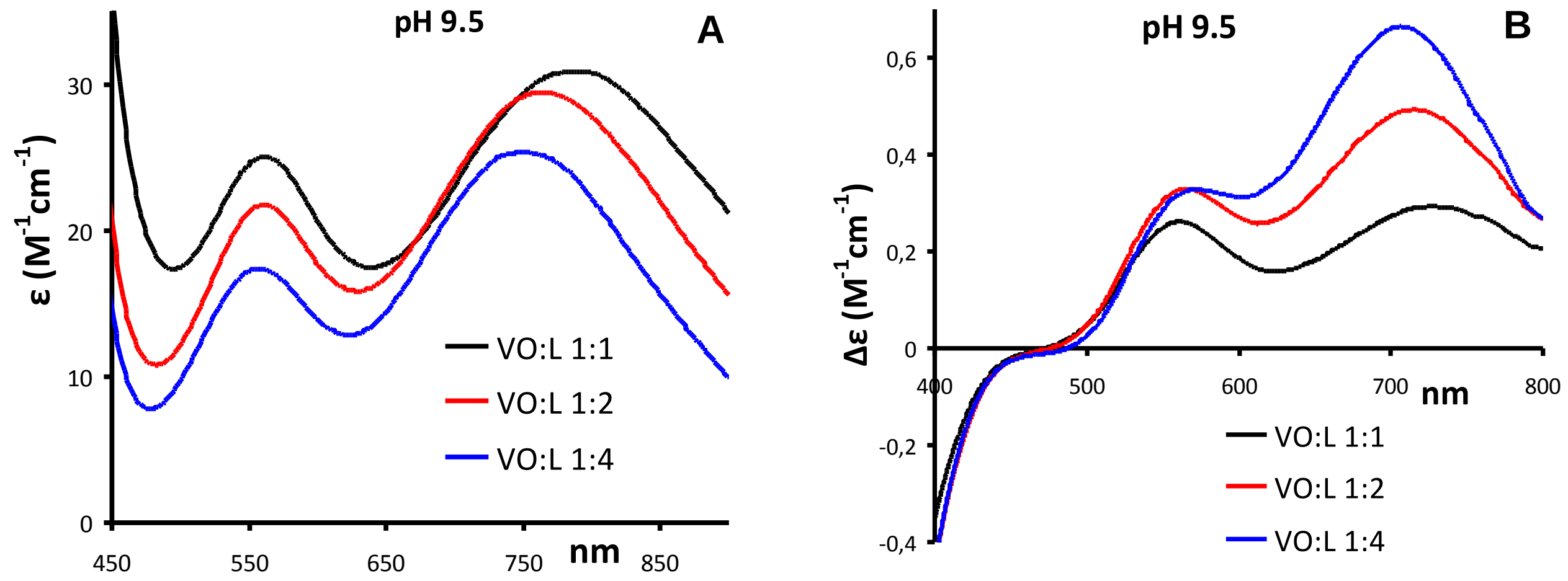

Figure S31. Spectra of solutions containing [$V^{IV}O(acac)_2$] and additions of Cys (L) at pH = 9.5 ± 0.15 and c_V ~2.0 mM. The VO:L ratios indicated correspond to [$V^{IV}O(acac)_2$]:Cys molar ratios. (A) Absorption spectra in the range 450-900 nm; (B) circular dichroism spectra in the range 400-800 nm. Note that the pattern of the spectra and the |Δε| values, particularly for λ > 600 nm, are higher than those measured at pH = 8.0. This means that distinct chiral $V^{IV}O$-complexes are being formed as Cys is added.

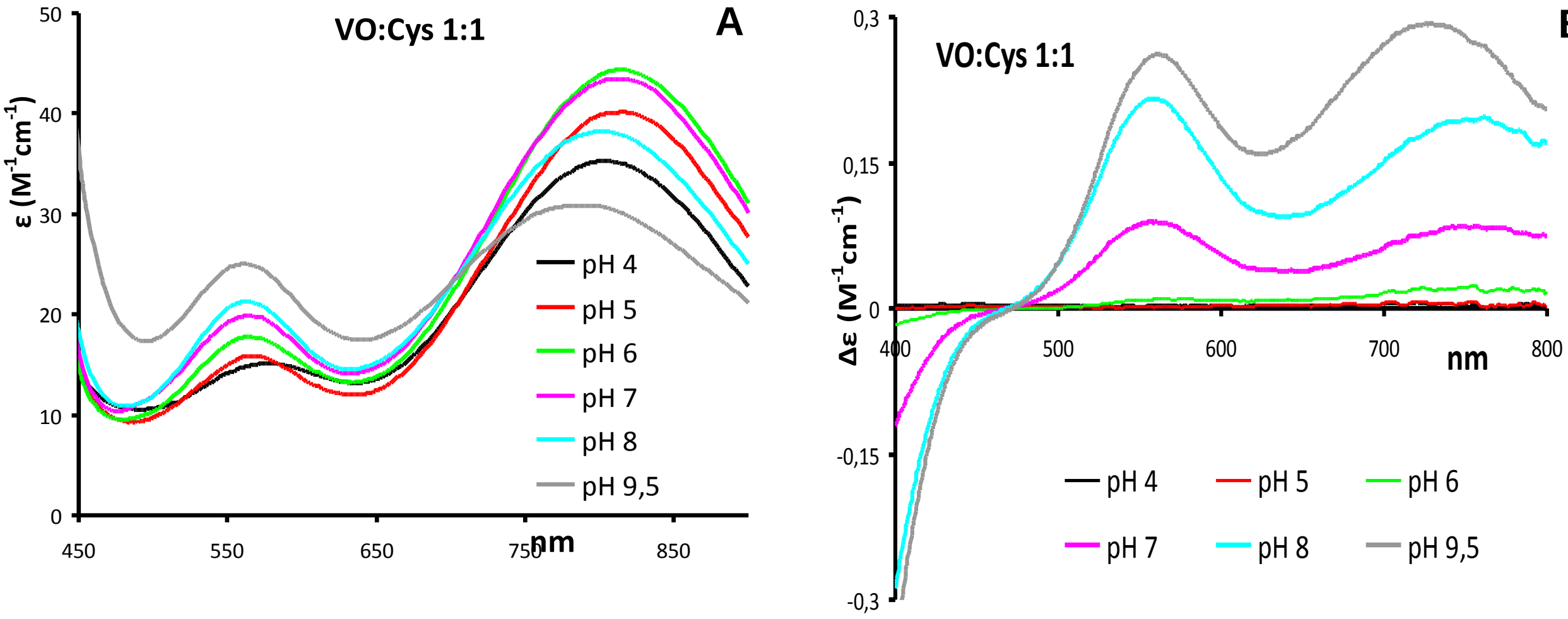

Figure S32. Spectra of solutions containing [$V^{IV}O(acac)_2$] and Cys (L) with a 1:1 [$V^{IV}O(acac)_2$]:Cys molar ratio and c_V ~2.0 mM at several pH values. These spectra correspond to those included in Figures S26-S31. (A) Absorption spectra in the range 450-900 nm; (B) circular dichroism spectra in the range 400-800 nm.

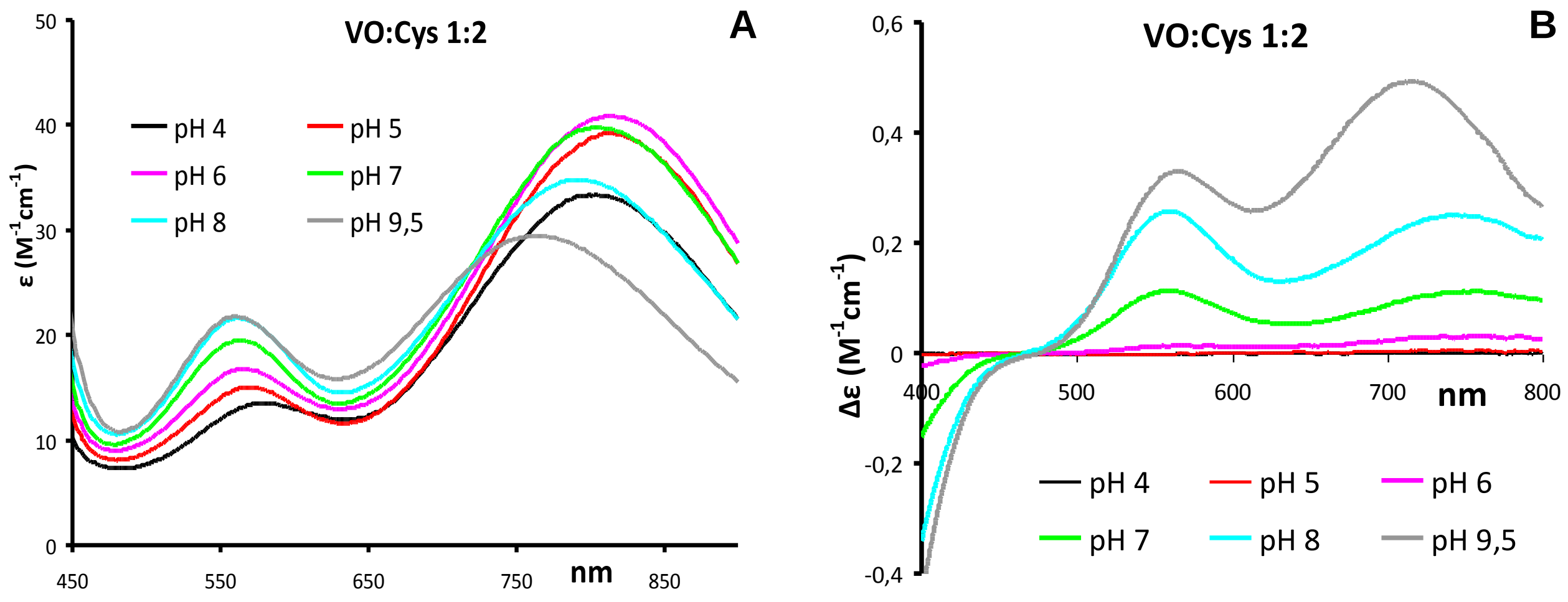

Figure S33. Spectra of solutions containing [$V^{IV}O(acac)_2$] and Cys (L) with a 1:2 [$V^{IV}O(acac)_2$]:Cys molar ratio and c_V ~2.0 mM at several pH values. These spectra correspond to those included in Figures S26-S31. (A) Absorption spectra in the range 450-900 nm; (B) circular dichroism spectra in the range 400-800 nm.

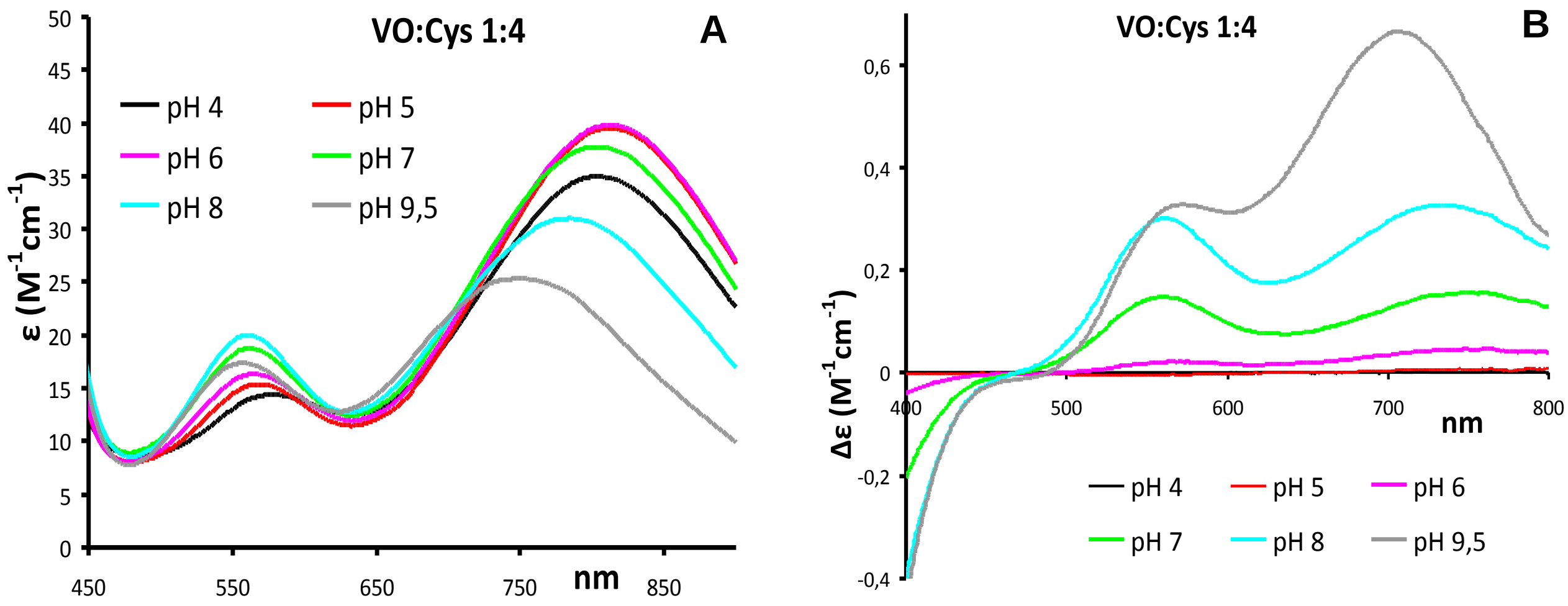

Figure S34. Spectra of solutions containing [$V^{IV}O(acac)_2$] and Cys (L) with a 1:4 [$V^{IV}O(acac)_2$]:Cys molar ratio and c_V ~2.0 mM at several pH values. These spectra correspond to those included in Figures S26-S31. (A) Absorption spectra in the range 450-900 nm; (B) circular dichroism spectra in the range 400-800 nm.

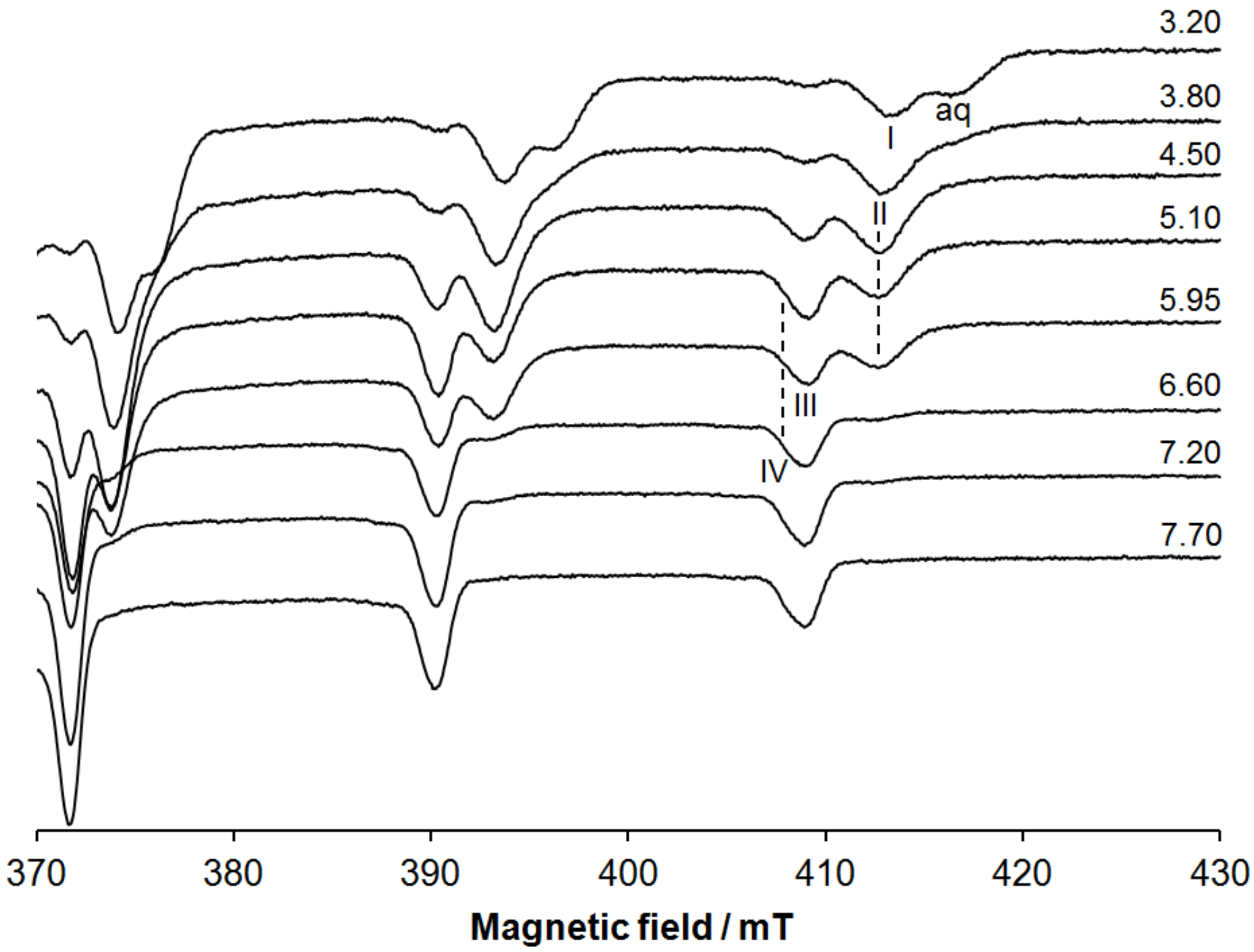

Figure S35. Anisotropic EPR spectra of solutions containing $V^{IV}O^{2+}$, acac and Asp with molar ratio 1:1:1 and $c_V = 2.0 \times 10^{-3}$ M at various pH values. The resonances of these species are indicated: **aq**, $[V^{IV}O(H_2O)_5]^{2+}$; **I**, $[V^{IV}O(acac)]^{+}$; **II**, $[V^{IV}O(acac)(HAsp)]$ / $[V^{IV}O(acac)(Asp)]^{-}$, isomer with (COO^{-}, COO^{-}, NH_2^{ax}) binding mode; **III**, $[V^{IV}O(acac)_2]$; **IV**, $[V^{IV}O(acac)(Asp)]^{-}$, isomer with (NH_2, COO^{-}, COO^{-ax}) coordination. The resonances of the mixed-ligand species $[V^{IV}O(acac)(HAsp)]$ / $[V^{IV}O(acac)(Asp)]^{-}$ are also denoted with the dotted lines.

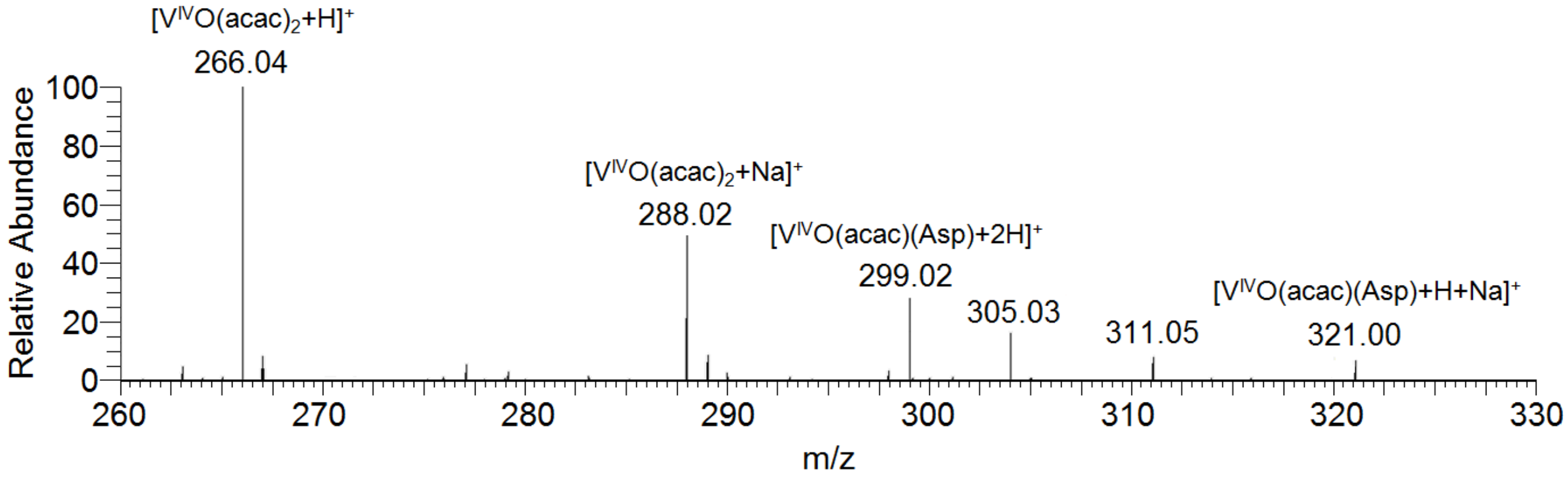

Figure S36. ESI-MS(+) spectrum of the $V^{IV}O^{2+}$/acac/Asp system with molar ratio 1:1:1 (ultrapure LC-MS water, pH = 4.85, $c_V = 5.0 \times 10^{-6}$ M). Only two decimal numbers are reported for clarity.

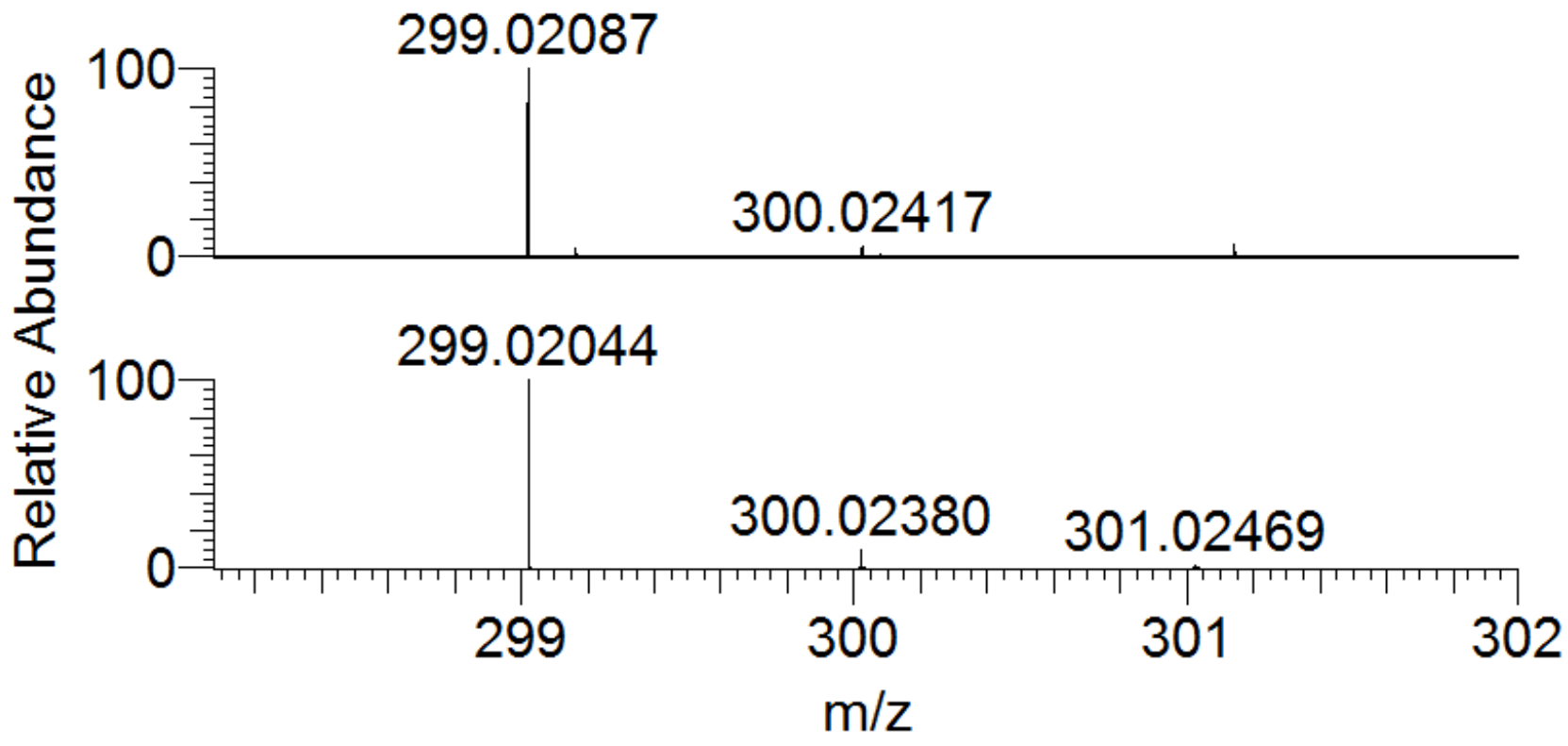

Figure S37. Experimental (above) and calculated (below) isotopic pattern for the peak of $[V^{IV}O(acac)(Asp)+2H]^+$ revealed at m/z = 299.0209 in the ESI-MS(+) spectrum recorded on the $V^{IV}O^{2+}$/acac/Asp system with molar ratio 1:1:1 (ultrapure LC-MS water, pH = 4.85, $c_V = 5.0 \times 10^{-6}$ M).

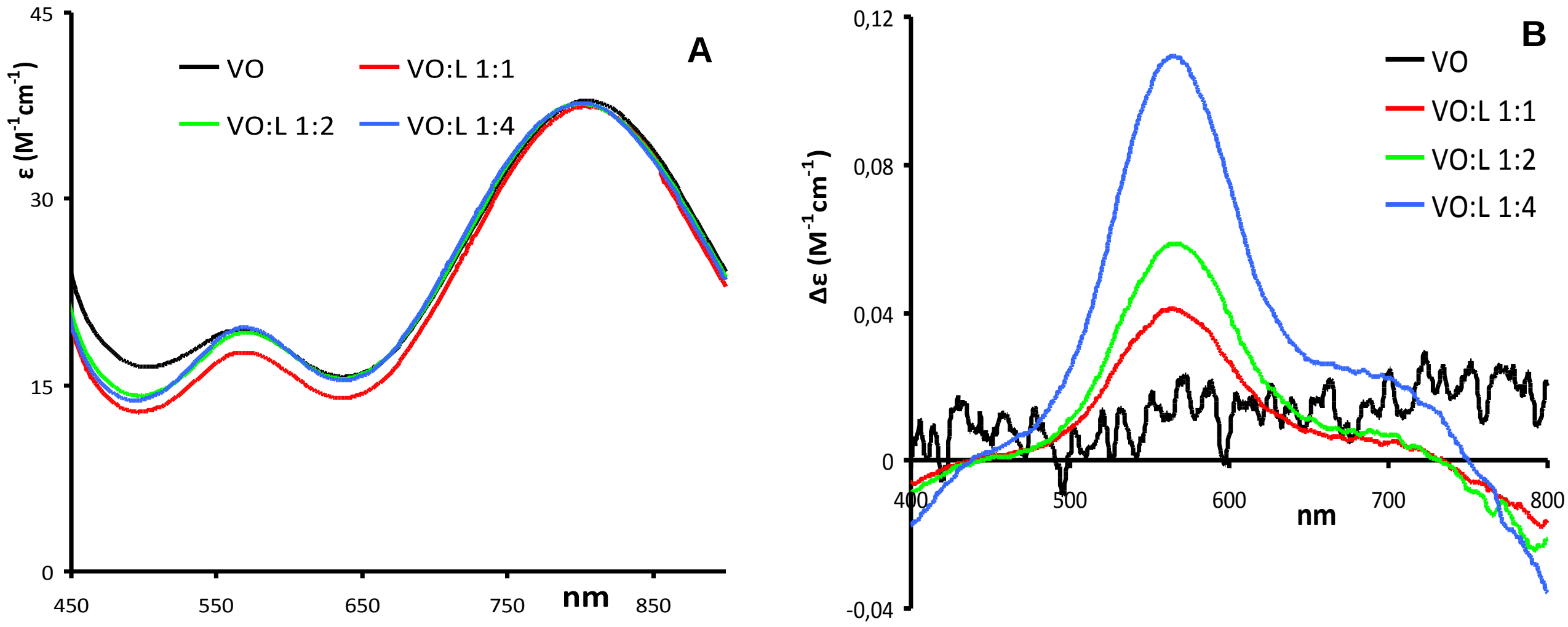

Figure S38. Spectra of solutions containing [$V^{IV}O(acac)_2$] and additions of Asp (L) at pH = 3.92 ± 0.06 and c_V ~2.0 mM. The indicated VO:L ratios correspond to [$V^{IV}O(acac)_2$]:Asp molar ratios. (A) Absorption spectra in the range 450-900 nm; (B) circular dichroism spectra in the range 400-800 nm. The Δε values are relatively low but demonstrate the formation of species with Asp bound to oxidovanadium(IV). Note that the black spectrum was recorded with a solution of [$V^{IV}O(acac)_2$], where no CD bands are expected to exist.

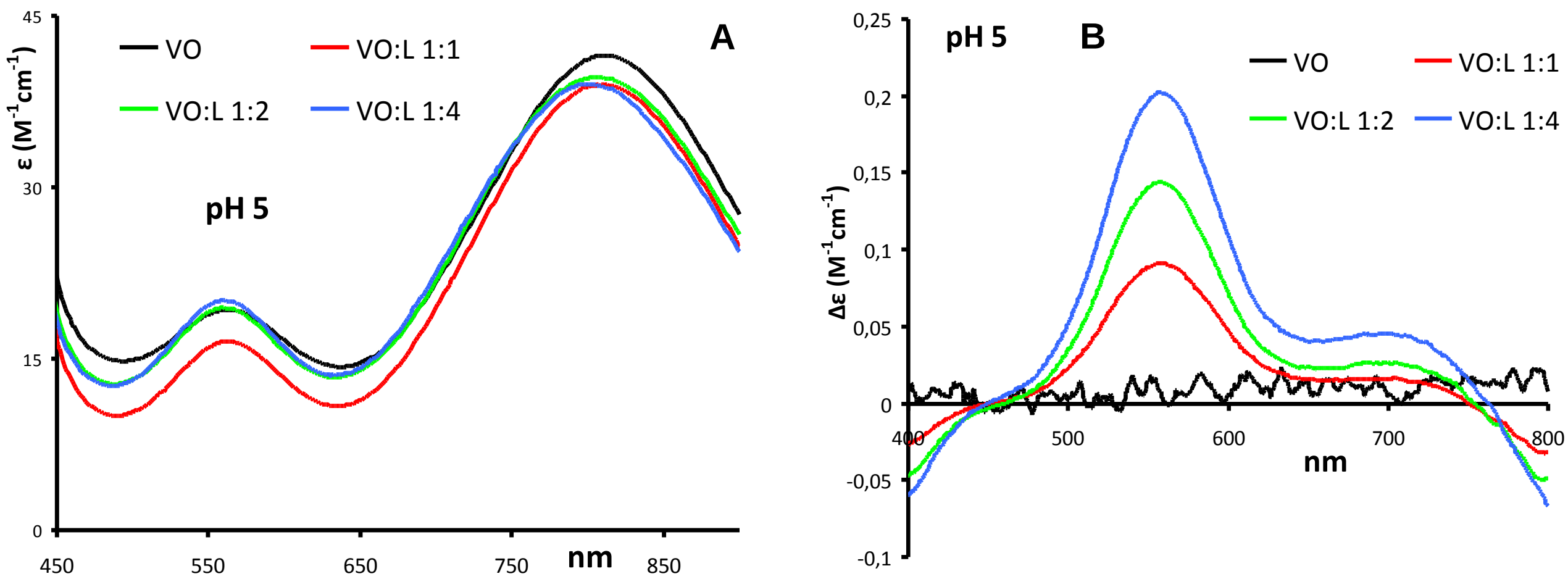

Figure S39. Spectra of solutions containing [$V^{IV}O(acac)_2$] and additions of Asp (L) at pH = 4.96 ± 0.06 and c_V ~2.0 mM. The indicated VO:L ratios correspond to [$V^{IV}O(acac)_2$]:Asp molar ratios. (A) Absorption spectra in the range 450-900 nm; (B) circular dichroism spectra in the range 400-800 nm. Note that the black spectrum was recorded with a solution of [$V^{IV}O(acac)_2$], where no CD bands are expected to exist.

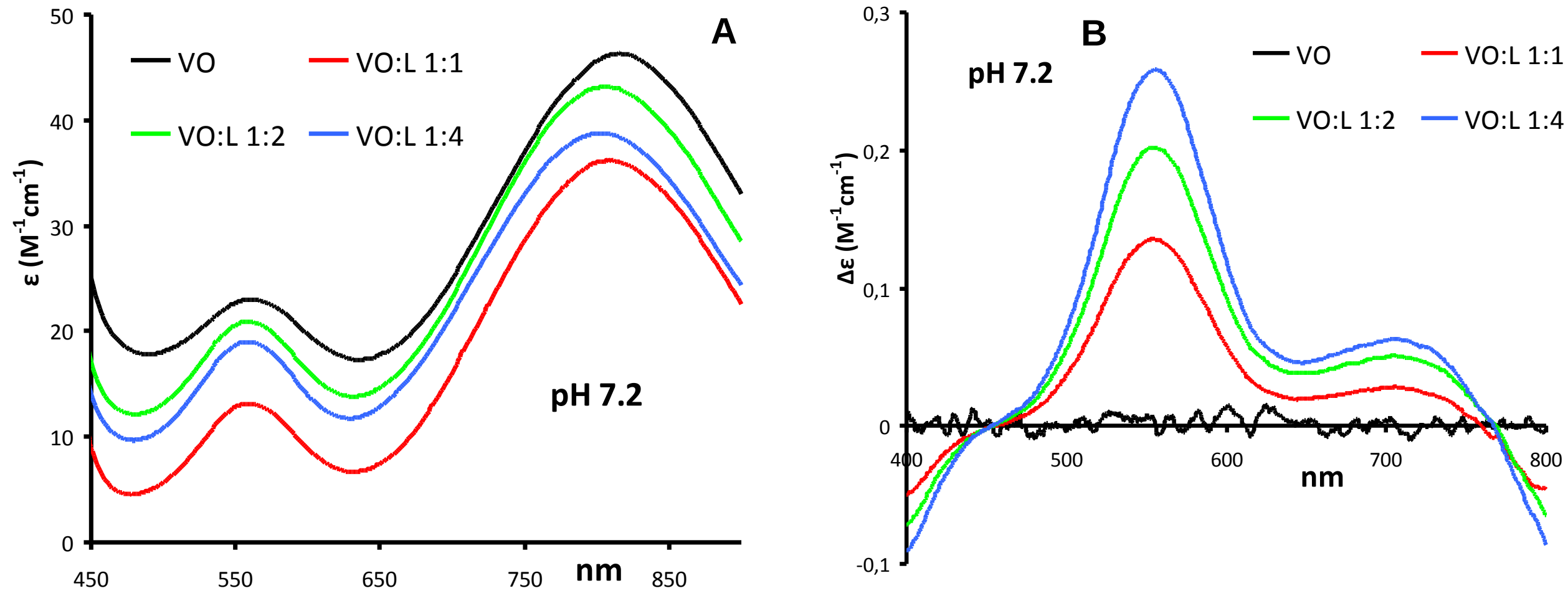

Figure S40. Spectra of solutions containing [$V^{IV}O(acac)_2$] and additions of Asp (L) at pH = 7.20 ± 0.01 and c_V ~2.0 mM. The indicated VO:L ratios correspond to [$V^{IV}O(acac)_2$]:Asp molar ratios. (A) Absorption spectra in the range 450-900 nm; (B) circular dichroism spectra in the range 400-800 nm. Note that the black spectrum was recorded with a solution of [$V^{IV}O(acac)_2$], where no CD bands are expected to exist.

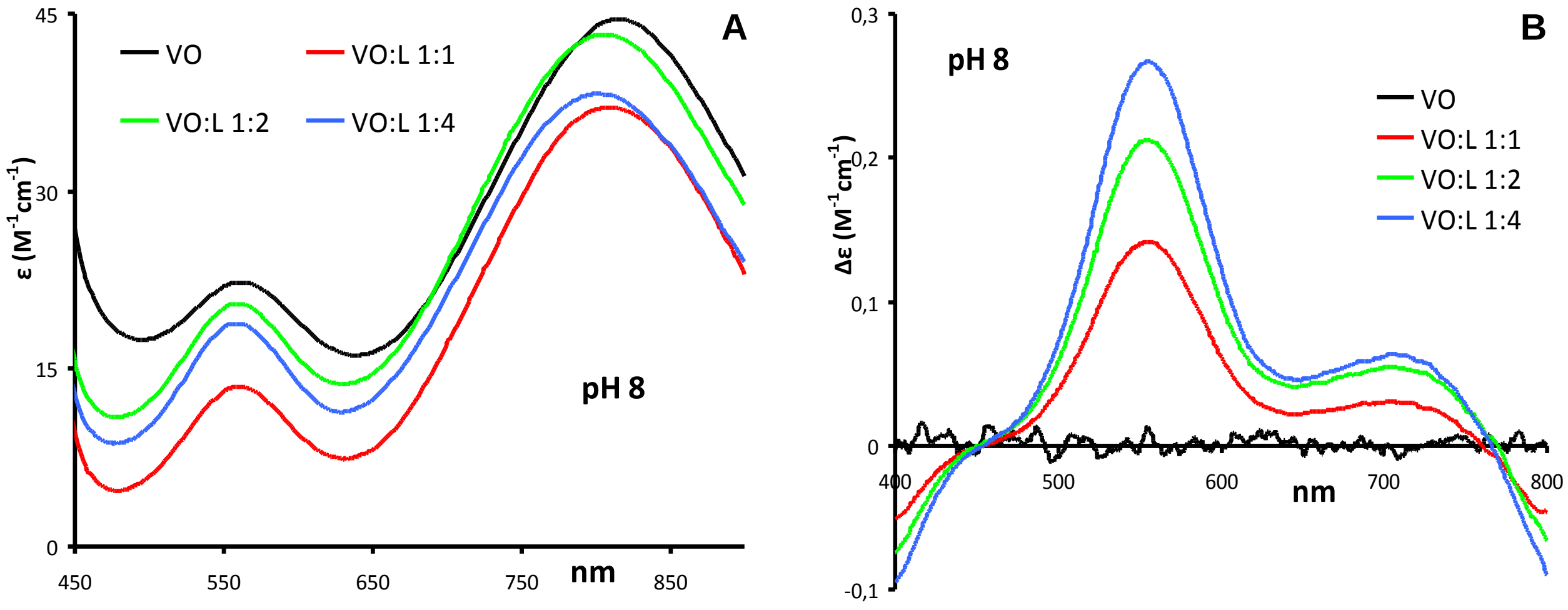

Figure S41. Spectra of solutions containing [$V^{IV}O(acac)_2$] and additions of Asp (L) at pH = 8.09 ± 0.04 and c_V ~2.0 mM. The indicated VO:L ratios correspond to [$V^{IV}O(acac)_2$]:Asp molar ratios. (A) Absorption spectra in the range 450-900 nm; (B) circular dichroism spectra in the range 400-800 nm. Note that the black spectrum was recorded with a solution of [$V^{IV}O(acac)_2$], where no CD bands are expected to exist.

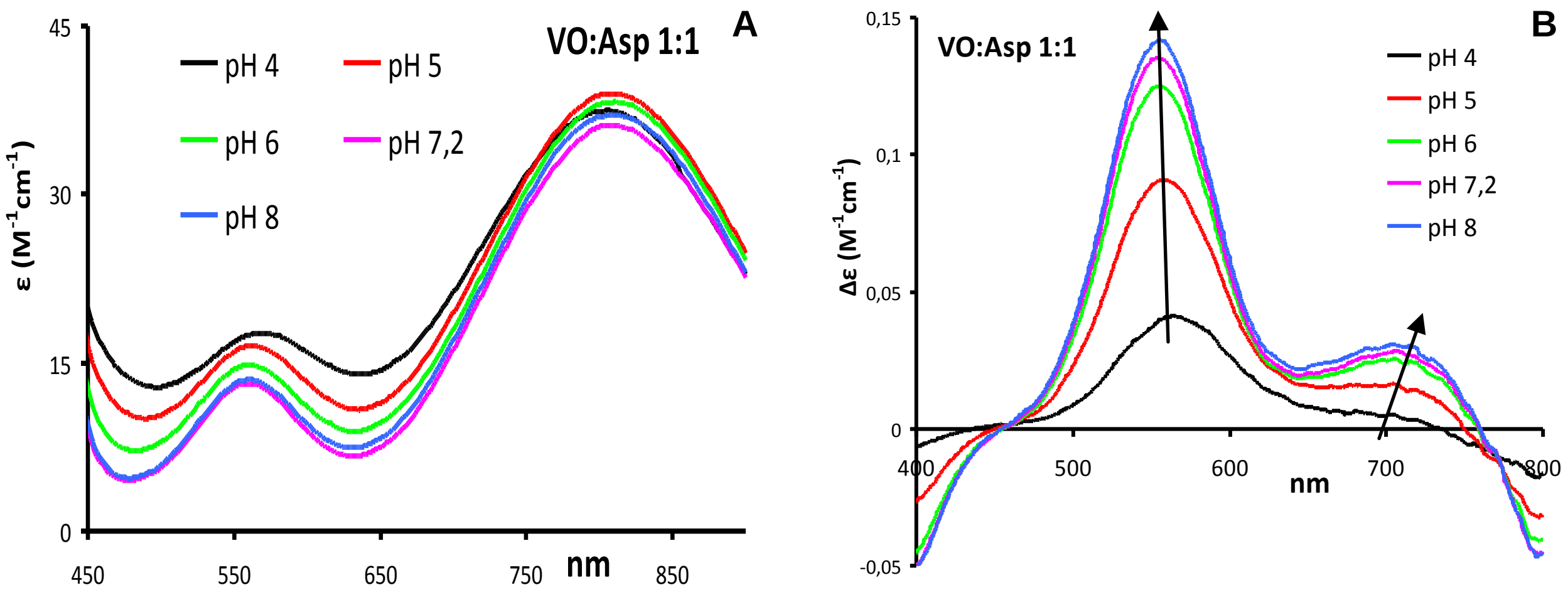

Figure S42. Spectra of solutions containing $[V^{IV}O(acac)_2]$ and Asp (L) with a 1:1 $[V^{IV}O(acac)_2]$:Asp molar ratio and c_V ~2.0 mM at several pH values. These spectra correspond to those included in Figures S38-S41. (A) Absorption spectra in the range 450-900 nm; (B) circular dichroism spectra in the range 400-800 nm. The changes in the position of the λ_{max} of the bands with $\Delta\varepsilon > 0$ as the amount of Asp is increased are indicated by arrows.

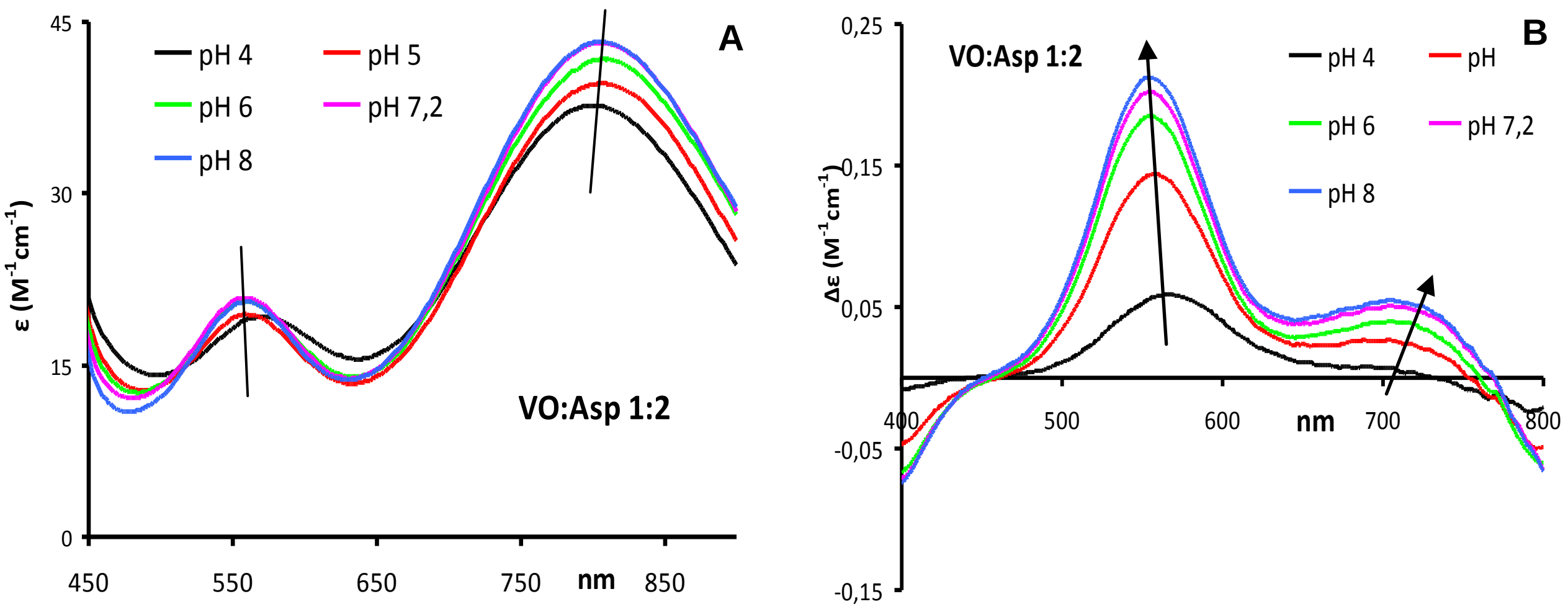

Figure S43. Spectra of solutions containing [$V^{IV}O(acac)_2$] and Asp (L) with a 1:2 [$V^{IV}O(acac)_2$]:Asp molar ratio and c_V ~2.0 mM at several pH values. These spectra correspond to those included in Figures S38-S41. (A) Absorption spectra in the range 450-900 nm; (B) circular dichroism spectra in the range 400-800 nm. The changes in the position of the λ_{max} of the bands with $\Delta\varepsilon > 0$ as the amount of Asp is increased are indicated by arrows.

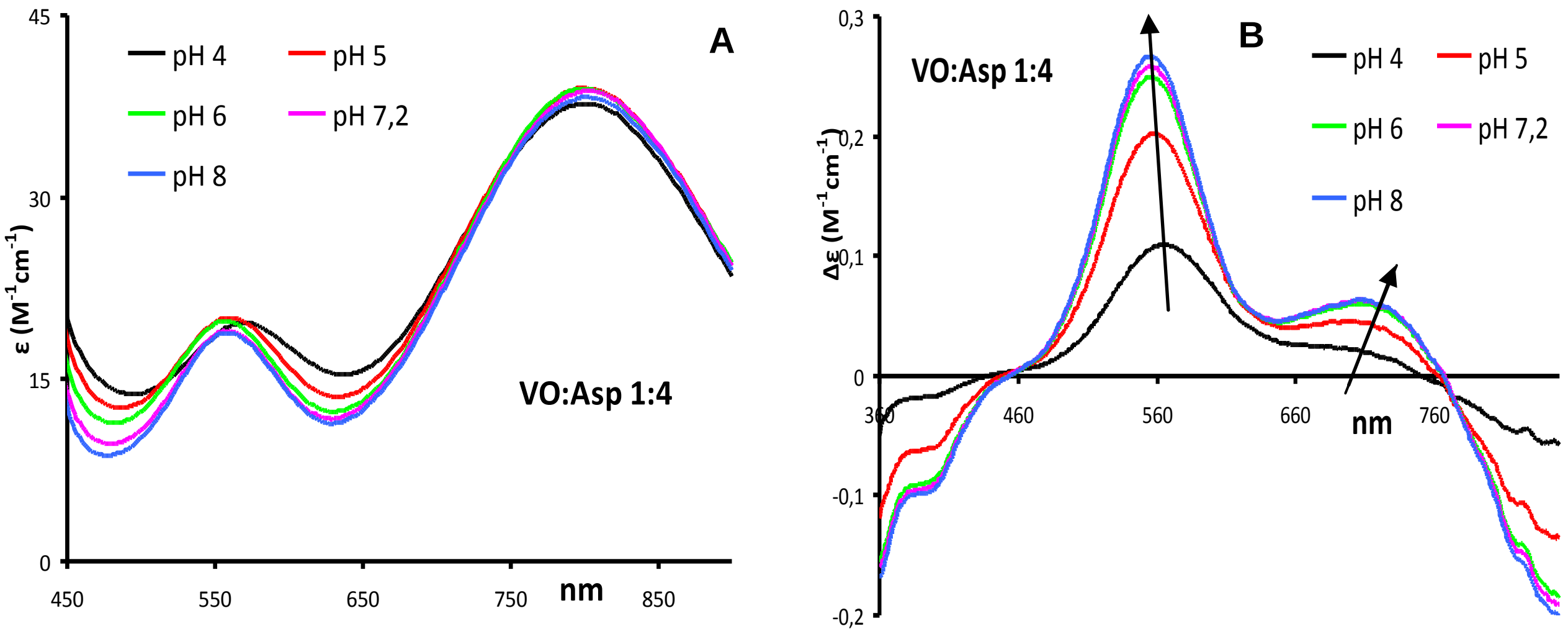

Figure S44. Spectra of solutions containing [$V^{IV}O(acac)_2$] and Asp (L) with a 1:4 [$V^{IV}O(acac)_2$]:Asp molar ratio and c_V ~2.0 mM at several pH values. These spectra correspond to those included in Figures S38-S41. (A) Absorption spectra in the range 450-900 nm; (B) circular dichroism spectra in the range 400-800 nm. The changes in the position of the λ_{max} of the bands with $\Delta\varepsilon > 0$ as the amount of Asp is increased are indicated by arrows.

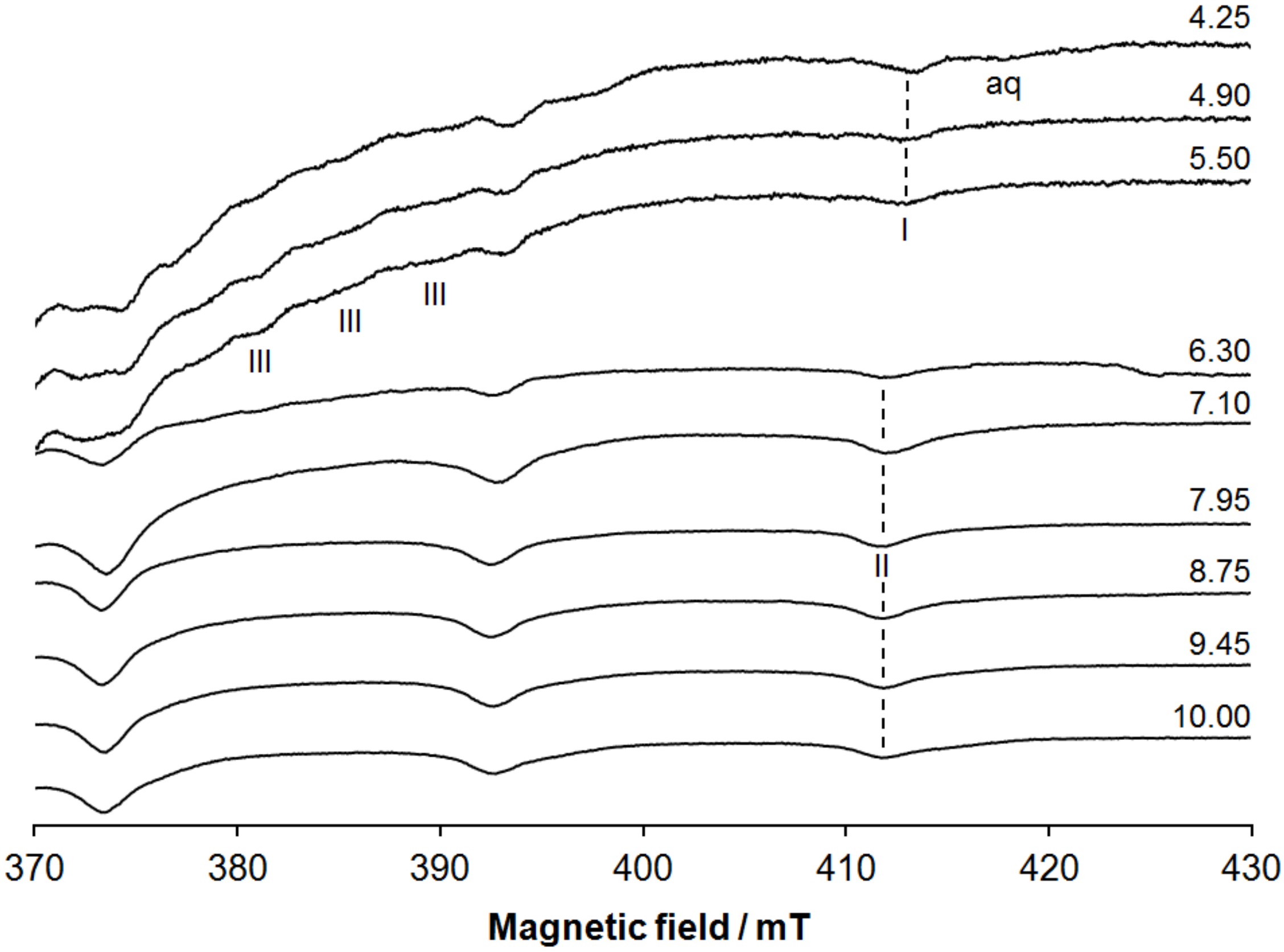

Figure S45. Anisotropic EPR spectra of solutions containing $V^{IV}O^{2+}$, acac and $P_2O_7^{4-}$ with molar ratio 1:1:1 and c_V = 2.0 × 10^{-3} M at various pH values. The resonances of these species are indicated: **aq**, $[V^{IV}O(H_2O)_5]^{2+}$; **I**, $[V^{IV}O(acac)(HP_2O_7)]^{2-}$ plus $[V^{IV}O(HP_2O_7)(H_2O)_2]^{-}$; **II**, $[V^{IV}O(acac)(P_2O_7)]^{3-}$; **III**, $[(V^{IV}O)_3(P_2O_7)_3]^{6-}$. The resonances of the mixed-ligand species $[V^{IV}O(acac)(HP_2O_7)]^{2-}$ / $[V^{IV}O(acac)(P_2O_7)]^{3-}$ are also denoted with the dotted line.

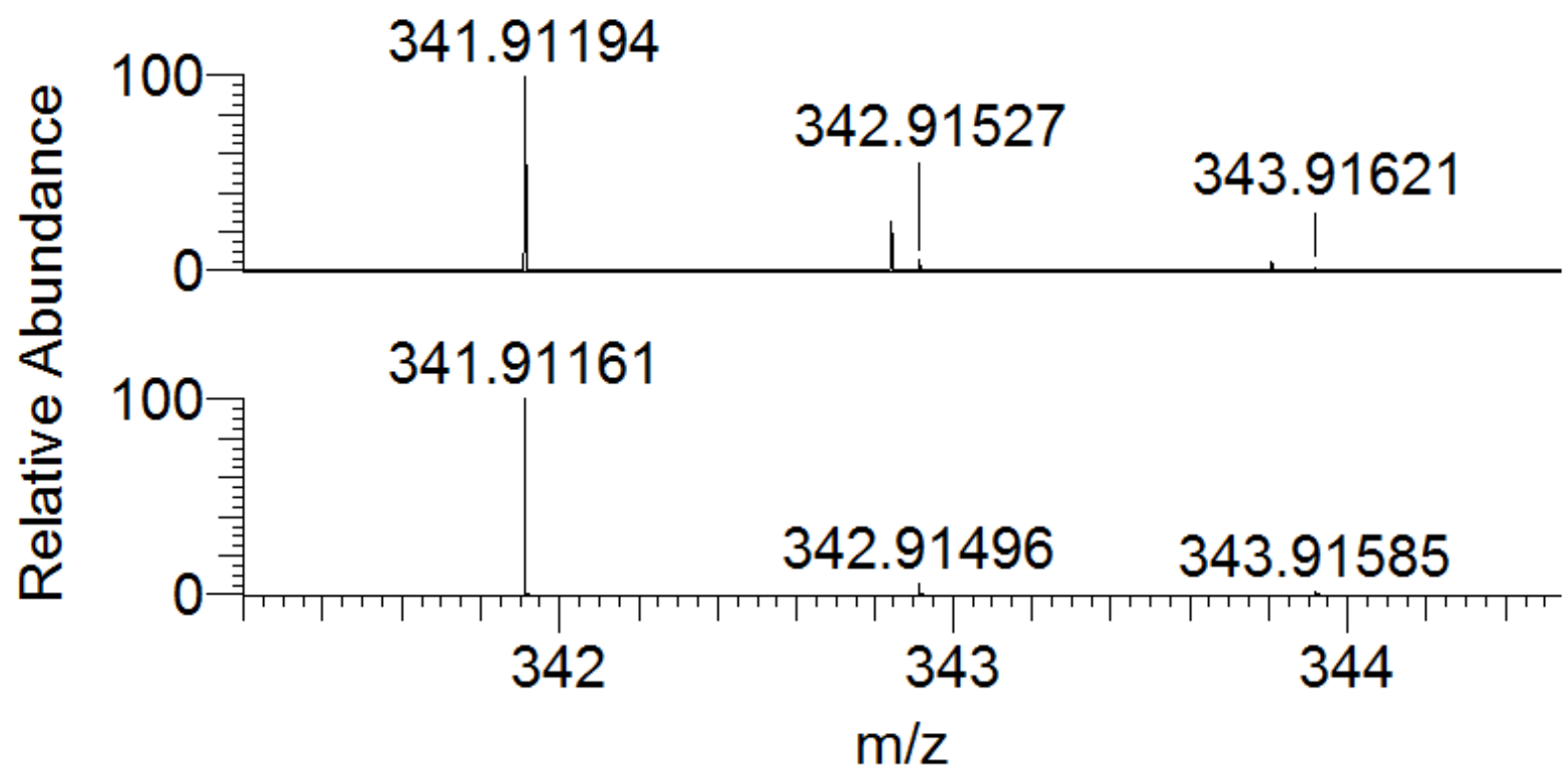

Figure S46. Experimental (above) and calculated (below) isotopic pattern for the peak of $[V^{IV}O(acac)(P_2O_7)+2H]^-$ revealed at m/z = 341.9119 in the ESI-MS(–) spectrum recorded on the $V^{IV}O^{2+}/acac/P_2O_7^{4-}$ system with molar ratio 1:1:5 (ultrapure LC-MS water, pH = 9.00, $c_V = 5.0 \times 10^{-6}$ M).

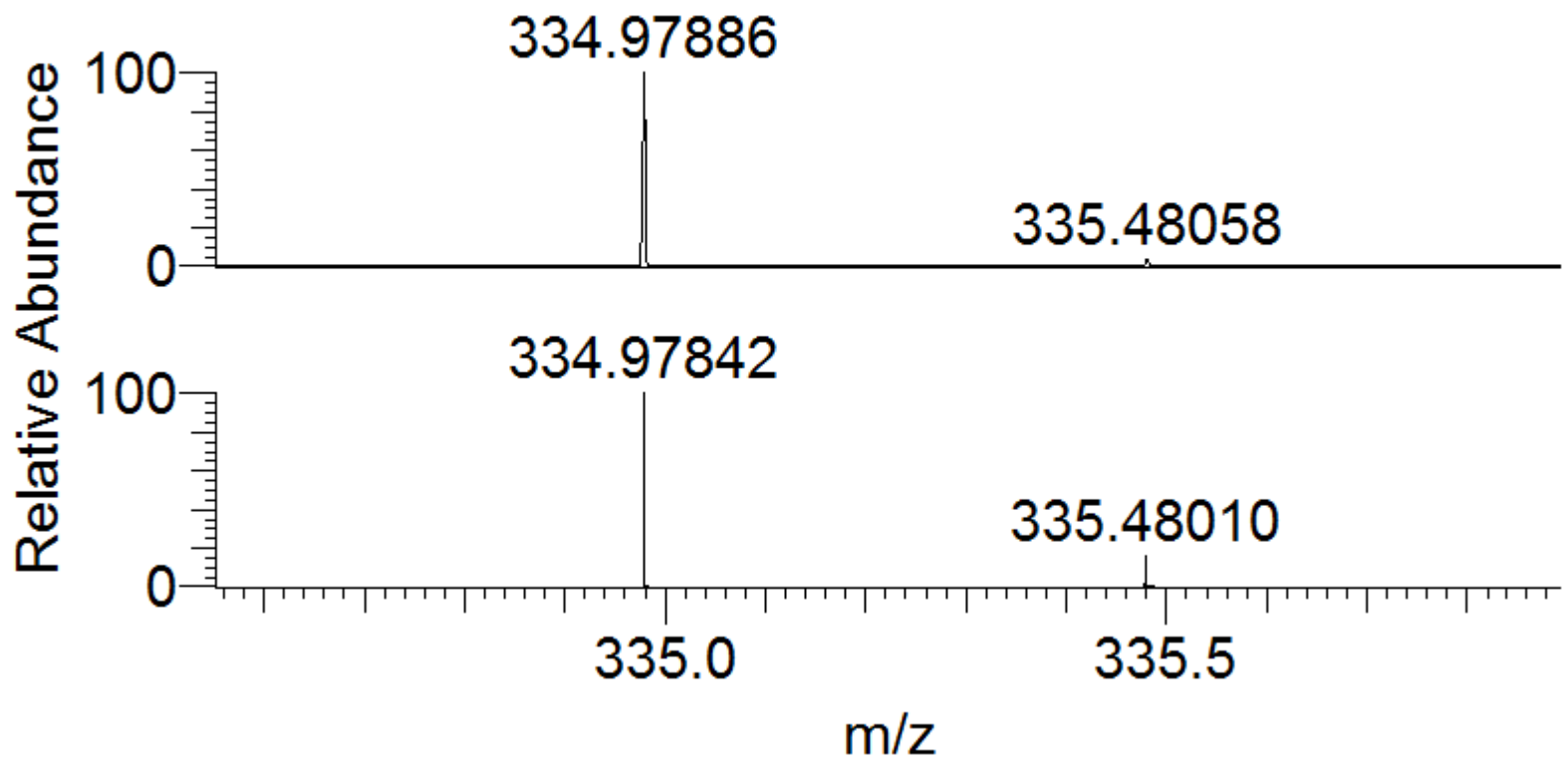

Figure S47. Experimental (above) and calculated (below) isotopic pattern for the peak of $[V^{IV}O(acac)(ATP)+H]^{2-}$ revealed at m/z = 334.9789 in the ESI-MS(–) spectrum recorded on the $V^{IV}O^{2+}$/acac/ATP system with molar ratio 1:1:10 (ultrapure LC-MS water, pH = 5.00, $c_V = 5.0 \times 10^{-6}$ M).

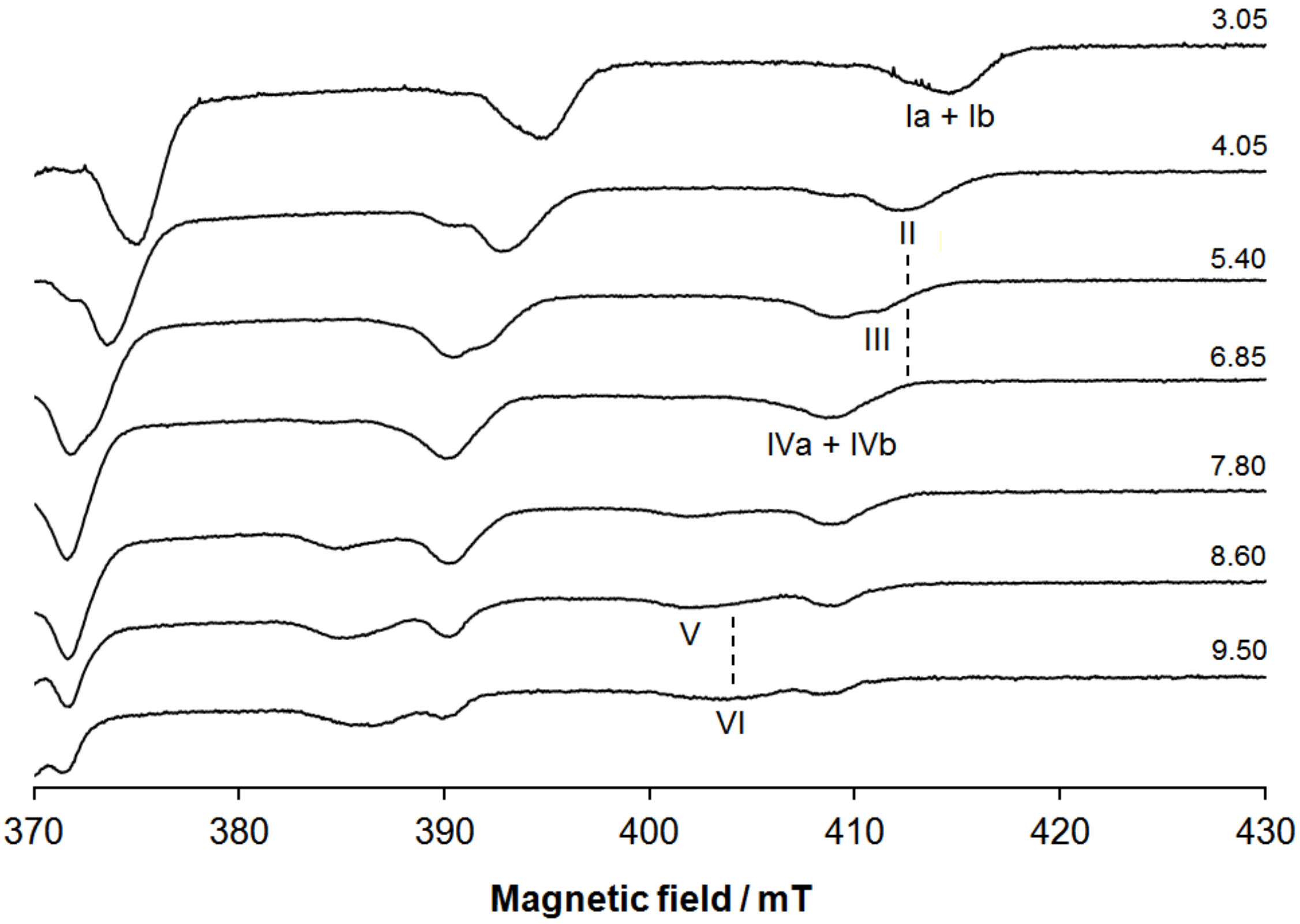

Figure S48. Anisotropic EPR spectra of solutions containing $V^{IV}O^{2+}$, acac and GSH with molar ratio 1:1:25 and c_V = 2.0 × 10^{-3} M at various pH values. **Ia + Ib**, $[V^{IV}O(H_2GSH)]^{x+}$ with coordination (COO^-, COO^-) of GSH and $[V^{IV}O(acac)]^+$; **II**, $[V^{IV}O(acac)(HGSH)]^-$ with coordination (NH_2, COO^-) of GSH; **III**, $[V^{IV}O(GSH)]^-$ with coordination (NH_2, COO^-); **IVa + IVb**, $[V^{IV}O(HGSH)_2]^{2-}$ with coordination 2 × (NH_2, COO^-) and $[V^{IV}O(acac)_2]$; **V**, $[V^{IV}O(GSH)(GSHH_{-1})]^{4-}$ with coordination (NH_2, COO^-); (N_{amide}^-, S^-) of GSH; **VI**, $[V^{IV}O(acac)(GSHH_{-1})]^{3-}$ with coordination (N_{amide}^-, S^-) of GSH. The resonances of the mixed species $[V^{IV}O(acac)(HGSH)]^-$ / $[V^{IV}O(GSH)(GSHH_{-1})]^{3-}$ are also denoted with the dotted line.

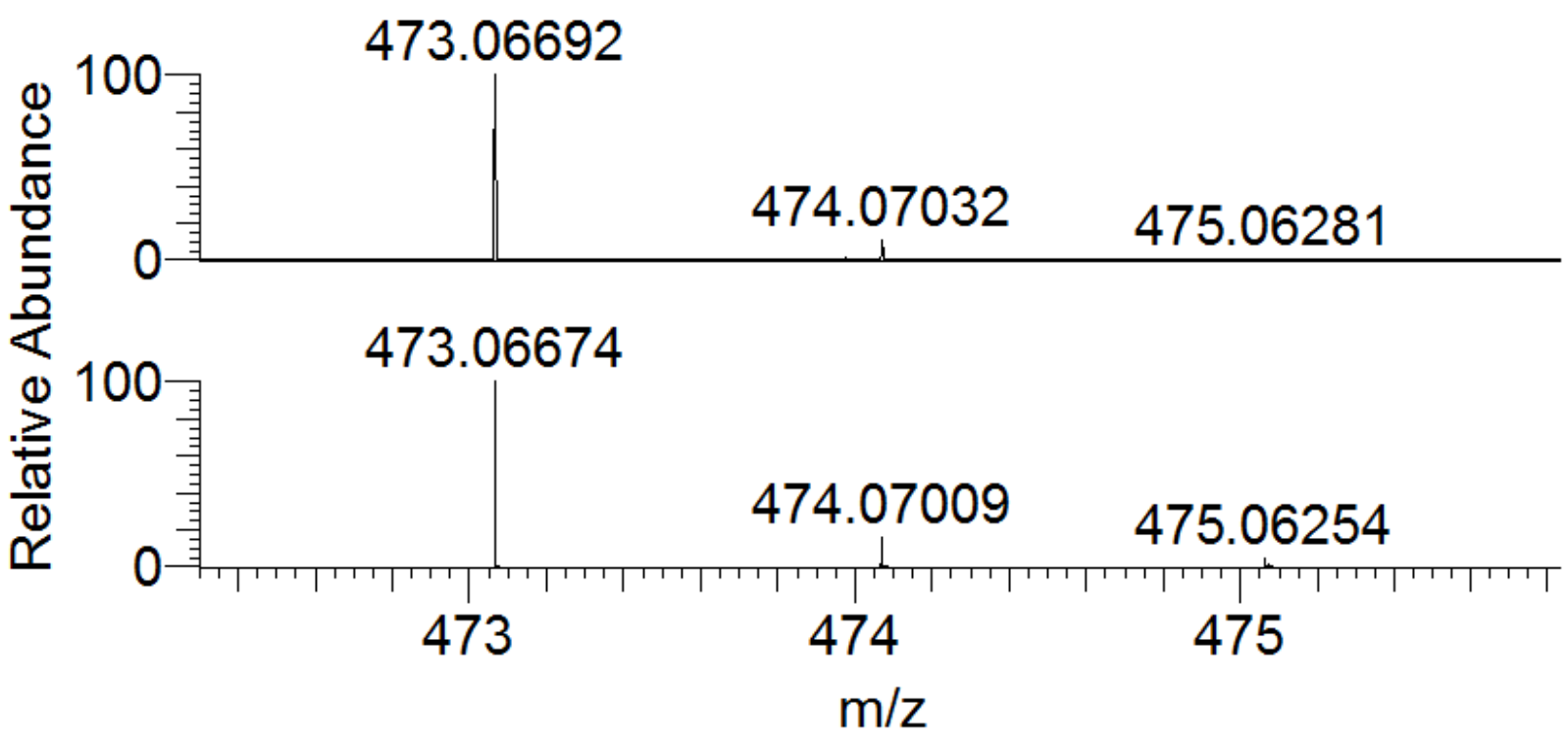

Figure S49. Experimental (above) and calculated (below) isotopic pattern for the peak of $[V^{IV}O(acac)(GSH)+3H]^+$ revealed at m/z = 473.0669 in the ESI-MS(+) spectrum recorded on the $V^{IV}O^{2+}$/acac/GSH system with molar ratio 1:1:25 (ultrapure LC-MS water, pH = 4.80, $c_V = 5.0 \times 10^{-6}$ M).

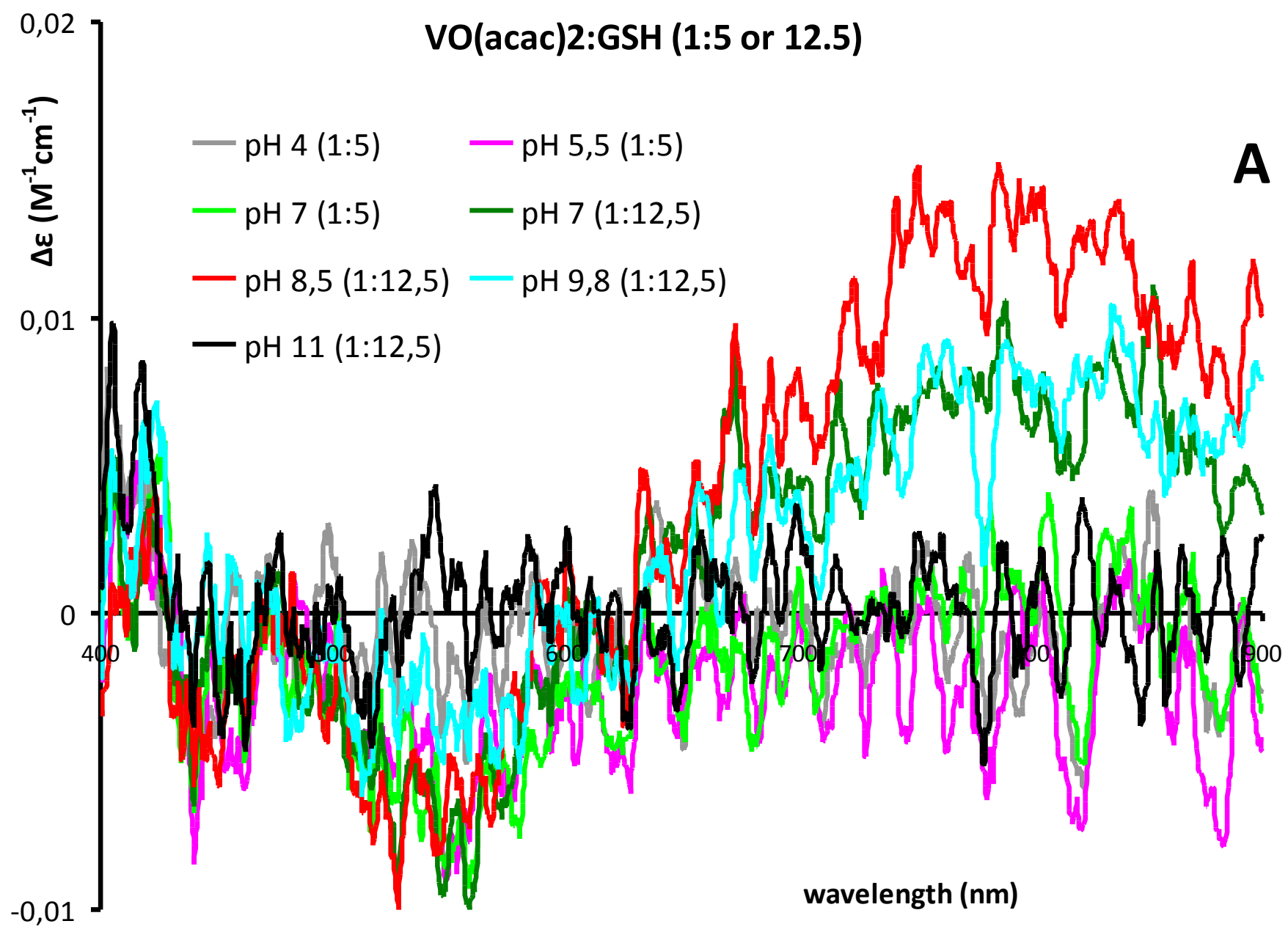

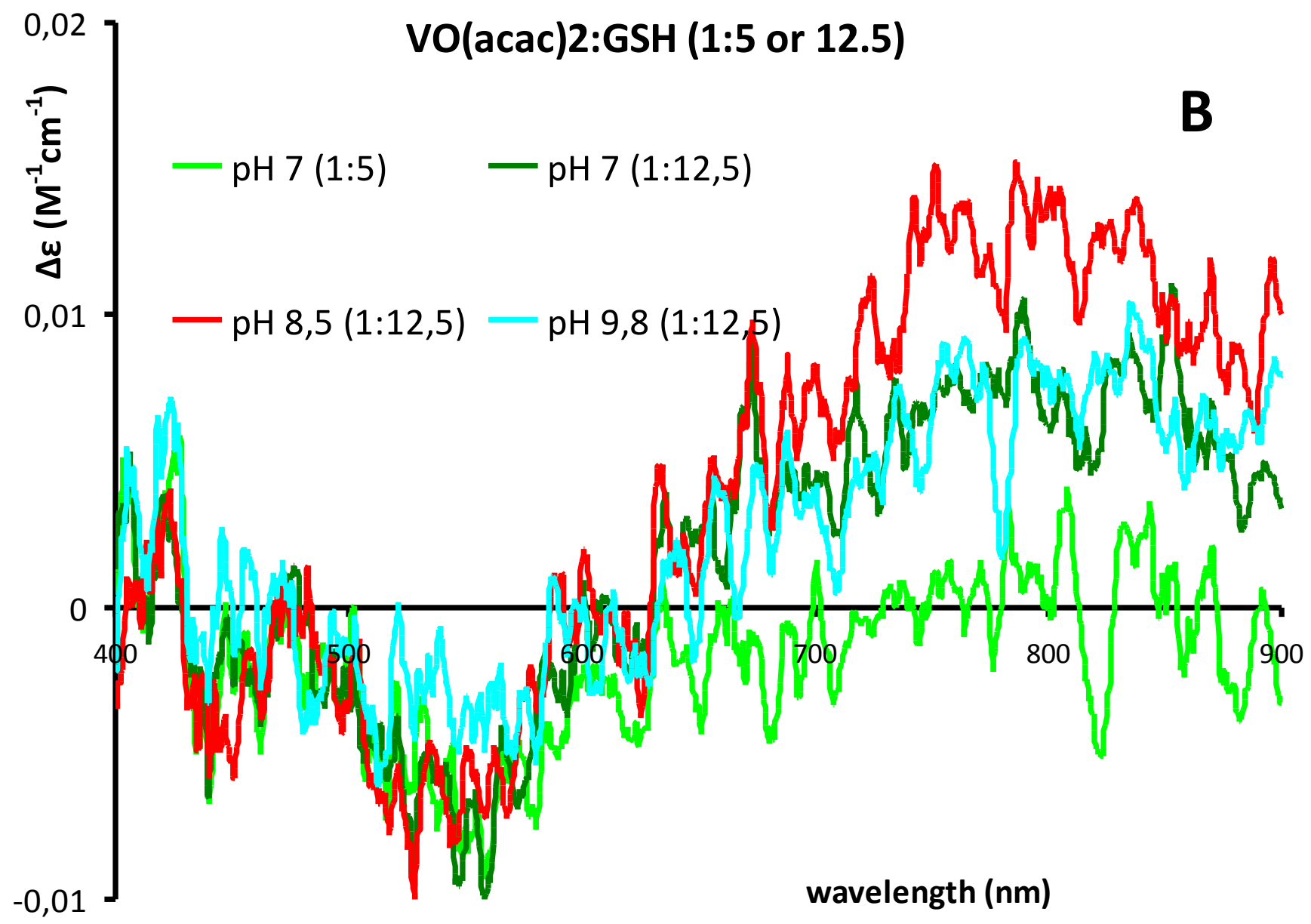

Figure S50. Circular dichroism spectra measured for solutions containing [$V^{IV}O(acac)_2$] and GSH with a 2.0 cm path length cell, c_V ~2.0 mM at several pH values. Molar ratios [$V^{IV}O(acac)_2$]:GSH of either 1:5 or of 1:12.5 were used. Because the induced CD spectra are very weak, the signal to noise ratio is low, and therefore the spectra appear very noisy. (A) CD spectra measured at the indicated pH values; (B) selection of some spectra included in (A).

Table S2. Bioligand concentration used to model the speciation of $V^{IV}O^{2+}$ ion in blood serum at pH 7.4 considering addition of $[V^{IV}O(acac)_2]$ in the range 1 nM-1000 μM.

Bioligand	Abbreviation	Concentration	Reference
Albumin	HSA	639.4 μM	1
Citrate	citr	99.0 μM	2
Cysteine	Cys	33.0 μM	2
Histidine	His	77.0 μM	2
Immunoglobulin G	IgG	101.0 μM	1
Inorganic phosphate	P_i	1.10 mM	2
Lactate	lact	1.51 mM	2
Oxalate	ox	9.20 μM	2
Transferrin	HTf	16.8 μM [a]	1

[a] For transferrin a total concentration of 29.9 μM were considered from ref. 1. Since 30% of HTf binds Fe^{3+}, this value corresponds to a protein concentration of 16.8 μM with 33.6 μM of available sites taking into account that there are two sites for $V^{IV}O^{2+}$ ion in the *N*-terminal and *C*-terminal region.

Table S3. Stability constants used to model the speciation of $V^{IV}O^{2+}$ ion in blood serum at pH 7.4 considering addition of $[V^{IV}O(acac)_2]$ in the range 1 nM-1000 μM.

Species	Reference
Binary complexes $V^{IV}O$–acac	This work
$(V^{IV}O)$(HSA)	3
Binary complexes $V^{IV}O$–citr	4
Ternary complexes $V^{IV}O$–acac–citr	This work
Binary complexes $V^{IV}O$–Cys	5
Ternary complexes $V^{IV}O$–acac–Cys	This work [a]
Binary complexes $V^{IV}O$–His	6
Ternary complexes $V^{IV}O$–acac–His	This work
$(V^{IV}O)$(IgG)	7
Binary complexes $V^{IV}O$–P_i	8
Binary complexes $V^{IV}O$–lact	9
Ternary complexes $V^{IV}O$–acac–lact	This work
Binary complexes $V^{IV}O$–ox	10
Ternary complexes $V^{IV}O$–acac–ox	This work
$(V^{IV}O)$(HTf) and $(V^{IV}O)_2$(HTf)	3
$[V^{IV}O(OH)]^+$ and $[(V^{IV}O)_2(OH)_2]^{2+}$	11
$[V^{IV}O(OH)_3]^-$ and $[(V^{IV}O)_2(OH)_5]^-$	12

[a] Species with low stability on the basis of the EPR data.

Table S4. Bioligand concentration used to model the speciation of $V^{IV}O^{2+}$ ion in RBC cytosol at pH 7.2 considering addition of $[V^{IV}O(acac)_2]$ in the range 1 nM-1000 μM.

Bioligand	Abbreviation	Concentration	Reference
Adenosine 5'-triphosphate	ATP	1.35 mM	13
Aspartate	Asp	306.0 μM	13
Citrate	citr	50.0 μM	This work [a]
Cysteine	Cys	33.0 μM	13
Glutathione reduced	GSH	2.23 mM	13
Glutathione oxidized	GSSG	3.6 μM	13
Hemoglobyn	Hb	5.10 mM	13
Histidine	His	86.0 μM	13
Inorganic phosphate	P_i	480.0 μM	13
Lactate	lact	0.932 μM	13
Nicotinamide adenine dinucleotide	NAD^+	49.0 μM	13
Nicotinamide adenine dinucleotide phosphate	$NADP^+$	1.4 μM	13

[a] Considering that citrate is detected in erythrocytes by metabolomic analyses, consistent with the absence of a functional tricarboxylic acid cycle in these cells, for our model its intracellular concentration was set to 50.0 μM, within the low μM range expected for this metabolite according to ref. 14.

Table S5. Stability constants used to model the speciation of $V^{IV}O^{2+}$ ion in RBC cytosol at pH 7.2 considering addition of $[V^{IV}O(acac)_2]$ in the range 1 nM-1000 μM.

Species	Reference
Binary complexes $V^{IV}O$–acac	This work
Binary complexes $V^{IV}O$–ATP	15
Ternary complexes $V^{IV}O$–acac–ATP	This work
Binary complexes $V^{IV}O$–Asp	16
Ternary complexes $V^{IV}O$–acac–Asp	This work
Binary complexes $V^{IV}O$–citr	4
Ternary complexes $V^{IV}O$–acac–citr	This work [a]
Binary complexes $V^{IV}O$–Cys	5
Ternary complexes $V^{IV}O$–acac–Cys	This work
Binary complexes $V^{IV}O$–GSH	17
Binary complexes $V^{IV}O$–GSSG	18
$(V^{IV}O)(Hb)$	19
Binary complexes $V^{IV}O$–His	6
Ternary complexes $V^{IV}O$–acac–His	This work
Binary complexes $V^{IV}O$–P_i	8
Binary complexes $V^{IV}O$–lact	9
Ternary complexes $V^{IV}O$–acac–lact	This work
Binary complexes $V^{IV}O$–NAD^+	20
Binary complexes $V^{IV}O$–$NADP^+$	20
$[V^{IV}O(OH)]^+$ and $[(V^{IV}O)_2(OH)_2]^{2+}$	11
$[V^{IV}O(OH)_3]^-$ and $[(V^{IV}O)_2(OH)_5]^-$	12

[a] Species with low stability on the basis of the EPR data.

Table S6. Bioligand concentration used to model the speciation of $V^{IV}O^{2+}$ ion in cancer cells at pH 7.3 considering addition of $[V^{IV}O(acac)_2]$ in the range 1 nM-1000 μM.

Bioligand	Abbreviation	Concentration	Reference
Adenosine 5'-triphosphate	ATP	6.0 mM	21
Aspartate	Asp	1.0 mM	22
Citrate	citr	1.0 mM	23
Cysteine	Cys	50.0 μM	24
Diphosphate	P_2O_7	50 μM	25
Glutathione reduced	GSH	5.0 mM	26
Histidine	His	50.0 μM	27
Inorganic phosphate	P_i	5 mM	28
Lactate	lact	15.0 mM	29
Oxalate	ox	50.0 μM	30
Protein	Protein	5.0 mM	This work [a]

[a] A mean value of 5.0 mM was considered for the total concentration of proteins.

Table S7. Stability constants used to model the speciation of $V^{IV}O^{2+}$ ion in cancer cells at pH 7.3 considering addition of $[V^{IV}O(acac)_2]$ in the range 1 nM-1000 μM.

Species	Reference
Binary complexes $V^{IV}O$–acac	This work
Binary complexes $V^{IV}O$–ATP	15
Ternary complexes $V^{IV}O$–acac–ATP	This work
Binary complexes $V^{IV}O$–Asp	16
Ternary complexes $V^{IV}O$–acac–Asp	This work
Binary complexes $V^{IV}O$–citr	4
Ternary complexes $V^{IV}O$–acac–citr	This work
Binary complexes $V^{IV}O$–Cys	5
Binary complexes $V^{IV}O$–P_2O_7	31
Ternary complexes $V^{IV}O$–acac–P_2O_7	This work
Binary complexes $V^{IV}O$–GSH	17
Binary complexes $V^{IV}O$–His	6
Binary complexes $V^{IV}O$–P_i	8
Ternary complexes $V^{IV}O$–acac–His	This work
Binary complexes $V^{IV}O$–lact	9
Ternary complexes $V^{IV}O$–acac–lact	This work
Binary complexes $V^{IV}O$–ox	10
Ternary complexes $V^{IV}O$–acac–ox	This work
$(V^{IV}O)$(Protein)	This work
$[V^{IV}O(OH)]^+$ and $[(V^{IV}O)_2(OH)_2]^{2+}$	11
$[V^{IV}O(OH)_3]^-$ and $[(V^{IV}O)_2(OH)_5]^-$	12

[a] For the adducts formed by $V^{IV}O^{2+}$ with proteins, $(V^{IV}O)$(Prot), a value of conditional binding constant (logβ) of 9.0 was considered acceptable for a non-specific binding of His-N.

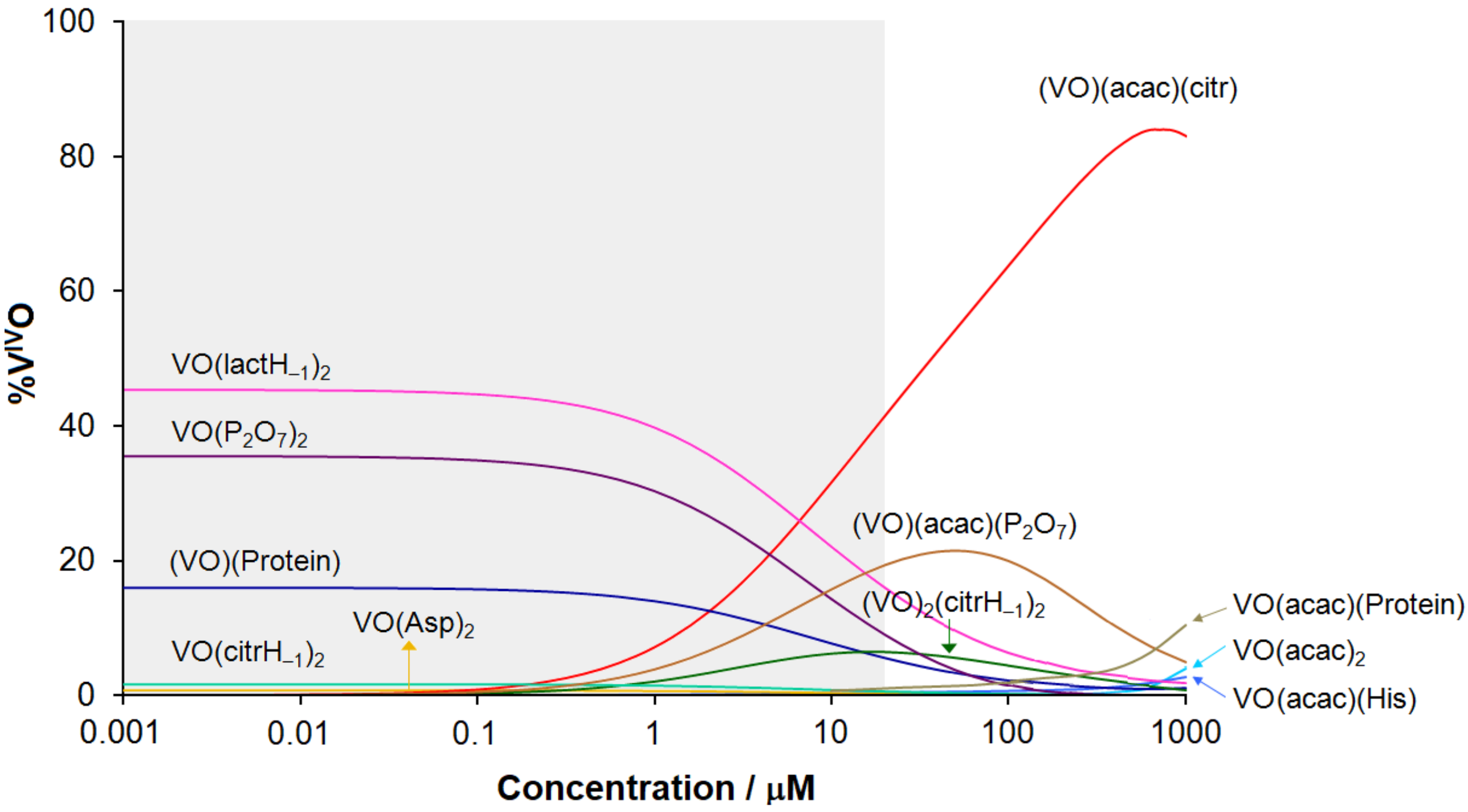

Figure S51. Calculated speciation in cancer cells at pH 7.3 considering addition of [$V^{IV}O(acac)_2$] in the range 1 nM-1000 µM. The V concentration range below 20 µM is shown with a gray background, even though it should be considered that, upon administration by intraperitoneal or intratumoral injections of VCs, the V concentration reached inside the cancer cells may possibly be much higher than 20 µM. The concentration of the bioligands was taken from refs. 21, 22, 23, 24, 25, 26, 27, 28, 29, 30, while for proteins a total concentration of 5 mM was fixed. The stability constants used to model the speciation are reported in Table S7, but – compared to Figure 21 of the main text – the species $V^{IV}O(acac)(Protein)$ was added with an estimated value of the conditional stability constant (logβ) of 14.5.

References

(1) Schaller, J.; Gerber, S.; Kämpfer, U.; Lejon, S.; Trachsel, C. *Human Blood Plasma Proteins: Structure and Function*. John Wiley & Sons Ltd.: Chichester, 2008.

(2) Harris, W. R. Equilibrium model for speciation of aluminum in serum. *Clin. Chem.* **1992**, *38*, 1809-18.

(3) Sanna, D.; Buglyó, P.; Micera, G.; Garribba, E. A quantitative study of the biotransformation of insulin-enhancing VO^{2+} compounds. *JBIC, J. Biol. Inorg. Chem.* **2010**, *15*, 825-839.

(4) Lodyga-Chruscinska, E.; Sanna, D.; Garribba, E.; Micera, G. Potentiometric, spectroscopic, electrochemical and DFT characterization of oxovanadium(IV) complexes formed by citrate and tartrates in aqueous solution at high ligand to metal molar ratios: the effects of the trigonal bipyramidal distortion in bis-chelated species and biological implications. *Dalton Trans.* **2008**, 4903-4916.

(5) Costa Pessoa, J.; Vilas Boas, L. F.; Gillard, R. D. Oxovanadium(IV) and amino acids–IV. The systems L-cysteine or D-penicillamine + VO^{2+}; a potentiometric and spectroscopic study. *Polyhedron* **1990**, *9*, 2101-2125.

(6) Costa Pessoa, J.; Luz, S. M.; Cavaco, I.; Gillard, R. D. Oxovanadium(IV) and amino acids–VII. The system L-histidine + VO^{2+}; a self-consistent potentiometric and spectroscopic study. *Polyhedron* **1994**, *13*, 3177-3198.

(7) Sanna, D.; Micera, G.; Garribba, E. Interaction of VO^{2+} Ion and Some Insulin-Enhancing Compounds with Immunoglobulin G. *Inorg. Chem.* **2011**, *50*, 3717-3728.

(8) Kiss, T.; Kiss, E.; Micera, G.; Sanna, D. The formation of ternary complexes between $VO(maltolate)_2$ and small bioligands. *Inorg. Chim. Acta* **1998**, *283*, 202-210.

(9) Micera, G.; Sanna, D.; Dessì, A.; Kiss, T.; Buglyó, P. Complex-forming properties of α-hydroxycarboxylic acids with oxovanadium(IV) ion. *Gazz. Chim. Ital.* **1993**, *123*, 573-577.

(10) Buglyo, P.; Kiss, E.; Fabian, I.; Kiss, T.; Sanna, D.; Garribba, E.; Micera, G. Speciation and NMR relaxation studies of VO(IV) complexes with several O-donor containing ligands: oxalate, malonate, maltolate and kojate. *Inorg. Chim. Acta* **2000**, *306*, 174-183.

(11) Henry, R. P.; Mitchell, P. C. H.; Prue, J. E. Hydrolysis of the oxovanadium(IV) ion and the stability of its complexes with the 1,2-dihydroxybenzenato(2–) ion. *J. Chem. Soc., Dalton Trans.* **1973**, 1156-1159.

(12) (a) Komura, A.; Hayashi, M.; Imanaga, H. Hydrolytic Behavior of Oxovanadium(IV) Ions. *Bull. Chem. Soc. Jpn.* **1977**, *50*, 2927-2931. (b) Vilas Boas, L.; Costa Pessoa, J. Vanadium. In *Comprehensive Coordination Chemistry*; Wilkinson, G., Gillard, R., McCleverty, J., Ed.;

Pergamon: Oxford, 1987; Vol. 3, pp 453-583. (c) Costa Pessoa, J. Thirty years through vanadium chemistry. *J. Inorg. Biochem.* **2015**, *147*, 4-24.

(13) *Williams Hematology, Eighth Edition*. The McGraw-Hill Companies, Inc.: China, 2010.

(14) Nemkov, T.; Sun, K.; Reisz, J. A.; Yoshida, T.; Dunham, A.; Wen, E. Y.; Wen, A. Q.; Roach, R. C.; Hansen, K. C.; Xia, Y.; D'Alessandro, A. Metabolism of Citrate and Other Carboxylic Acids in Erythrocytes As a Function of Oxygen Saturation and Refrigerated Storage. *Front. Med.* **2017**, *4*, 175.

(15) Alberico, E.; Dewaele, D.; Kiss, T.; Micera, G. Oxovanadium(IV) complexation by adenosine 5′-di-and -tri-phosphate and nucleotide building blocks. *J. Chem. Soc., Dalton Trans.* **1995**, 425-430.

(16) Costa Pessoa, J.; Marques, R. L.; Vilas Boas, L. F.; Gillard, R. D. Oxovanadium(IV) and amino acids–III. The system L-aspartic acid + VO^{2+}; A potentiometric and spectroscopic study. *Polyhedron* **1990**, *9*, 81-98.

(17) Costa Pessoa, J.; Tomaz, I.; Kiss, T.; Kiss, E.; Buglyó, P. The systems $V^{IV}O^{2+}$-glutathione and related ligands: a potentiometric and spectroscopic study. *JBIC, J. Biol. Inorg. Chem.* **2002**, *7*, 225-240.

(18) Costa Pessoa, J.; Tomaz, I.; Kiss, T.; Buglyó, P. The system VO^{2+}+oxidized glutathione: a potentiometric and spectroscopic study. *J. Inorg. Biochem.* **2001**, *84*, 259-270.

(19) Sanna, D.; Serra, M.; Micera, G.; Garribba, E. Interaction of Antidiabetic Vanadium Compounds with Hemoglobin and Red Blood Cells and Their Distribution between Plasma and Erythrocytes. *Inorg. Chem.* **2014**, *53*, 1449-1464.

(20) Micera, G.; Sanna, D.; Kiss, E.; Garribba, E.; Kiss, T. Oxovanadium(IV) complexes of phosphates of biological relevance: NAD^+, $NADP^+$ and thiamine mono- and diphosphate. *J. Inorg. Biochem.* **1999**, *75*, 303-309.

(21) Qian, Y.; Wang, X.; Li, Y.; Cao, Y.; Chen, X. Extracellular ATP a New Player in Cancer Metabolism: NSCLC Cells Internalize ATP In Vitro and In Vivo Using Multiple Endocytic Mechanisms. *Mol. Cancer Res.* **2016**, *14*, 1087-1096.

(22) Garcia-Bermudez, J.; Baudrier, L.; La, K.; Zhu, X. G.; Fidelin, J.; Sviderskiy, V. O.; Papagiannakopoulos, T.; Molina, H.; Snuderl, M.; Lewis, C. A.; Possemato, R. L.; Birsoy, K. Aspartate is a limiting metabolite for cancer cell proliferation under hypoxia and in tumours. *Nat. Cell Biol.* **2018**, *20*, 775-781.

(23) Giskeødegård, G. F.; Bertilsson, H.; Selnæs, K. M.; Wright, A. J.; Bathen, T. F.; Viset, T.; Halgunset, J.; Angelsen, A.; Gribbestad, I. S.; Tessem, M.-B. Spermine and Citrate as Metabolic Biomarkers for Assessing Prostate Cancer Aggressiveness. *PLoS One* **2013**, *8*, e62375.

(24) Zhuang, M.; Hou, Z.; Chen, P.; Liang, G.; Huang, G. Introducing charge tag via click reaction in living cells for single cell mass spectrometry. *Chem. Sci.* **2020**, *11*, 7308-7312.

(25) (a) Qiu, D.; Wilson, M. S.; Eisenbeis, V. B.; Harmel, R. K.; Riemer, E.; Haas, T. M.; Wittwer, C.; Jork, N.; Gu, C.; Shears, S. B.; Schaaf, G.; Kammerer, B.; Fiedler, D.; Saiardi, A.; Jessen, H. J. Analysis of inositol phosphate metabolism by capillary electrophoresis electrospray ionization mass spectrometry. *Nat. Commun.* **2020**, *11*, 6035. (b) Wimmer, J. L. E.; Kleinermanns, K.; Martin, W. F. Pyrophosphate and Irreversibility in Evolution, or why PPi Is Not an Energy Currency and why Nature Chose Triphosphates. *Front. Microbiol.* **2021**, *12*, 759359.

(26) Ortega, A. L.; Mena, S.; Estrela, J. M. Glutathione in Cancer Cell Death. *Cancers* **2011**, *3*, 1285-1310.

(27) Lee, C.-H.; Chen, I.-S.; Chao, Y.-J.; Hsu, H.-P. High plasma histidine concentration predicts a favorable prognosis in patients with ampullary cancer. *Am. J. Cancer Res.* **2025**, *15*, 4452-4469.

(28) (a) Ramirez, C. P.; Fiedler, D. Investigating the role of inorganic phosphate in tumor metabolism and metastasis. *Cancer Metab.* **2014**, *2*, P55. (b) Jennings, M. L. Role of transporters in regulating mammalian intracellular inorganic phosphate. *Front. Pharmacol.* **2023**, *14*, 1163442.

(29) (a) Pérez-Tomás, R.; Pérez-Guillén, I. Lactate in the Tumor Microenvironment: An Essential Molecule in Cancer Progression and Treatment. *Cancers* **2020**, *12*, 3244. (b) Sharma, D.; Singh, M.; Gupta, R.; Kumar, V.; Kumar, V.; Rani, R. Intervention on lactate in cancer: A promising approach for the development of cancer therapeutics. *Adv. Cancer Biol. - Metastasis* **2022**, *5*, 100058.

(30) Castellaro, A. M.; Tonda, A.; Cejas, H. H.; Ferreyra, H.; Caputto, B. L.; Pucci, O. A.; Gil, G. A. Oxalate induces breast cancer. *BMC Cancer* **2015**, *15*, 761.

(31) Buglyó, P.; Kiss, T.; Alberico, E.; Micera, G.; Dewaele, D. Oxovanadium(IV) complexes of di- and triphosphate. *J. Coord. Chem.* **1995**, *36*, 105-116.
